# A Systems Pharmacology-Based Study of the Molecular Mechanisms of San Cao Decoction for Treating Hypertension

**DOI:** 10.1155/2019/3171420

**Published:** 2019-07-02

**Authors:** Chongyang Ma, Changming Zhai, Tian Xu, Fang Lu, Shuang Zhang, Changxiang Li, Qingguo Wang, Fafeng Cheng, Xueqian Wang

**Affiliations:** ^1^School of Traditional Chinese Medicine, Capital Medical University, 10 Youanmenwai, Xitoutiao, Fengtai District, Beijing, China; ^2^School of Traditional Chinese Medicine, Beijing University of Chinese Medicine, 11 Beisanhuandong Road, Chao Yang District, Beijing 100029, China; ^3^Fangshan Hospital, Beijing University of Chinese Medicine, Beijing 102400, China

## Abstract

Traditional Chinese medicine (TCM) has a longstanding history and has gained widespread clinical applications. San Cao Decoction (SCD) is an experience prescription first formulated by Prof. Duzhou Liu. We previously demonstrated its antihypertensive effects; however, to systematically explain the underlying mechanisms of action, we employed a systems pharmacology approach for pharmacokinetic screening and target prediction by constructing protein-protein interaction networks of hypertension-related and putative SCD-related targets, and Database for Annotation, Visualization, and Integrated Discovery enrichment analysis. We identified 123 active compounds in SCD and 116 hypertension-related targets. Furthermore, the enrichment analysis of the drug-target network showed that SCD acts in a multidimensional manner to regulate PI3K-Akt-endothelial nitric oxide synthase signaling to maintain blood pressure. Our results highlighted the molecular mechanisms of antihypertensive actions of medicinal herbs at a systematic level.

## 1. Introduction

Hypertension is a chronic condition that triggers various fatal cardiovascular disorders, including heart failure, coronary artery disease, stroke, peripheral artery disease, and renal failure [1]. It was responsible for causing almost 10 million deaths in 2013 worldwide [2]. A nationwide survey in China during 2013–2014 showed that 27.8% (292 million) of the adult population was hypertensive, indicating that the number increased since 2002 [3]. Therefore, lowering the blood pressure is critical to prevent organ damage and cardiovascular mortality in hypertensive patients [4].

The WHO global atlas of traditional, complementary, and alternative medicine reports a global upsurge in herbal and traditional medicinal practices [5]. Although traditional remedies for hypertension have existed since a thousand years in China, their molecular mechanisms of action remain elusive. Recently, the efficacy of some Chinese herbal formulations has been demonstrated through case studies and clinical trials [6–8]. San Cao Decoction (SCD) is an experience prescription that was first formulated by Prof. Duzhou Liu, based on the Shao Yao Gan Cao Decoction, which was described in the Shang Han Lun and compiled by Zhang Zhongjing. This formulation contains five components, namely fruit-spike of* Prunella vulgaris* L (spica prunellae); root of* Glycyrrhiza uralensis* Fisch,* Glycyrrhiza inflata* Bat, or* Glycyrrhiza glabra* L (radix glycyrrhizae); root of* Paeonia lactiflora* Pall (radix paeoniae alba); aerial parts of* Leonurus japonicus* Houtt (herba leonuri); and root of* Gentiana lutea* L (radix gentianae). We previously demonstrated that SCD exhibits antihypertensive effects by modulating nitric oxide (NO)/endothelin-1 signaling and increasing NO level as well as by protecting against H_2_O_2_-induced injury through the regulation of the endothelial nitric oxide synthase (eNOS)/NO pathway in human umbilical vein endothelial cells [9–13]. However, the identification of the antihypertensive mechanisms of individual components of SCD needs further investigation.

Because herbal formulations contain multiple components exhibiting multidimensional pharmacological effects, it is a challenge to identify the effects of individual components using traditional methods of analyses. Moreover, the pharmacological effects of herbal formulations depend on the complex and dynamic interactions among its components [14]. Using systems pharmacology approach, it is capable of identifying compound-compound, compound-target, and target-disease interactions in a network model and understanding the effect of herbs on biological networks based on a systemic theory [15].

In the present study, a systems pharmacology approach was employed to screen the oral bioavailability (OB) and drug-likeness (DL) of the individual compounds of SCD. The potential biological targets of these active ingredients and interaction networks were obtained from public databases, which has been previously used to study the biological mechanisms of Niao Du Qing granules [16] and Tianfoshen oral liquid [17]. Protein-protein interaction (PPI) networks of hypertension-related and putative SCD-related targets were constructed, and core targets were identified through topological screening. Finally, we performed an enrichment analysis using the Database for Annotation, Visualization, and Integrated Discovery (DAVID), and deciphered the mechanistic pathways underlying the antihypertensive effects of SCD.

## 2. Materials and Methods

### 2.1. Data Mining

Data on the individual herbs in SCD were mined from the TCM systems pharmacology (TCMSP) database [18], one of the largest noncommercial database that includes data on drugs used in TCM from a systems pharmacology perspective. It is constructed based on scientific publications and medical literature on TCM and includes data on more than 13,731 pure compounds isolated from 505 TCM herbs. The structures of the individual SCD compounds were sourced from the NCBI PubChem database and by large-scale biomedical literature mining.

### 2.2. Prediction of Active Compounds in SCD

Although formulations in TCM are composed of multiple compounds, all of them may not be pharmacologically active. Therefore, the identification of the pharmacokinetic properties of each compound in TCM is essential. We screened various compounds present in SCD based on their pharmacokinetic absorption, distribution, metabolism, and excretion (ADME) parameters, such as OB (systemic bioavailability after oral absorption and distribution) [19] and DL (structural similarity between compounds and clinically used drugs in the DrugBank database) [20], according to previously reported models [21]. A bioactive molecule with a high OB displays good DL, which is a qualitative concept utilized in drug design to optimize pharmaceutical and pharmacokinetic properties of molecules, such as chemical stability and solubility. Active compounds were selected based on threshold values of OB ≥ 30% and DL ≥ 0.18 [22]. Detailed information of these active compounds is described in Table S1.

### 2.3. Prediction of Putative Targets of SCD

For the current study, we chose a public database interrogation strategy performed as previously described to predict the pharmacological targets of the individual compound in SCD [23]; gene names were extracted from the UniProt Knowledgebase. Relationship between active compounds and putative targets is described in Table S2.

### 2.4. Identification of Hypertension-Related Targets

Known hypertension-related targets were identified from the following six existing resources: (1) the DrugBank database; we identified interactions between FDA-approved drugs for hypertension treatment and human gene/protein targets [24]; (2) the Online Mendelian Inheritance in Man (OMIM) database using “hypertension” as the keyword [25]; (3) the Genetic Association Database (GAD) using “hypertension” as the keyword [26]; (4) the Kyoto Encyclopedia of Genes and Genomes (KEGG) Pathway, pathway database, Disease H number, H01633 [27]; (5) the Therapeutic Target Database (TTD) database [28]; and (6) Text-mined Hypertension, Obesity and Diabetes candidate gene database (T-HOD) using “hypertension” as the keyword [29]. Detailed information on these known therapeutic targets is described in Table S3.

### 2.5. PPI Network Construction

A PPI network was constructed using Bisogenet, a Cytoscape plugin, for the analysis of five existing PPI databases, including the Biological General Repository for Interaction Datasets, the Biomolecular Interaction Network Database, the Molecular INTeraction Database, the Human Protein Reference Database, and the Database of Interacting Proteins [30]. We constructed two PPI networks for further analysis, which contain compounds putative targets and known hypertenssive therapeutic targets, respectively.

### 2.6. Identification of Candidate SCD Targets Responsible for Its Antihypertensive Effects

We constructed an interaction network for the known hypertension-related targets and putative pharmacological targets of SCD based on data obtained using the Cytoscape plugin, Bisogenet. Further, the interaction network was visualized using the Cytoscape software (Version 3.2.1), and the topological properties of each node in the interaction network were assessed using another Cytoscape plugin (CytoNCA) on the basis of betweenness centrality (BC), degree centrality (DC), closeness centrality (CC), eigenvector centrality (EC), network centrality (NC), and local average connectivity (LAC). The definitions and computational formulas of these parameters have been previously described [31]. The values of these parameters directly correlate with the importance of the node in the network. Following twice topological screening, all targets in the core PPI network were considered as candidate targets.

### 2.7. GO and KEGG Pathway Enrichment Analysis

We analyzed 172 putative targets of SCD using GO enrichment with DAVID to identify their involvement based on three different terms including biological process (BP), cell component (CC), and molecular function (MF) terms. With p <0.05, we applied a hypergeometric test to identify enriched GO terms. An overview of the GO analysis with up to 10 significantly enriched terms in each of these three categories is shown. Further, we performed a DAVID-based enrichment analysis of 116 candidate targets of SCD with the KEGG signaling pathway; we only selected terms with p <0.05.

## 3. Results

### 3.1. Screening of the Active Compounds in SCD

**Figure 1:**
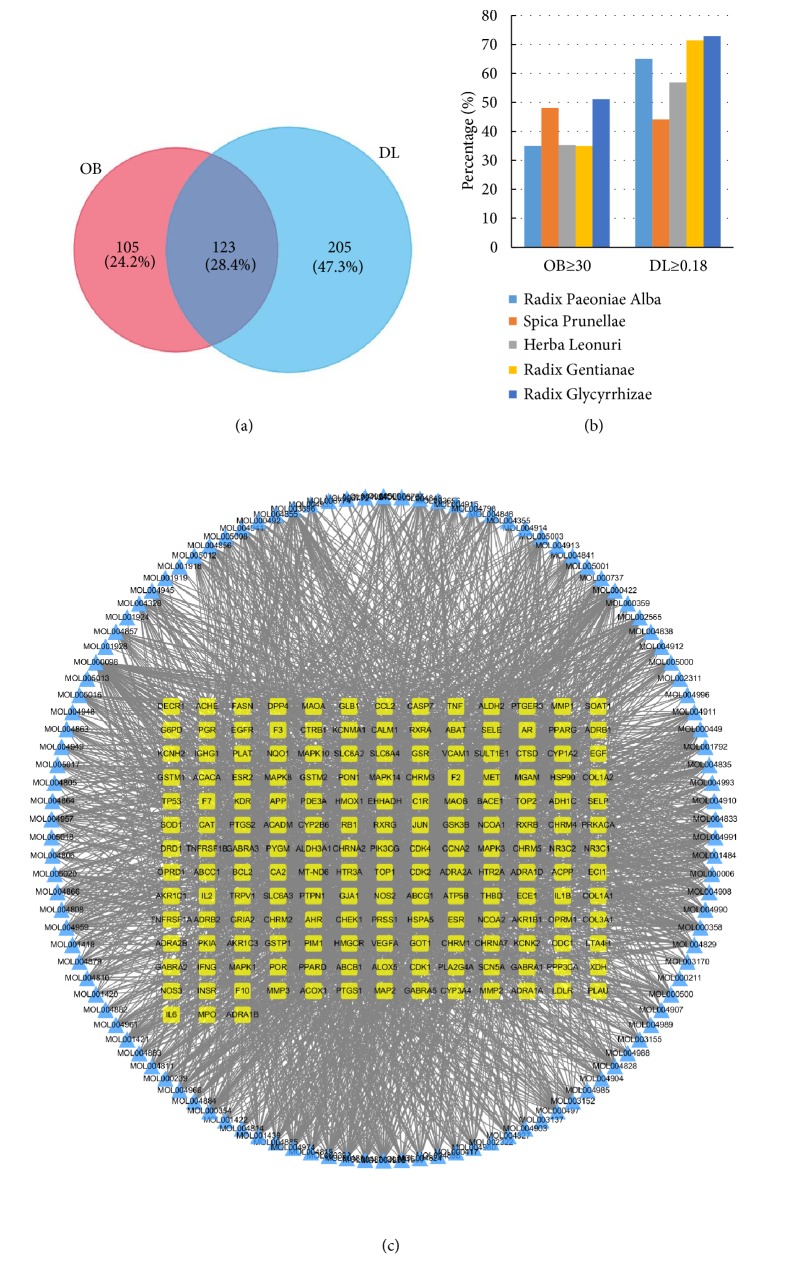
Analysis of the active compounds of SCD and preliminary GO analysis of putative SCD targets. (a) Active compounds in SCD were preliminarily screened for two ADME parameters. (b) ADME parameter distribution for different herbs. (c) The compound-target network plotting.

Based on previous reports, 60, 77, 51, 63, and 280 compounds for each of the five medicinal herbs,* spica prunellae*,* radix paeoniae alba*,* herba leonuri*,* radix gentianae*, and* radix glycyrrhizae*, respectively, were included in this study. These active compounds were screened for ADME-related pharmacokinetic parameters, OB and DL [32]; the screening criteria were OB ≥ 30% and DL ≥ 0.18 [33]. Based on these criteria, 45.4% (228/502) and 65.3% (328/502) of the compounds had favorable OB and DL, respectively. After screening and excluding duplicates, 123 potential compounds were included (Figures 1(a) and 1(b));* radix paeoniae alba*,* spica prunellae*,* herba leonuri*,* radix gentianae,* and* radix glycyrrhizae* contained 11, 13, 8, 10, and 92 compounds, respectively. The detailed ADME parameters and the structural information of these compounds are shown in Table S1. Among the 123 candidate compounds, six compounds were abundant in multiple herbs and reported to exhibit diverse biological effects. For example, kaempferol, present in all five herbs, is reported to treat vascular disorders via antiatherosclerotic, antioxidant and anti-inflammatory activities [34, 35].

### 3.2. Generating a Compound-Target Network for SCD

**Figure 2:**
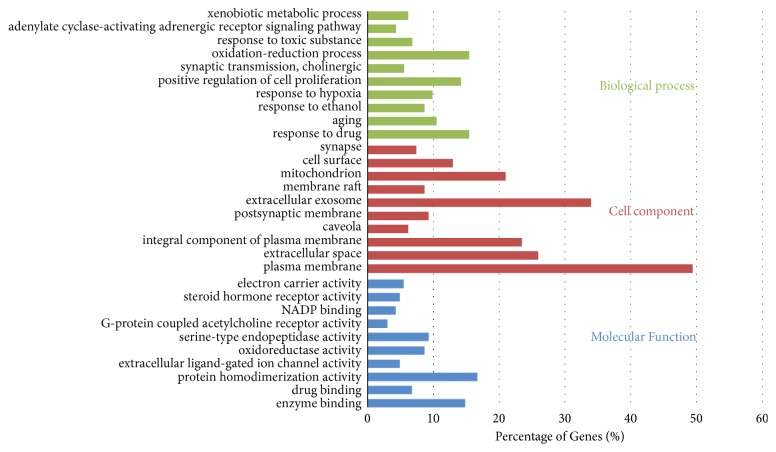
GO analysis for targets of SCD. Biological processes, cell component, and molecular function terms were performed on putative SCD targets; the top 10 terms with P < 0.05 are shown. Terms in the same category are ordered by p values (95% confidence level) starting with the most significant values on top. The percentage of genes/proteins involved in a term is presented at the bottom of the figure.

We identified 172 putative therapeutic targets for 112 of the 123 candidate compounds of SCD by integrating available chemical, genomic, and pharmacological information [36]; however, targets for 11 compounds could not be identified. Despite the varied number of targets for compounds belonging to each herb, they significantly overlapped, indicating that they acted synergistically. To further understand the complex interactions between the compounds and their corresponding targets at a system level, we constructed a compound-target network based on the candidate compounds of SCD and their potential targets (Figure 1(c)). The network contained 286 nodes and 2450 edges. In a network topology analysis, the degree of a vertex node represents the number of edges incident to it. We identified 63 candidate compounds having a median of 21 degrees, suggesting that most of the compounds acted on multiple targets: quercetin, kaempferol, and beta-sitosterol interacted with 85, 54, and 49 targets, respectively. This could explain the pleiotropic effects exhibited by the active compounds in SCD. DAVID-based gene ontology (GO) enrichment analysis of putative SCD targets were performed using BP, CC and MF terms. We identified 401 BPs, 73 CCs, and 114 MFs that were enriched in this dataset with p <0.05.  Figure 2 illustrates an overview of the GO analysis with 10 remarkably enriched BP, CC, and MF terms. The results showed that these targets participate in signal transduction mechanisms to elicit responses to drugs along with other cellular metabolic processes.

### 3.3. Identification of Hypertension-Related Targets

**Figure 3:**
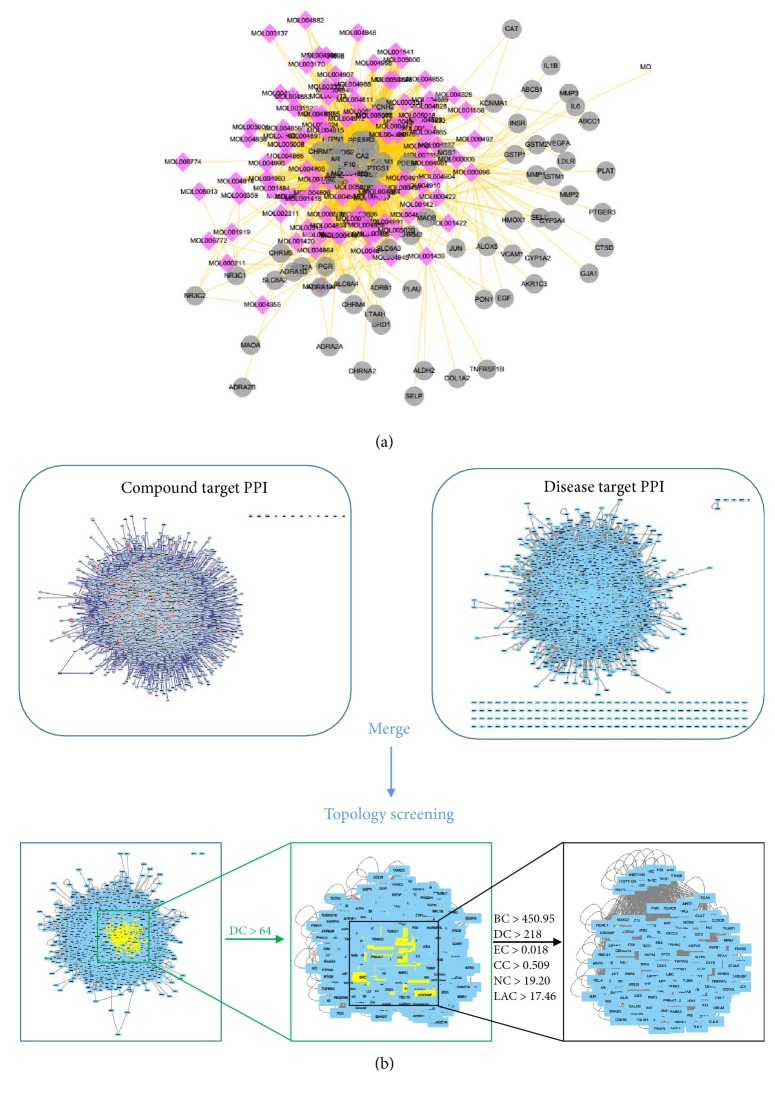
Identification of candidate targets for SCD against hypertension. (a) SCD shared 75 putative targets with known antihypertension drugs. The compound-putative target network was constructed by linking the overlapped targets (between SCD putative and known hypertension-related) and the homologous candidate compounds of SCD. The nodes representing candidate compounds are shown as polychrome rhombus and the targets are presented as grey circles. (b) Identification of candidate SCD targets for hypertension treatment through PPI network. 116 candidate targets are finally predicted.

The pharmacological target of a drug determines its indication. We identified hypertension-related targets using various databases, including DrugBank, OMIM, GAD, KEGG Pathway, TTD, and T-HOD database. After compensating for data redundancy, 913 hypertension-related targets were identified (Table S3). Of these, 75 were targets of the herbs comprising SCD, which verifies its therapeutic potential as an antihypertensive TCM formulation. We constructed and analyzed the compound antihypertension targets network (Figure 3(a), Table S4). Based on degree centrality, top six active compounds in SCD were quercetin, kaempferol, 8-prenylwighteone, isorhamnetin, 7-methoxy-2-methyl isoflavone, and medicarpin, of which degree was 41, 31, 28, 28, 26, and 24, respectively.

### 3.4. Identification of Candidate SCD Targets Responsible for Its Antihypertensive Effects

Genes and proteins do not function independently; instead they work on multiple levels via interconnected molecular networks and pathways [37]. PPI networks reflect the behavior and properties of biological molecules; they are especially useful to understand the role of various proteins in complex diseases, such as hypertension. Therefore, we selected proteins as nodes and constructed a PPI network to identify pharmacological mechanisms by which SCD ameliorates hypertension [38].

First, a putative target PPI network of SCD-related genes was constructed with 6,634 nodes and 1,50,547 edges on a systems pharmacology platform. After further extraction of hypertension-related targets, a disease-specific network was constructed with 7,509 nodes and 1,65,579 edges. We then merged these two networks to obtain a core PPI network with 4,563 nodes and 1,21,159 edges. Subsequently, candidate hypertension-related proteins modified by SCD were screened based on the topological features of the core PPI. A node is identified as a significant target if its degree was more than twice the median degree of all nodes in the network [39]; the median degree in our network was 32. Thus, we constructed a network of significant targets of SCD with 1,120 nodes and 50,778 edges. Using a Cytoscape plugin (CytoNCA), we selected the following seven topological features to identify candidate targets: BC, DC, CC, EC, NC, and LAC [31]. The median values of BC, DC, EC, CC, NC, and LAC were 450.9586438, 218, 0.018675338, 0.509099181, 19.20085046, and 17.46794872, respectively. Thus, we identified 116 candidate targets with values for these topological features higher than the reported median values. A representative flow chart of the screening process is presented in Figure 3(b). Detailed topological features of the core PPI network and the 116 candidate targets are shown in Table S5.

### 3.5. Pathway Enrichment Analysis for Candidate SCD Targets

**Figure 4:**
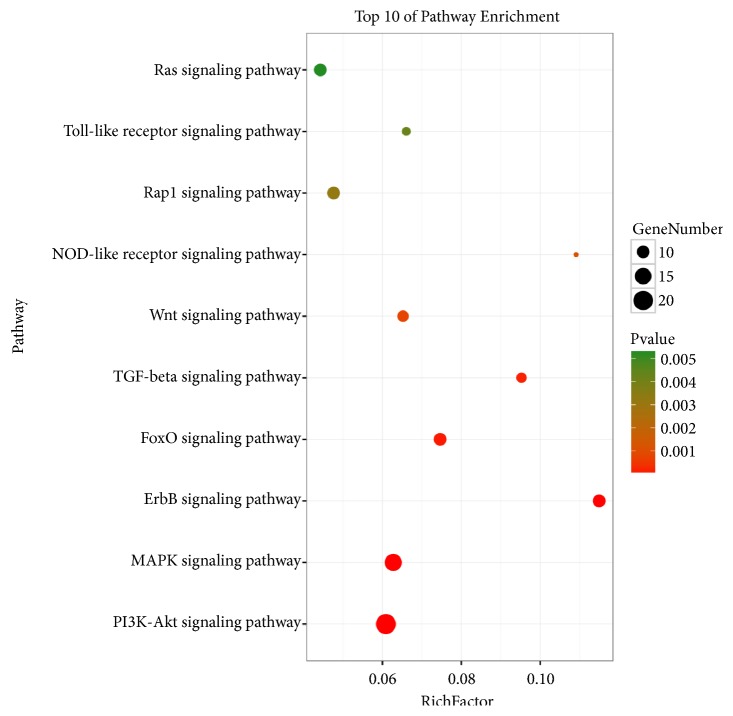
Enrichment analysis of candidate targets for SCD against hypertension.

**Figure 5:**
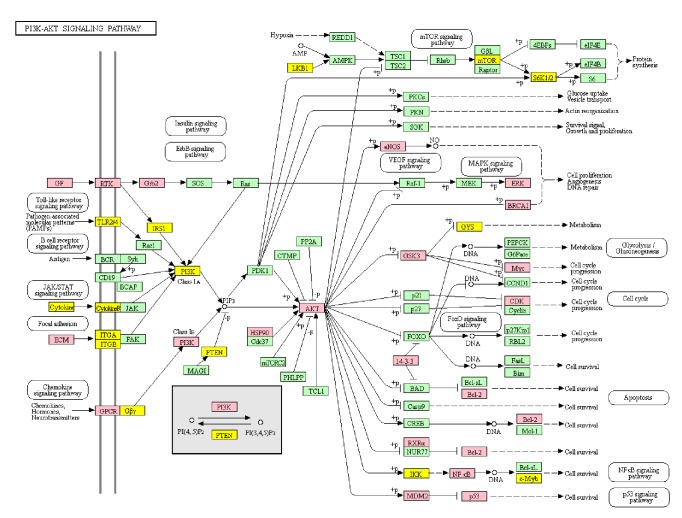
Modulating PI3K-Akt signaling pathway of SCD. Targets of SCD were colored in pink, targets of hypertension were colored in yellow, and proteins in the pathway were colored in green.

Based on DAVID enrichment, we correlated BP and MF terms with proteins involved in cellular processes, such as the regulation of NOS expression, cell adhesion mediated by cadherin binding, and the regulation of apoptosis by p53 signaling. Various signaling molecules related hypertension, such as PI3K-Akt, MAPK, ErbB, FoxO, TGF-beta, Wnt, NOD-like receptor, Rap1, Toll-like receptor, and Ras signaling pathways, were identified (ordered in P-value, Figure 4). We choose PI3K-Akt signaling pathway to further understand the antihypertensive effect of SCD. A concept map contains SCD targets (pink) and hypertension targets (yellow) in PI3K/Akt signaling pathway (Figure 5). Based on these data, we postulated that SCD inhibits hypertension by regulating PI3K-Akt-eNOS signaling.

## 4. Discussion

Long-established Chinese herbal formulations not only stabilize the blood pressure, but also improve the quality of life, minimize hypertension-related risk factors, and prevent organ damage to improve patient survival [40]. Although the antihypertensive effects of SCD have been recognized, underlying molecular mechanisms are not well established. In the present study, a systems pharmacology method was applied to identify bioactive compounds in SCD and the pathways modulated by these compounds by evaluating their OB and DL. We used the TCM systems pharmacology database to retrieve 501 candidate compounds present in SCD. After screening, 123 compounds were identified to possess favorable OB and DL properties. Quercetin, kaempferol, 8-Prenylwighteone, isorhamnetin, 7-Methoxy-2-methyl isoflavone, and medicarpin were considered as potential antihypertension compounds in SCD. Evidence showed that some compounds indentifieed in the present approach have some cardiovascular pharmacological activities. Previous studies suggested quercetin was an orally active bioflavonoid that has been reported to play critical roles in treating cardiovascular diseases [41]. Quercetin was also considered to reduce blood pressure via upregulating eNOS/NO signal. Kaempferol was also reported to inhibit activity of ACE and decrease blood pressure [42, 43].

To understand potential biological mechanism of SCD, a PPI network was constructed, and 116 potential targets were recognized. Through the KEGG pathway analysis, we recognized ten hypertension-related signaling pathways, PI3K-Akt, MAPK, ErbB, FoxO, TGF-beta, Wnt, NOD-like receptor, Rap1, Toll-like receptor, and Ras signaling pathways. Actually, these pathways may be involved in the progress of hypertension. Based on P-Value, we choose PI3K-Akt signal pathway as most candidate signal for further study. We constructed a concept map containing SCD targets and hypertension targets in PI3K/Akt signaling pathway and found a synergistic effect of SCD targets in this pathway to treat hypertension. We highlighted PI3K-Akt-eNOS as an important signal pathway based on its role in regulating blood pressure [44] and our previous experimental data.

We previously demonstrated that SCD increases eNOS activation to increase NO levels* in vivo* and* in vitro* [11, 13]. The phosphorylation of eNOS is induced by Akt, the downstream effector of PI3K [45]; we identified that SCD exerts its antihypertensive effect by regulating this signaling pathway. Activation of eNOS promotes NO production by the endothelium to maintain vascular tone [46]. NO is an endogenous antagonist of angiotensin II and endothelin-1 [47]. In addition to regulating vascular tone, NO attenuates inflammation, atherosclerosis, and apoptosis [48, 49]. Moreover, hypertension is characterized by the deficiency of eNOS and NO, especially in the endothelium [50]. Therefore, SCD may reduce blood pressure by regulating PI3K-Akt-eNOS signaling.

## 5. Conclusions

In this study, we predicted the mechanisms underlying the antihypertensive effects of SCD to verify its potential to treat hypertension. We recognized that SCD acts by modulating the PI3K-Akt-eNOS pathway to produce antihypertensive effects. This study demonstrates the usefulness of a systems pharmacology-based approach to elucidate relationships between complex diseases, such as hypertension, and Chinese herbal medicines. A limitation of our study is that the target-prediction tools used in our systems pharmacology analysis only reveal indeterminate connections between compounds and their corresponding target genes. Therefore, further experimental studies are required to accurately determine and validate the predicted mechanisms of action.

## Data Availability

The data used to support the findings of this study are available from the corresponding author upon request.

## Supplementary Materials

Supplementary materials Supplementary materials contain five tables. Table S1: detailed information of active compounds in SCD; Table S2: relationship between active compounds and putative targets; Table S3: detailed information on these known therapeutic targets; Table S4: topological features of antihypertension targets related active compounds in SCD; Table S5: topological features of 116 candidate targets.
