# Supplementary material for: A Systems Pharmacology-Based Study of the Molecular Mechanisms of San Cao Decoction for Treating Hypertension
Source: Evid Based Complement Alternat Med. 2019 Jul 2;2019:3171420. doi: 10.1155/2019/3171420 (PMC6632497; doi:10.1155/2019/3171420)
Supplement: Supplementary materials — contain five tables. Table S1: detailed information of active compounds in SCD; Table S2: relationship between active compounds and putative targets; Table S3: detailed information on these known therapeutic targets; Table S4: topological features of antihypertension targets related active compounds in SCD; Table S5: topological features of 116 candidate targets. [file 3171420.f1.docx]

Table S1 Detailed information of active compounds in SCD

| MOL_ID | molecule_name | OB | MW | Drug-likeness | SMILES |
| --- | --- | --- | --- | --- | --- |
| MOL000006 | luteolin | 36.16263 | 286.25 | 0.24552 | C1=CC(=C(C=C1C2=CC(=O)C3=C(C=C(C=C3O2)O)O)O)O |
| MOL000098 | quercetin | 46.43335 | 302.25 | 0.27525 | C1=CC(=C(C=C1C2=C(C(=O)C3=C(C=C(C=C3O2)O)O)O)O)O |
| MOL000358 | beta-sitosterol | 36.91391 | 414.79 | 0.75123 | CCC(CCC(C)C1CCC2C1(CCC3C2CC=C4C3(CCC(C4)O)C)C)C(C)C |
| MOL000422 | kaempferol | 41.88225 | 286.25 | 0.24066 | C1=CC(=CC=C1C2=C(C(=O)C3=C(C=C(C=C3O2)O)O)O)O |
| MOL000449 | Stigmasterol | 43.82985 | 412.77 | 0.75665 | CCC(C=CC(C)C1CCC2C1(CCC3C2CC=C4C3(CCC(C4)O)C)C)C(C)C |
| MOL000737 | morin | 46.22959 | 302.25 | 0.27457 | C1=CC(=C(C=C1O)O)C2=C(C(=O)C3=C(C=C(C=C3O2)O)O)O |
| MOL004355 | Spinasterol | 42.97937 | 412.77 | 0.75534 | CCC(C=CC(C)C1CCC2C1(CCC3C2=CCC4C3(CCC(C4)O)C)C)C(C)C |
| MOL004798 | delphinidin | 40.63498 | 303.26 | 0.27763 | CCC(C=CC(C)C1CCC2C1(CCC3C2=CCC4C3(CCC(C4)O)C)C)C(C)C |
| MOL006767 | Vulgaxanthin-I | 56.13969 | 339.34 | 0.25836 | C1C(NC(=CC1=CC=NC(CCC(=O)N)C(=O)O)C(=O)O)C(=O)O |
| MOL006772 | poriferasterol monoglucoside_qt | 43.82985 | 412.77 | 0.75769 | CCC(C=CC(C)C1CCC2C1(CCC3C2CC=C4C3(CCC(C4)OC5C(C(C(C(O5)CO)O)O)O)C)C)C(C)C |
| MOL006774 | stigmast-7-enol | 37.42312 | 414.79 | 0.75133 | CCC(CCC(C)C1CCC2C1(CCC3C2=CCC4C3(CCC(C4)O)C)C)C(C)C |
| MOL000354 | isorhamnetin | 49.60438 | 316.28 | 0.306 | COC1=C(C=CC(=C1)C2=C(C(=O)C3=C(C=C(C=C3O2)O)O)O)O |
| MOL001418 | galeopsin | 61.01548 | 376.54 | 0.3753 | CC(=O)OC1(C(=O)CC2C(CCCC2(C1(CCC3=COC=C3)O)C)(C)C)C |
| MOL001420 | ZINC04073977 | 37.99619 | 412.77 | 0.75755 | CCC(CCC(C)C1CCC2C1(CCC3C2CC=C4C3(CCC(=O)C4)C)C)C(C)C |
| MOL001421 | preleoheterin | 85.97259 | 334.5 | 0.33044 | CC1C(C(=O)C2C(CCCC2(C13CCC4(O3)COC=C4)C)(C)C)O |
| MOL001422 | iso-preleoheterin | 66.28878 | 334.5 | 0.33032 | CC1C(C(=O)C2C(CCCC2(C13CCC4(O3)COC=C4)C)(C)C)O |
| MOL001439 | arachidonic acid | 45.57325 | 304.52 | 0.20409 | CCCCCC=CCC=CCC=CCC=CCCCC(=O)O |
| MOL000359 | sitosterol | 36.91391 | 414.79 | 0.7512 | CCC(CCC(C)C1CCC2C1(CCC3C2CC=C4C3(CCC(C4)O)C)C)C(C)C |
| MOL001558 | sesamin | 56.54706 | 354.38 | 0.82722 | C1C2C(COC2C3=CC4=C(C=C3)OCO4)C(O1)C5=CC6=C(C=C5)OCO6 |
| MOL002322 | isovitexin | 31.29464 | 432.41 | 0.71838 | C1=CC(=CC=C1C2=CC(=O)C3=C(C(=C(C=C3O2)O)C4C(C(C(C(O4)CO)O)O)O)O)O |
| MOL003137 | Leucanthoside | 32.11589 | 462.44 | 0.78146 | COC1=C(C(=C2C(=C1)OC(=CC2=O)C3=CC(=C(C=C3)O)O)O)C4C(C(C(C(O4)CO)O)O)O |
| MOL003143 | gentirigenic acid | 38.77662 | 522.8 | 0.77981 | CC1(C(C(CC(O1)(C2CCC3(C2CCC4C3(CCC5C4(CCC(C5(C)CO)O)C)C)C)C(=O)O)O)O)C |
| MOL003152 | Gentisin | 64.06193 | 258.24 | 0.21335 | COC1=CC(=C2C(=C1)OC3=C(C2=O)C=C(C=C3)O)O |
| MOL003155 | pranferin | 52.14348 | 318.4 | 0.28408 | CC1(C(OC(O1)(C)C)C2=C(C=CC3=C2OC(=O)C=C3)OC)C |
| MOL003169 | Gentiopicroside tetraacetate | 32.43546 | 524.52 | 0.74805 | CC(=O)OCC1C(C(C(C(O1)OC2C(C3=CCOC(=O)C3=CO2)C=C)OC(=O)C)OC(=O)C)OC(=O)C |
| MOL003170 | Gentisein | 67.5725 | 244.21 | 0.18962 | C1=CC2=C(C=C1O)C(=O)C3=C(C=C(C=C3O2)O)O |
| MOL000211 | Mairin | 55.37707 | 456.78 | 0.7761 | CC(=C)C1CCC2(C1C3CCC4C5(CCC(C(C5CCC4(C3(CC2)C)C)(C)C)O)C)C(=O)O |
| MOL000492 | (+)-catechin | 54.82643 | 290.29 | 0.24164 | C1C(C(OC2=CC(=CC(=C21)O)O)C3=CC(=C(C=C3)O)O)O |
| MOL001910 | 11alpha,12alpha-epoxy-3beta-23-dihydroxy-30-norolean-20-en-28,12beta-olide | 64.77389 | 470.71 | 0.37586 | CC(=CCC1=CC=CC2=C1OC(CC2=O)C3=CC=C(C=C3)O)C |
| MOL001918 | paeoniflorgenone | 87.59312 | 318.35 | 0.36678 | CC12CC(=O)C3CC1(OC(C3COC(=O)C4=CC=CC=C4)O2)O |
| MOL001919 | (3S,5R,8R,9R,10S,14S)-3,17-dihydroxy-4,4,8,10,14-pentamethyl-2,3,5,6,7,9-hexahydro-1H-cyclopenta[a]phenanthrene-15,16-dione | 43.5562 | 358.52 | 0.53276 | CC1(C2CCC3(C(C2(CCC1O)C)CC(C4=CCCC43C)O)C)C |
| MOL001921 | Lactiflorin | 49.12132 | 462.49 | 0.79711 | CC12C3(CC4C3(C(O1)CC4=O)COC(=O)C5=CC=CC=C5)OC6C(O2)C(C(C(O6)CO)O)O |
| MOL001924 | paeoniflorin | 53.87038 | 480.51 | 0.78709 | CC12CC3(C4CC1(C4(C(O2)O3)COC(=O)C5=CC=CC=C5)OC6C(C(C(C(O6)CO)O)O)O)O |
| MOL001925 | paeoniflorin_qt | 68.17576 | 318.35 | 0.39507 | CC12CC3(C4CC1(C4(C(O2)O3)COC(=O)C5=CC=CC=C5)OC6C(C(C(C(O6)CO)O)O)O)O |
| MOL001928 | albiflorin_qt | 66.64077 | 318.35 | 0.32626 | CC12CC(C3CC1(C3(C(=O)O2)COC(=O)C4=CC=CC=C4)OC5C(C(C(C(O5)CO)O)O)O)O |
| MOL001930 | benzoyl paeoniflorin | 31.27447 | 584.62 | 0.74612 | CC12CC3(C4CC1(C4(C(O2)O3)COC(=O)C5=CC=CC=C5)OC6C(C(C(C(O6)COC(=O)C7=CC=CC=C7)O)O)O)O |
| MOL000239 | Jaranol | 50.82882 | 314.31 | 0.29148 | COC1=CC(=C2C(=C1)OC(=C(C2=O)OC)C3=CC=C(C=C3)O)O |
| MOL000392 | formononetin | 69.67388 | 268.28 | 0.21202 | COC1=CC=C(C=C1)C2=COC3=C(C2=O)C=CC(=C3)O |
| MOL000417 | Calycosin | 47.75183 | 284.28 | 0.24278 | COC1=C(C=C(C=C1)C2=COC3=C(C2=O)C=CC(=C3)O)O |
| MOL000497 | licochalcone a | 40.78965 | 338.43 | 0.28517 | CC(C)(C=C)C1=C(C=C(C(=C1)C=CC(=O)C2=CC=C(C=C2)O)OC)O |
| MOL000500 | Vestitol | 74.65519 | 272.32 | 0.20935 | COC1=CC(=C(C=C1)C2CC3=C(C=C(C=C3)O)OC2)O |
| MOL001484 | Inermine | 75.18306 | 284.28 | 0.53754 | C1C2C(C3=C(O1)C=C(C=C3)O)OC4=CC5=C(C=C24)OCO5 |
| MOL001792 | DFV | 32.76272 | 256.27 | 0.18316 | C1C(OC2=C(C1=O)C=CC(=C2)O)C3=CC=C(C=C3)O |
| MOL002311 | Glycyrol | 90.77578 | 366.39 | 0.66819 | CC(=CCC1=C(C=C2C(=C1OC)C3=C(C4=C(O3)C=C(C=C4)O)C(=O)O2)O)C |
| MOL002565 | Medicarpin | 49.21982 | 270.3 | 0.3351 | COC1=CC2=C(C=C1)C3COC4=C(C3O2)C=CC(=C4)O |
| MOL003656 | Lupiwighteone | 51.63569 | 338.38 | 0.36739 | CC(=CCC1=C(C=C(C2=C1OC=C(C2=O)C3=CC=C(C=C3)O)O)O)C |
| MOL003896 | 7-Methoxy-2-methyl isoflavone | 42.56474 | 266.31 | 0.19946 | CC(=C(CC1=C(C=CC(=C1)C2=COC3=CC(=CC(=C3C2=O)O)OC)O)O)C |
| MOL004328 | naringenin | 59.2939 | 272.27 | 0.21128 | C1C(OC2=CC(=CC(=C2C1=O)O)O)C3=CC=C(C=C3)O |
| MOL004805 | (2S)-2-[4-hydroxy-3-(3-methylbut-2-enyl)phenyl]-8,8-dimethyl-2,3-dihydropyrano[2,3-f]chromen-4-one | 31.78703 | 390.51 | 0.72403 | CC(=CCC1=C(C=CC(=C1)C2CC(=O)C3=C(O2)C4=C(C=C3)OC(C=C4)(C)C)O)C |
| MOL004806 | euchrenone | 30.28726 | 406.56 | 0.57386 | CC(=CCC1=C(C=CC2=C1OC(CC2=O)C3=C(C=C(C=C3)O)O)O)C |
| MOL004808 | glyasperin B | 65.22439 | 370.43 | 0.43851 | CC(=CCC1=C(C=C2C(=C1O)C(=O)C(CO2)C3=C(C=C(C=C3)O)O)OC)C |
| MOL004810 | glyasperin F | 75.8368 | 354.38 | 0.53514 | CC1(C=CC2=C(C=CC(=C2O1)C3COC4=CC(=CC(=C4C3=O)O)O)O)C |
| MOL004811 | Glyasperin C | 45.56381 | 356.45 | 0.39947 | CC(=CCC1=C(C=C2C(=C1OC)CC(CO2)C3=C(C=C(C=C3)O)O)O)C |
| MOL004814 | Isotrifoliol | 31.94479 | 298.26 | 0.42422 | COC1=C2C(=CC(=C1)O)OC(=O)C3=C2OC4=C3C=CC(=C4)O |
| MOL004815 | (E)-1-(2,4-dihydroxyphenyl)-3-(2,2-dimethylchromen-6-yl)prop-2-en-1-one | 39.61686 | 322.38 | 0.35077 | CC1(C=CC2=C(O1)C=CC(=C2)C=CC(=O)C3=C(C=C(C=C3)O)O)C |
| MOL004820 | kanzonols W | 50.48008 | 336.36 | 0.51704 | CC1(C=CC2=C(O1)C=CC3=C2OC(=O)C(=C3)C4=C(C=C(C=C4)O)O)C |
| MOL004824 | (2S)-6-(2,4-dihydroxyphenyl)-2-(2-hydroxypropan-2-yl)-4-methoxy-2,3-dihydrofuro[3,2-g]chromen-7-one | 60.25041 | 384.41 | 0.63433 | CC(C)(C1CC2=C(O1)C=C3C(=C2OC)C=C(C(=O)O3)C4=C(C=C(C=C4)O)O)O |
| MOL004827 | Semilicoisoflavone B | 48.77755 | 352.36 | 0.54732 | CC1(C=CC2=C(O1)C(=CC(=C2)C3=COC4=CC(=CC(=C4C3=O)O)O)O)C |
| MOL004828 | Glepidotin A | 44.72187 | 338.38 | 0.34685 | CC(=CCC1=C(C=C(C2=C1OC(=C(C2=O)O)C3=CC=CC=C3)O)O)C |
| MOL004829 | Glepidotin B | 64.46292 | 340.4 | 0.34485 | CC(=CCC1=C(C=C(C2=C1OC(C(C2=O)O)C3=CC=CC=C3)O)O)C |
| MOL004833 | Phaseolinisoflavan | 32.00811 | 324.4 | 0.44538 | CC1(C=CC2=C(O1)C=CC(=C2O)C3CC4=C(C=C(C=C4)O)OC3)C |
| MOL004835 | Glypallichalcone | 61.59706 | 284.33 | 0.18993 | COC1=CC=C(C=C1)C(=O)C=CC2=C(C=C(C=C2)O)OC |
| MOL004838 | 8-(6-hydroxy-2-benzofuranyl)-2,2-dimethyl-5-chromenol | 58.43728 | 308.35 | 0.38106 | CCC1=C(C(=C(C(=O)N1)CC2=C(C3=C(C(=C2O)C(=O)C(C)CC)OC(C=C3)(C)C)O)O)C |
| MOL004841 | Licochalcone B | 76.75735 | 286.3 | 0.1935 | COC1=C(C=CC(=C1O)O)C=CC(=O)C2=CC=C(C=C2)O |
| MOL004848 | licochalcone G | 49.25496 | 354.43 | 0.32325 | CC(C)(C=C)C1=C(C=C(C(=C1)C=CC(=O)C2=C(C=C(C=C2)O)O)OC)O |
| MOL004849 | 3-(2,4-dihydroxyphenyl)-8-(1,1-dimethylprop-2-enyl)-7-hydroxy-5-methoxy-coumarin | 59.62247 | 368.41 | 0.42894 | CC(C)(C=C)C1=C(C=C2C(=C1O)C(=O)CC(O2)C3=CC=CC=C3)OC |
| MOL004855 | Licoricone | 63.57846 | 382.44 | 0.4712 | CC(=CCC1=C(C=C(C(=C1OC)C2=COC3=C(C2=O)C=CC(=C3)O)O)OC)C |
| MOL004856 | Gancaonin A | 51.07519 | 352.41 | 0.40378 | CC(=CCC1=C(C=C2C(=C1O)C(=O)C(=CO2)C3=CC=C(C=C3)OC)O)C |
| MOL004857 | Gancaonin B | 48.7944 | 368.41 | 0.44924 | CC(=CCC1=C(C=C2C(=C1O)C(=O)C(=CO2)C3=CC(=C(C=C3)OC)O)O)C |
| MOL004860 | licorice glycoside E | 32.88743 | 693.71 | 0.27218 | C1C(OC2=C(C1=O)C=CC(=C2)O)C3=CC=C(C=C3)OC4C(C(C(C(O4)CO)O)O)OC5C(C(CO5)(COC(=O)C6=CNC7=CC=CC=C76)O)O |
| MOL004863 | 3-(3,4-dihydroxyphenyl)-5,7-dihydroxy-8-(3-methylbut-2-enyl)chromone | 66.37125 | 354.38 | 0.41392 | CC1=CC(=O)C2=C(C(=C(C=C2O1)O)CC=C(C)C)O |
| MOL004864 | 5,7-dihydroxy-3-(4-methoxyphenyl)-8-(3-methylbut-2-enyl)chromone | 30.48878 | 352.41 | 0.41002 | CC(=CCC1=C(C=C(C2=C1OC(CC2=O)C3=CC(=C(C=C3)O)OC)O)O)C |
| MOL004866 | 2-(3,4-dihydroxyphenyl)-5,7-dihydroxy-6-(3-methylbut-2-enyl)chromone | 44.15196 | 354.38 | 0.41482 | CC(=CCC1=C(C=CC(=C1O)C2COC3=CC(=C(C(=C3C2=O)O)CC=C(C)C)O)O)C |
| MOL004879 | Glycyrin | 52.60657 | 382.44 | 0.47466 | CC(=CCC1=C(C=C2C(=C1OC)C=C(C(=O)O2)C3=C(C=C(C=C3)O)O)OC)C |
| MOL004882 | Licocoumarone | 33.21085 | 340.4 | 0.3568 | CC(=CCC1=C(C=C2C(=C1OC)C=C(O2)C3=C(C=C(C=C3)O)O)O)C |
| MOL004883 | Licoisoflavone | 41.61022 | 354.38 | 0.41646 | CC(=CCC1=C(C=CC(=C1O)C2=COC3=CC(=CC(=C3C2=O)O)O)O)C |
| MOL004884 | Licoisoflavone B | 38.92871 | 352.36 | 0.54714 | CC1(C=CC2=C(O1)C=CC(=C2O)C3=COC4=CC(=CC(=C4C3=O)O)O)C |
| MOL004885 | licoisoflavanone | 52.46625 | 354.38 | 0.54488 | CC1(C=CC2=C(O1)C=CC(=C2O)C3COC4=CC(=CC(=C4C3=O)O)O)C |
| MOL004891 | shinpterocarpin | 80.29528 | 322.38 | 0.72746 | CC1(C=CC2=C(O1)C=CC3=C2OCC4C3OC5=C4C=CC(=C5)O)C |
| MOL004898 | (E)-3-[3,4-dihydroxy-5-(3-methylbut-2-enyl)phenyl]-1-(2,4-dihydroxyphenyl)prop-2-en-1-one | 46.26792 | 340.4 | 0.3062 | CC(=CCC1=C(C(=CC(=C1)C=CC(=O)C2=C(C=C(C=C2)O)O)O)O)C |
| MOL004903 | liquiritin | 65.69011 | 418.43 | 0.73893 | C1C(OC2=C(C1=O)C=CC(=C2)O)C3=CC=C(C=C3)OC4C(C(C(C(O4)CO)O)O)O |
| MOL004904 | licopyranocoumarin | 80.36001 | 384.41 | 0.6535 | CC1(CCC2=C(O1)C=C3C(=C2OC)C=C(C(=O)O3)C4=C(C=C(C=C4)O)O)CO |
| MOL004905 | 3,22-Dihydroxy-11-oxo-delta(12)-oleanene-27-alpha-methoxycarbonyl-29-oic acid | 34.31942 | 512.75 | 0.54718 | CC1(C2CCC3(C(C2(CCC1O)C)C(=O)C=C4C3(CCC5(C4CC6(CC5OC6=O)C)C)C(=O)OC)C)C |
| MOL004907 | Glyzaglabrin | 61.06889 | 298.26 | 0.35347 | C1OC2=C(O1)C(=C(C=C2)C3=COC4=C(C3=O)C=CC(=C4)O)O |
| MOL004908 | Glabridin | 53.24514 | 324.4 | 0.46967 | CC1(C=CC2=C(O1)C=CC3=C2OCC(C3)C4=C(C=C(C=C4)O)O)C |
| MOL004910 | Glabranin | 52.89566 | 324.4 | 0.31208 | CC(=CCC1=C(C=C(C2=C1OC(CC2=O)C3=CC=CC=C3)O)O)C |
| MOL004911 | Glabrene | 46.26686 | 322.38 | 0.43902 | CC1(C=CC2=C(C=CC(=C2O1)C3=CC4=C(C=C(C=C4)O)OC3)O)C |
| MOL004912 | Glabrone | 52.51217 | 336.36 | 0.49645 | CC1(C=CC2=C(O1)C=CC(=C2O)C3=COC4=C(C3=O)C=CC(=C4)O)C |
| MOL004913 | 1,3-dihydroxy-9-methoxy-6-benzofurano[3,2-c]chromenone | 48.14154 | 298.26 | 0.42831 | COC1=C(C=C2C(=C1)OC3=C2C(=O)OC4=C3C=CC(=C4)O)O |
| MOL004914 | 1,3-dihydroxy-8,9-dimethoxy-6-benzofurano[3,2-c]chromenone | 62.90135 | 328.29 | 0.52759 | CC1=C(C=C2C(=C1)C3=C(C4=CC(=C(C(=C4O3)C)O)O)C(=O)O2)C |
| MOL004915 | Eurycarpin A | 43.27728 | 338.38 | 0.37429 | CC(=CCC1=C(C=CC(=C1O)C2=COC3=C(C2=O)C=CC(=C3)O)O)C |
| MOL004917 | glycyroside | 37.25032 | 562.57 | 0.79156 | COC1=CC=C(C=C1)C2=COC3=C(C2=O)C=CC(=C3)OC4C(C(C(C(O4)CO)O)O)OC5C(C(CO5)(CO)O)O |
| MOL004924 | (-)-Medicocarpin | 40.99397 | 432.46 | 0.95059 | COC1=CC2=C(C=C1)C3COC4=C(C3O2)C=CC(=C4)OC5C(C(C(C(O5)CO)O)O)O |
| MOL004935 | Sigmoidin-B | 34.88109 | 356.4 | 0.41455 | CC(=CCC1=C(C(=CC(=C1)C2CC(=O)C3=C(C=C(C=C3O2)O)O)O)O)C |
| MOL004941 | (2R)-7-hydroxy-2-(4-hydroxyphenyl)chroman-4-one | 71.12299 | 256.27 | 0.18303 | CC1C(C(C(C(O1)OCC2C(C(C(C(O2)OC3=CC(=C4C(=O)CC(OC4=C3)C5=CC=C(C=C5)O)O)O)O)O)O)O)O |
| MOL004945 | (2S)-7-hydroxy-2-(4-hydroxyphenyl)-8-(3-methylbut-2-enyl)chroman-4-one | 36.56537 | 324.4 | 0.32291 | CC1(C=CC2=C(O1)C=CC3=C2OCC(C3)C4=C(C=C(C=C4)OC)O)C |
| MOL004948 | Isoglycyrol | 44.69923 | 366.39 | 0.83845 | CC1(CCC2=C(O1)C=C3C(=C2OC)C4=C(C5=C(O4)C=C(C=C5)O)C(=O)O3)C |
| MOL004949 | Isolicoflavonol | 45.16999 | 354.38 | 0.41859 | CC(=CCC1=C(C=CC(=C1)C2=C(C(=O)C3=C(C=C(C=C3O2)O)O)O)O)C |
| MOL004957 | HMO | 38.36542 | 268.28 | 0.21067 | COC1=CC2=C(C=C1)C(=O)C(=CO2)C3=CC=C(C=C3)O |
| MOL004959 | 1-Methoxyphaseollidin | 69.98098 | 354.43 | 0.63739 | CC(=CCC1=C(C=CC2=C1OC3C2COC4=CC(=CC(=C34)OC)O)O)C |
| MOL004961 | Quercetin der. | 46.44939 | 330.31 | 0.3343 | COC1=C(C=CC(=C1)C2=C(C(=O)C3=C(C=C(C=C3O2)O)O)OC)O |
| MOL004966 | 3'-Hydroxy-4'-O-Methylglabridin | 43.71495 | 354.43 | 0.57406 | CC1(C=CC2=C(O1)C=CC3=C2OCC(C3)C4=C(C(=C(C=C4)OC)O)O)C |
| MOL004974 | 3'-Methoxyglabridin | 46.16151 | 354.43 | 0.57393 | CC1(C=CC2=C(O1)C=CC3=C2OCC(C3)C4=C(C(=C(C=C4)O)OC)O)C |
| MOL004978 | 2-[(3R)-8,8-dimethyl-3,4-dihydro-2H-pyrano[6,5-f]chromen-3-yl]-5-methoxyphenol | 36.21429 | 338.43 | 0.52122 | CC1(C=CC2=C(O1)C=CC3=C2OCC(C3)C4=C(C=C(C=C4)OC)O)C |
| MOL004980 | Inflacoumarin A | 39.7091 | 322.38 | 0.32613 | CC(=CCC1=C(C=C2C(=C1)C(=CC(=O)O2)C3=CC=C(C=C3)O)O)C |
| MOL004985 | icos-5-enoic acid | 30.70294 | 310.58 | 0.19725 | CCCCCCCCCCCCCCC=CCCCC(=O)O |
| MOL004988 | Kanzonol F | 32.46833 | 420.54 | 0.89364 | CC(=CCC1=C(C=C2C(=C1OC)C3C(CO2)C4=C(O3)C=C5C(=C4)C=CC(O5)(C)C)O)C |
| MOL004989 | 6-prenylated eriodictyol | 39.22383 | 356.4 | 0.41259 | C1C(OC2=CC(=CC(=C2C1=O)O)OC3C(C(C(C(O3)CO)O)O)O)C4=CC(=C(C=C4)O)O |
| MOL004990 | 7,2',4'-trihydroxy－5-methoxy-3－arylcoumarin | 83.71437 | 300.28 | 0.27136 | COC1=C2C=C(C(=O)OC2=CC(=C1)O)C3=C(C=C(C=C3)O)O |
| MOL004991 | 7-Acetoxy-2-methylisoflavone | 38.92333 | 294.32 | 0.26217 | CC1=C(C(=O)C2=C(O1)C=C(C=C2)OC(=O)C)C3=CC=CC=C3 |
| MOL004993 | 8-prenylated eriodictyol | 53.79476 | 356.4 | 0.40383 | C1C(OC2=CC(=CC(=C2C1=O)O)O)C3=CC(=C(C=C3)O)O |
| MOL004996 | gadelaidic acid | 30.70294 | 310.58 | 0.19725 | CCCCCCCCCCC=CCCCCCCCC(=O)O |
| MOL005000 | Gancaonin G | 60.43521 | 352.41 | 0.39404 | CC(=CCC1=C(C=C2C(=C1O)C(=O)C(=CO2)C3=CC=C(C=C3)O)OC)C |
| MOL005001 | Gancaonin H | 50.10372 | 420.49 | 0.78416 | CC(=CCC1=C(C=C2C(=C1O)C(=O)C(=CO2)C3=CC4=C(C(=C3)O)OC(C=C4)(C)C)O)C |
| MOL005003 | Licoagrocarpin | 58.8139 | 338.43 | 0.58498 | CC(=CCC1=C(C=CC2=C1OCC3C2OC4=C3C=CC(=C4)OC)O)C |
| MOL005007 | Glyasperins M | 72.67081 | 368.41 | 0.59274 | CC1(C=CC2=C(O1)C=CC(=C2O)C3COC4=CC(=CC(=C4C3=O)OC)O)C |
| MOL005008 | Glycyrrhiza flavonol A | 41.27528 | 370.38 | 0.59512 | CC1(C(CC2=C(O1)C=CC(=C2)C3=C(C(=O)C4=C(C=C(C=C4O3)O)O)O)O)C |
| MOL005012 | Licoagroisoflavone | 57.28224 | 336.36 | 0.48679 | CC(=C)C1CC2=C(O1)C=C3C(=C2O)C(=O)C(=CO3)C4=CC=C(C=C4)O |
| MOL005013 | 18α-hydroxyglycyrrhetic acid | 41.16139 | 486.76 | 0.7091 | CC(C)N1CCC2=C(C1)SC(=C2C(=O)OC)NC(=O)C3=CC=C(C=C3)OC.Cl |
| MOL005016 | Odoratin | 49.94822 | 314.31 | 0.30487 | COC1=C(C=C(C=C1)C2=COC3=CC(=C(C=C3C2=O)OC)O)O |
| MOL005017 | Phaseol | 78.76622 | 336.36 | 0.57867 | CC(=CCC1=C(C=CC2=C1OC(=O)C3=C2OC4=C3C=CC(=C4)O)O)C |
| MOL005018 | Xambioona | 54.84916 | 388.49 | 0.87419 | CC1(C=CC2=C(O1)C=CC(=C2)C3CC(=O)C4=C(O3)C5=C(C=C4)OC(C=C5)(C)C)C |
| MOL005020 | dehydroglyasperins C | 53.82326 | 340.4 | 0.37006 | CC1=CC(=C(C=C1)C2=CC3=C(C(=C(C=C3OC2)O)CC=C(C)C)OC)O |

Table S2 Relationship between active compounds and putative targets

| Molecule_ID | Gene_name |
| --- | --- |
| MOL000211 | ESR |
| MOL000211 | AR |
| MOL000211 | PGR |
| MOL000211 | NR3C1 |
| MOL000358 | BCL2 |
| MOL000358 | PON1 |
| MOL000358 | JUN |
| MOL000358 | MAP2 |
| MOL000358 | NOS2 |
| MOL000358 | PTGS1 |
| MOL000358 | DRD1 |
| MOL000358 | CHRM3 |
| MOL000358 | F2 |
| MOL000358 | KCNH2 |
| MOL000358 | CHRM1 |
| MOL000358 | ESR |
| MOL000358 | AR |
| MOL000358 | SCN5A |
| MOL000358 | PPARG |
| MOL000358 | PTGS2 |
| MOL000358 | CA2 |
| MOL000358 | GABRA2 |
| MOL000358 | CHRM4 |
| MOL000358 | ACHE |
| MOL000358 | PDE3A |
| MOL000358 | HTR2A |
| MOL000358 | GABRA5 |
| MOL000358 | ADRA1A |
| MOL000358 | GABRA3 |
| MOL000358 | PGR |
| MOL000358 | CHRM2 |
| MOL000358 | ADRA1B |
| MOL000358 | PTPN1 |
| MOL000358 | ADRB2 |
| MOL000358 | CHRNA2 |
| MOL000358 | SLC6A4 |
| MOL000358 | OPRM1 |
| MOL000358 | ESR2 |
| MOL000358 | NR3C1 |
| MOL000358 | GABRA1 |
| MOL000358 | DPP4 |
| MOL000358 | MAPK14 |
| MOL000358 | GSK3B |
| MOL000358 | HSP90 |
| MOL000358 | CDK2 |
| MOL000358 | PIK3CG |
| MOL000358 | CHRNA7 |
| MOL000358 | CHEK1 |
| MOL000358 | PRKACA |
| MOL000358 | PRSS1 |
| MOL000358 | PIM1 |
| MOL000358 | CCNA2 |
| MOL000358 | NCOA2 |
| MOL000359 | ESR |
| MOL000359 | AR |
| MOL000359 | PGR |
| MOL000359 | NR3C2 |
| MOL000359 | NR3C1 |
| MOL000359 | NCOA2 |
| MOL000422 | NOS2 |
| MOL000422 | INSR |
| MOL000422 | ESR |
| MOL000422 | BCL2 |
| MOL000422 | ALOX5 |
| MOL000422 | PTGS2 |
| MOL000422 | AKR1C3 |
| MOL000422 | TNF |
| MOL000422 | ESR2 |
| MOL000422 | MMP1 |
| MOL000422 | JUN |
| MOL000422 | SELE |
| MOL000422 | CDK1 |
| MOL000422 | VCAM1 |
| MOL000422 | XDH |
| MOL000422 | CYP3A4 |
| MOL000422 | MAPK8 |
| MOL000422 | CYP1A2 |
| MOL000422 | GSTP1 |
| MOL000422 | HMOX1 |
| MOL000422 | GSTM1 |
| MOL000422 | AHR |
| MOL000422 | GSTM2 |
| MOL000422 | PPP3CA |
| MOL000422 | PTGS1 |
| MOL000422 | F2 |
| MOL000422 | CHRM1 |
| MOL000422 | AR |
| MOL000422 | PPARG |
| MOL000422 | NOS3 |
| MOL000422 | CA2 |
| MOL000422 | F7 |
| MOL000422 | GABRA2 |
| MOL000422 | ACHE |
| MOL000422 | SLC6A2 |
| MOL000422 | PGR |
| MOL000422 | CHRM2 |
| MOL000422 | ADRA1B |
| MOL000422 | PTPN1 |
| MOL000422 | TOP2 |
| MOL000422 | GABRA1 |
| MOL000422 | DPP4 |
| MOL000422 | MAPK14 |
| MOL000422 | GSK3B |
| MOL000422 | HSP90 |
| MOL000422 | CDK2 |
| MOL000422 | PIK3CG |
| MOL000422 | CHEK1 |
| MOL000422 | PRKACA |
| MOL000422 | PRSS1 |
| MOL000422 | PIM1 |
| MOL000422 | CCNA2 |
| MOL000422 | NCOA2 |
| MOL000422 | CALM1 |
| MOL000492 | CAT |
| MOL000492 | NOS2 |
| MOL000492 | PTGS1 |
| MOL000492 | ESR |
| MOL000492 | AR |
| MOL000492 | PPARG |
| MOL000492 | PTGS2 |
| MOL000492 | CA2 |
| MOL000492 | RXRA |
| MOL000492 | PTPN1 |
| MOL000492 | ESR2 |
| MOL000492 | DPP4 |
| MOL000492 | MAPK14 |
| MOL000492 | GSK3B |
| MOL000492 | HSP90 |
| MOL000492 | CDK2 |
| MOL000492 | CHEK1 |
| MOL000492 | PRKACA |
| MOL000492 | PIM1 |
| MOL000492 | CCNA2 |
| MOL000492 | NCOA2 |
| MOL000492 | CALM1 |
| MOL001918 | GABRA1 |
| MOL001918 | DPP4 |
| MOL001919 | ESR |
| MOL001919 | AR |
| MOL001919 | PGR |
| MOL001919 | NR3C2 |
| MOL001919 | NR3C1 |
| MOL001924 | TNF |
| MOL001924 | IL6 |
| MOL001928 | DPP4 |
| MOL000098 | NOS2 |
| MOL000098 | PTGS1 |
| MOL000098 | INSR |
| MOL000098 | ESR |
| MOL000098 | AR |
| MOL000098 | BCL2 |
| MOL000098 | ALOX5 |
| MOL000098 | PTGS2 |
| MOL000098 | ODC1 |
| MOL000098 | ACACA |
| MOL000098 | MMP2 |
| MOL000098 | TNF |
| MOL000098 | EGFR |
| MOL000098 | ESR2 |
| MOL000098 | MGAM |
| MOL000098 | PLAU |
| MOL000098 | IL6 |
| MOL000098 | MMP1 |
| MOL000098 | MAPK1 |
| MOL000098 | PON1 |
| MOL000098 | CTSD |
| MOL000098 | IFNG |
| MOL000098 | JUN |
| MOL000098 | CCL2 |
| MOL000098 | IL1B |
| MOL000098 | GSK3B |
| MOL000098 | SELE |
| MOL000098 | MPO |
| MOL000098 | CDK1 |
| MOL000098 | PLAT |
| MOL000098 | GJA1 |
| MOL000098 | VCAM1 |
| MOL000098 | THBD |
| MOL000098 | F3 |
| MOL000098 | NQO1 |
| MOL000098 | XDH |
| MOL000098 | PTGER3 |
| MOL000098 | SOD1 |
| MOL000098 | CYP3A4 |
| MOL000098 | TP53 |
| MOL000098 | CYP1A2 |
| MOL000098 | COL1A1 |
| MOL000098 | GSTP1 |
| MOL000098 | EGF |
| MOL000098 | VEGFA |
| MOL000098 | POR |
| MOL000098 | SULT1E1 |
| MOL000098 | TOP1 |
| MOL000098 | HMOX1 |
| MOL000098 | COL3A1 |
| MOL000098 | RB1 |
| MOL000098 | GSTM1 |
| MOL000098 | HSPA5 |
| MOL000098 | ACPP |
| MOL000098 | AHR |
| MOL000098 | GSTM2 |
| MOL000098 | IL2 |
| MOL000098 | F2 |
| MOL000098 | KCNH2 |
| MOL000098 | SCN5A |
| MOL000098 | PPARG |
| MOL000098 | F10 |
| MOL000098 | NOS3 |
| MOL000098 | CA2 |
| MOL000098 | F7 |
| MOL000098 | RXRA |
| MOL000098 | ACHE |
| MOL000098 | PTPN1 |
| MOL000098 | ADRB2 |
| MOL000098 | TOP2 |
| MOL000098 | AKR1B1 |
| MOL000098 | GABRA1 |
| MOL000098 | DPP4 |
| MOL000098 | MAPK14 |
| MOL000098 | MMP3 |
| MOL000098 | HSP90 |
| MOL000098 | CDK2 |
| MOL000098 | PIK3CG |
| MOL000098 | MAOB |
| MOL000098 | CHEK1 |
| MOL000098 | PRKACA |
| MOL000098 | PRSS1 |
| MOL000098 | PIM1 |
| MOL000098 | CCNA2 |
| MOL000098 | NCOA2 |
| MOL000211 | ESR |
| MOL000211 | AR |
| MOL000211 | PGR |
| MOL000211 | NR3C1 |
| MOL000239 | NOS2 |
| MOL000239 | PTGS1 |
| MOL000239 | ESR |
| MOL000239 | AR |
| MOL000239 | SCN5A |
| MOL000239 | PPARG |
| MOL000239 | PTGS2 |
| MOL000239 | CA2 |
| MOL000239 | PTPN1 |
| MOL000239 | ESR2 |
| MOL000239 | DPP4 |
| MOL000239 | MAPK14 |
| MOL000239 | GSK3B |
| MOL000239 | HSP90 |
| MOL000239 | CDK2 |
| MOL000239 | CHEK1 |
| MOL000239 | PRSS1 |
| MOL000239 | PIM1 |
| MOL000239 | CCNA2 |
| MOL000239 | NCOA2 |
| MOL000239 | CALM1 |
| MOL000354 | NOS2 |
| MOL000354 | XDH |
| MOL000354 | PTGS1 |
| MOL000354 | F2 |
| MOL000354 | ESR |
| MOL000354 | AR |
| MOL000354 | PPARG |
| MOL000354 | PTGS2 |
| MOL000354 | NOS3 |
| MOL000354 | CA2 |
| MOL000354 | F7 |
| MOL000354 | ACHE |
| MOL000354 | PTPN1 |
| MOL000354 | AKR1B1 |
| MOL000354 | ESR2 |
| MOL000354 | GABRA1 |
| MOL000354 | DPP4 |
| MOL000354 | PYGM |
| MOL000354 | PPARD |
| MOL000354 | MAPK14 |
| MOL000354 | GSK3B |
| MOL000354 | HSP90 |
| MOL000354 | CDK2 |
| MOL000354 | PIK3CG |
| MOL000354 | MAOB |
| MOL000354 | CHEK1 |
| MOL000354 | PRKACA |
| MOL000354 | PRSS1 |
| MOL000354 | PIM1 |
| MOL000354 | CCNA2 |
| MOL000354 | GRIA2 |
| MOL000354 | NCOA2 |
| MOL000354 | NCOA1 |
| MOL000354 | CALM1 |
| MOL000359 | ESR |
| MOL000359 | AR |
| MOL000359 | PGR |
| MOL000359 | NR3C2 |
| MOL000359 | NR3C1 |
| MOL000359 | NCOA2 |
| MOL000392 | ESR |
| MOL000392 | ESR2 |
| MOL000392 | JUN |
| MOL000392 | MT-ND6 |
| MOL000392 | ATP5B |
| MOL000392 | NOS2 |
| MOL000392 | PTGS1 |
| MOL000392 | F2 |
| MOL000392 | CHRM1 |
| MOL000392 | AR |
| MOL000392 | PPARG |
| MOL000392 | PTGS2 |
| MOL000392 | NOS3 |
| MOL000392 | CA2 |
| MOL000392 | RXRA |
| MOL000392 | ACHE |
| MOL000392 | PDE3A |
| MOL000392 | ADRA1A |
| MOL000392 | PTPN1 |
| MOL000392 | SLC6A3 |
| MOL000392 | ADRB2 |
| MOL000392 | SLC6A4 |
| MOL000392 | DPP4 |
| MOL000392 | MAPK14 |
| MOL000392 | GSK3B |
| MOL000392 | HSP90 |
| MOL000392 | CDK2 |
| MOL000392 | MAOB |
| MOL000392 | CHEK1 |
| MOL000392 | PRKACA |
| MOL000392 | PRSS1 |
| MOL000392 | PIM1 |
| MOL000392 | CCNA2 |
| MOL000392 | PKIA |
| MOL000392 | CALM1 |
| MOL000417 | NOS2 |
| MOL000417 | PTGS1 |
| MOL000417 | ESR |
| MOL000417 | AR |
| MOL000417 | PPARG |
| MOL000417 | PTGS2 |
| MOL000417 | CA2 |
| MOL000417 | RXRA |
| MOL000417 | PDE3A |
| MOL000417 | PTPN1 |
| MOL000417 | ADRB2 |
| MOL000417 | ESR2 |
| MOL000417 | DPP4 |
| MOL000417 | MAPK14 |
| MOL000417 | GSK3B |
| MOL000417 | HSP90 |
| MOL000417 | CDK2 |
| MOL000417 | CHEK1 |
| MOL000417 | PRKACA |
| MOL000417 | PRSS1 |
| MOL000417 | PIM1 |
| MOL000417 | CCNA2 |
| MOL000417 | NCOA2 |
| MOL000417 | CALM1 |
| MOL000422 | NOS2 |
| MOL000422 | INSR |
| MOL000422 | ESR |
| MOL000422 | BCL2 |
| MOL000422 | ALOX5 |
| MOL000422 | PTGS2 |
| MOL000422 | AKR1C3 |
| MOL000422 | TNF |
| MOL000422 | ESR2 |
| MOL000422 | MMP1 |
| MOL000422 | JUN |
| MOL000422 | SELE |
| MOL000422 | CDK1 |
| MOL000422 | VCAM1 |
| MOL000422 | XDH |
| MOL000422 | CYP3A4 |
| MOL000422 | MAPK8 |
| MOL000422 | CYP1A2 |
| MOL000422 | GSTP1 |
| MOL000422 | HMOX1 |
| MOL000422 | GSTM1 |
| MOL000422 | AHR |
| MOL000422 | GSTM2 |
| MOL000422 | PPP3CA |
| MOL000422 | PTGS1 |
| MOL000422 | F2 |
| MOL000422 | CHRM1 |
| MOL000422 | AR |
| MOL000422 | PPARG |
| MOL000422 | NOS3 |
| MOL000422 | CA2 |
| MOL000422 | F7 |
| MOL000422 | GABRA2 |
| MOL000422 | ACHE |
| MOL000422 | SLC6A2 |
| MOL000422 | PGR |
| MOL000422 | CHRM2 |
| MOL000422 | ADRA1B |
| MOL000422 | PTPN1 |
| MOL000422 | TOP2 |
| MOL000422 | GABRA1 |
| MOL000422 | DPP4 |
| MOL000422 | MAPK14 |
| MOL000422 | GSK3B |
| MOL000422 | HSP90 |
| MOL000422 | CDK2 |
| MOL000422 | PIK3CG |
| MOL000422 | CHEK1 |
| MOL000422 | PRKACA |
| MOL000422 | PRSS1 |
| MOL000422 | PIM1 |
| MOL000422 | CCNA2 |
| MOL000422 | NCOA2 |
| MOL000422 | CALM1 |
| MOL000497 | BCL2 |
| MOL000497 | MAPK1 |
| MOL000497 | CDK2 |
| MOL000497 | CDK4 |
| MOL000497 | RB1 |
| MOL000497 | CCNA2 |
| MOL000497 | NOS2 |
| MOL000497 | PTGS1 |
| MOL000497 | F2 |
| MOL000497 | CHRM1 |
| MOL000497 | ESR |
| MOL000497 | AR |
| MOL000497 | SCN5A |
| MOL000497 | PPARG |
| MOL000497 | F10 |
| MOL000497 | PTGS2 |
| MOL000497 | CA2 |
| MOL000497 | ADRA1B |
| MOL000497 | PTPN1 |
| MOL000497 | SLC6A3 |
| MOL000497 | ADRB2 |
| MOL000497 | ESR2 |
| MOL000497 | DPP4 |
| MOL000497 | MAPK14 |
| MOL000497 | GSK3B |
| MOL000497 | HSP90 |
| MOL000497 | CHEK1 |
| MOL000497 | PRSS1 |
| MOL000497 | PIM1 |
| MOL000497 | NCOA2 |
| MOL000497 | CALM1 |
| MOL000500 | NOS2 |
| MOL000500 | PTGS1 |
| MOL000500 | CHRM1 |
| MOL000500 | ESR |
| MOL000500 | AR |
| MOL000500 | SCN5A |
| MOL000500 | PPARG |
| MOL000500 | PTGS2 |
| MOL000500 | CA2 |
| MOL000500 | CHRM4 |
| MOL000500 | RXRA |
| MOL000500 | PDE3A |
| MOL000500 | HTR2A |
| MOL000500 | ADRA1A |
| MOL000500 | ADRA1B |
| MOL000500 | PTPN1 |
| MOL000500 | SLC6A3 |
| MOL000500 | ADRB2 |
| MOL000500 | SLC6A4 |
| MOL000500 | ESR2 |
| MOL000500 | DPP4 |
| MOL000500 | MAPK14 |
| MOL000500 | GSK3B |
| MOL000500 | HSP90 |
| MOL000500 | CDK2 |
| MOL000500 | CHEK1 |
| MOL000500 | PRKACA |
| MOL000500 | PRSS1 |
| MOL000500 | PIM1 |
| MOL000500 | CCNA2 |
| MOL000500 | PKIA |
| MOL000500 | CALM1 |
| MOL001484 | NOS2 |
| MOL001484 | PTGS1 |
| MOL001484 | CHRM3 |
| MOL001484 | CHRM1 |
| MOL001484 | ESR |
| MOL001484 | AR |
| MOL001484 | SCN5A |
| MOL001484 | PPARG |
| MOL001484 | PTGS2 |
| MOL001484 | HTR3A |
| MOL001484 | CA2 |
| MOL001484 | RXRA |
| MOL001484 | ADRA1B |
| MOL001484 | PTPN1 |
| MOL001484 | ADRB2 |
| MOL001484 | ADRA1D |
| MOL001484 | OPRM1 |
| MOL001484 | ESR2 |
| MOL001484 | DPP4 |
| MOL001484 | MAPK14 |
| MOL001484 | GSK3B |
| MOL001484 | HSP90 |
| MOL001484 | CDK2 |
| MOL001484 | PIK3CG |
| MOL001484 | CHEK1 |
| MOL001484 | PRKACA |
| MOL001484 | IGHG1 |
| MOL001484 | PRSS1 |
| MOL001484 | PIM1 |
| MOL001484 | CCNA2 |
| MOL001484 | CALM1 |
| MOL001792 | NOS2 |
| MOL001792 | PTGS1 |
| MOL001792 | ESR |
| MOL001792 | AR |
| MOL001792 | PPARG |
| MOL001792 | PTGS2 |
| MOL001792 | CA2 |
| MOL001792 | RXRA |
| MOL001792 | PTPN1 |
| MOL001792 | ADRB2 |
| MOL001792 | SLC6A4 |
| MOL001792 | ESR2 |
| MOL001792 | DPP4 |
| MOL001792 | MAPK14 |
| MOL001792 | GSK3B |
| MOL001792 | HSP90 |
| MOL001792 | CDK2 |
| MOL001792 | PIK3CG |
| MOL001792 | MAOB |
| MOL001792 | PRKACA |
| MOL001792 | PIM1 |
| MOL001792 | CCNA2 |
| MOL001792 | PKIA |
| MOL002311 | NOS2 |
| MOL002311 | F2 |
| MOL002311 | ESR |
| MOL002311 | PPARG |
| MOL002311 | PTGS2 |
| MOL002311 | KDR |
| MOL002311 | PTPN1 |
| MOL002311 | MAPK14 |
| MOL002311 | GSK3B |
| MOL002311 | CHEK1 |
| MOL002311 | PIM1 |
| MOL002311 | CCNA2 |
| MOL002565 | NOS2 |
| MOL002565 | PTGS1 |
| MOL002565 | DRD1 |
| MOL002565 | CHRM3 |
| MOL002565 | CHRM1 |
| MOL002565 | ESR |
| MOL002565 | AR |
| MOL002565 | SCN5A |
| MOL002565 | PPARG |
| MOL002565 | CHRM5 |
| MOL002565 | PTGS2 |
| MOL002565 | CA2 |
| MOL002565 | CHRM4 |
| MOL002565 | RXRA |
| MOL002565 | OPRD1 |
| MOL002565 | PDE3A |
| MOL002565 | HTR2A |
| MOL002565 | ADRA1A |
| MOL002565 | CHRM2 |
| MOL002565 | ADRA1B |
| MOL002565 | PTPN1 |
| MOL002565 | SLC6A3 |
| MOL002565 | ADRB2 |
| MOL002565 | ADRA1D |
| MOL002565 | SLC6A4 |
| MOL002565 | OPRM1 |
| MOL002565 | ESR2 |
| MOL002565 | DPP4 |
| MOL002565 | MAPK14 |
| MOL002565 | GSK3B |
| MOL002565 | MAPK10 |
| MOL002565 | HSP90 |
| MOL002565 | CDK2 |
| MOL002565 | PIK3CG |
| MOL002565 | CHRNA7 |
| MOL002565 | CHEK1 |
| MOL002565 | PRKACA |
| MOL002565 | PRSS1 |
| MOL002565 | PIM1 |
| MOL002565 | CCNA2 |
| MOL002565 | CALM1 |
| MOL003656 | NOS2 |
| MOL003656 | F2 |
| MOL003656 | ESR |
| MOL003656 | AR |
| MOL003656 | SCN5A |
| MOL003656 | PPARG |
| MOL003656 | F10 |
| MOL003656 | PTGS2 |
| MOL003656 | CA2 |
| MOL003656 | PTPN1 |
| MOL003656 | TOP2 |
| MOL003656 | ESR2 |
| MOL003656 | DPP4 |
| MOL003656 | MAPK14 |
| MOL003656 | GSK3B |
| MOL003656 | HSP90 |
| MOL003656 | CDK2 |
| MOL003656 | CHEK1 |
| MOL003656 | PRSS1 |
| MOL003656 | PIM1 |
| MOL003656 | CCNA2 |
| MOL003656 | NCOA2 |
| MOL003656 | CALM1 |
| MOL003896 | NOS2 |
| MOL003896 | PTGS1 |
| MOL003896 | DRD1 |
| MOL003896 | CHRM3 |
| MOL003896 | F2 |
| MOL003896 | CHRM1 |
| MOL003896 | ESR |
| MOL003896 | AR |
| MOL003896 | ADRB1 |
| MOL003896 | SCN5A |
| MOL003896 | PPARG |
| MOL003896 | CHRM5 |
| MOL003896 | PTGS2 |
| MOL003896 | NOS3 |
| MOL003896 | CA2 |
| MOL003896 | RXRA |
| MOL003896 | ACHE |
| MOL003896 | PDE3A |
| MOL003896 | ADRA1B |
| MOL003896 | PTPN1 |
| MOL003896 | SLC6A3 |
| MOL003896 | ADRB2 |
| MOL003896 | ADRA1D |
| MOL003896 | SLC6A4 |
| MOL003896 | OPRM1 |
| MOL003896 | ESR2 |
| MOL003896 | GABRA1 |
| MOL003896 | DPP4 |
| MOL003896 | MAPK14 |
| MOL003896 | GSK3B |
| MOL003896 | HSP90 |
| MOL003896 | CDK2 |
| MOL003896 | LTA4H |
| MOL003896 | MAOB |
| MOL003896 | CHRNA7 |
| MOL003896 | CHEK1 |
| MOL003896 | PRKACA |
| MOL003896 | IGHG1 |
| MOL003896 | PRSS1 |
| MOL003896 | PIM1 |
| MOL003896 | CCNA2 |
| MOL003896 | NCOA2 |
| MOL003896 | NCOA1 |
| MOL003896 | PKIA |
| MOL003896 | CALM1 |
| MOL004328 | NOS2 |
| MOL004328 | ESR |
| MOL004328 | BCL2 |
| MOL004328 | ABAT |
| MOL004328 | LDLR |
| MOL004328 | GOT1 |
| MOL004328 | GSR |
| MOL004328 | ABCC1 |
| MOL004328 | ESR2 |
| MOL004328 | MAPK1 |
| MOL004328 | FASN |
| MOL004328 | MAPK3 |
| MOL004328 | SOD1 |
| MOL004328 | GSTP1 |
| MOL004328 | CAT |
| MOL004328 | HMGCR |
| MOL004328 | SOAT1 |
| MOL004328 | AKR1C1 |
| MOL004328 | PTGS1 |
| MOL004328 | AR |
| MOL004328 | PPARG |
| MOL004328 | PTGS2 |
| MOL004328 | CA2 |
| MOL004328 | PTPN1 |
| MOL004328 | DPP4 |
| MOL004328 | MAPK14 |
| MOL004328 | GSK3B |
| MOL004328 | HSP90 |
| MOL004328 | CDK2 |
| MOL004328 | PIK3CG |
| MOL004328 | #N/A |
| MOL004328 | PRKACA |
| MOL004328 | CCNA2 |
| MOL004805 | NOS2 |
| MOL004805 | F2 |
| MOL004805 | KCNH2 |
| MOL004805 | ESR |
| MOL004805 | AR |
| MOL004805 | PPARG |
| MOL004805 | F10 |
| MOL004805 | PTGS2 |
| MOL004805 | CA2 |
| MOL004805 | PTPN1 |
| MOL004805 | ESR2 |
| MOL004805 | DPP4 |
| MOL004805 | MAPK14 |
| MOL004805 | GSK3B |
| MOL004805 | CHEK1 |
| MOL004805 | PRSS1 |
| MOL004805 | PIM1 |
| MOL004805 | CALM1 |
| MOL004806 | NOS2 |
| MOL004806 | F2 |
| MOL004806 | KCNH2 |
| MOL004806 | ESR |
| MOL004806 | AR |
| MOL004806 | SCN5A |
| MOL004806 | PPARG |
| MOL004806 | F10 |
| MOL004806 | PTGS2 |
| MOL004806 | PTPN1 |
| MOL004806 | ESR2 |
| MOL004806 | DPP4 |
| MOL004806 | BACE1 |
| MOL004806 | MAPK14 |
| MOL004806 | PRSS1 |
| MOL004806 | PIM1 |
| MOL004806 | CALM1 |
| MOL004808 | NOS2 |
| MOL004808 | F2 |
| MOL004808 | ESR |
| MOL004808 | AR |
| MOL004808 | PPARG |
| MOL004808 | F10 |
| MOL004808 | PTGS2 |
| MOL004808 | CA2 |
| MOL004808 | F7 |
| MOL004808 | KDR |
| MOL004808 | ACHE |
| MOL004808 | PTPN1 |
| MOL004808 | TOP2 |
| MOL004808 | ESR2 |
| MOL004808 | DPP4 |
| MOL004808 | GSK3B |
| MOL004808 | HSP90 |
| MOL004808 | CDK2 |
| MOL004808 | CHEK1 |
| MOL004808 | PRSS1 |
| MOL004808 | PIM1 |
| MOL004808 | CCNA2 |
| MOL004808 | NCOA2 |
| MOL004808 | CALM1 |
| MOL004810 | NOS2 |
| MOL004810 | PTGS1 |
| MOL004810 | F2 |
| MOL004810 | ESR |
| MOL004810 | AR |
| MOL004810 | SCN5A |
| MOL004810 | PPARG |
| MOL004810 | F10 |
| MOL004810 | PTGS2 |
| MOL004810 | CA2 |
| MOL004810 | PTPN1 |
| MOL004810 | TOP2 |
| MOL004810 | ESR2 |
| MOL004810 | DPP4 |
| MOL004810 | MAPK14 |
| MOL004810 | GSK3B |
| MOL004810 | HSP90 |
| MOL004810 | CDK2 |
| MOL004810 | CHEK1 |
| MOL004810 | PRSS1 |
| MOL004810 | PIM1 |
| MOL004810 | CCNA2 |
| MOL004810 | CALM1 |
| MOL004811 | NOS2 |
| MOL004811 | F2 |
| MOL004811 | KCNH2 |
| MOL004811 | ESR |
| MOL004811 | AR |
| MOL004811 | SCN5A |
| MOL004811 | PPARG |
| MOL004811 | F10 |
| MOL004811 | PTGS2 |
| MOL004811 | CA2 |
| MOL004811 | RXRA |
| MOL004811 | ACHE |
| MOL004811 | PTPN1 |
| MOL004811 | TOP2 |
| MOL004811 | ESR2 |
| MOL004811 | DPP4 |
| MOL004811 | MAPK14 |
| MOL004811 | GSK3B |
| MOL004811 | HSP90 |
| MOL004811 | CDK2 |
| MOL004811 | CHEK1 |
| MOL004811 | PRSS1 |
| MOL004811 | PIM1 |
| MOL004811 | CCNA2 |
| MOL004811 | NCOA2 |
| MOL004811 | CALM1 |
| MOL004814 | NOS2 |
| MOL004814 | ESR |
| MOL004814 | AR |
| MOL004814 | PTGS2 |
| MOL004814 | PTPN1 |
| MOL004814 | ESR2 |
| MOL004814 | MAPK14 |
| MOL004814 | GSK3B |
| MOL004814 | HSP90 |
| MOL004814 | CDK2 |
| MOL004814 | PIK3CG |
| MOL004814 | CHEK1 |
| MOL004814 | PRKACA |
| MOL004814 | PIM1 |
| MOL004814 | CCNA2 |
| MOL004815 | NOS2 |
| MOL004815 | PTGS1 |
| MOL004815 | F2 |
| MOL004815 | ESR |
| MOL004815 | AR |
| MOL004815 | SCN5A |
| MOL004815 | PPARG |
| MOL004815 | F10 |
| MOL004815 | PTGS2 |
| MOL004815 | CA2 |
| MOL004815 | RXRA |
| MOL004815 | ACHE |
| MOL004815 | ADRA1B |
| MOL004815 | ESR2 |
| MOL004815 | DPP4 |
| MOL004815 | MAPK14 |
| MOL004815 | GSK3B |
| MOL004815 | CDK2 |
| MOL004815 | CHEK1 |
| MOL004815 | PRSS1 |
| MOL004815 | PIM1 |
| MOL004815 | CCNA2 |
| MOL004815 | NCOA2 |
| MOL004815 | CALM1 |
| MOL004820 | NOS2 |
| MOL004820 | PTGS1 |
| MOL004820 | F2 |
| MOL004820 | ESR |
| MOL004820 | AR |
| MOL004820 | SCN5A |
| MOL004820 | PPARG |
| MOL004820 | F10 |
| MOL004820 | PTGS2 |
| MOL004820 | CA2 |
| MOL004820 | RXRA |
| MOL004820 | ACHE |
| MOL004820 | PTPN1 |
| MOL004820 | TOP2 |
| MOL004820 | ESR2 |
| MOL004820 | MAPK14 |
| MOL004820 | GSK3B |
| MOL004820 | CDK2 |
| MOL004820 | CHEK1 |
| MOL004820 | PRSS1 |
| MOL004820 | PIM1 |
| MOL004820 | CCNA2 |
| MOL004820 | NCOA2 |
| MOL004820 | NCOA1 |
| MOL004820 | CALM1 |
| MOL004824 | NOS2 |
| MOL004824 | F2 |
| MOL004824 | ESR |
| MOL004824 | AR |
| MOL004824 | PPARG |
| MOL004824 | F10 |
| MOL004824 | PTGS2 |
| MOL004824 | CA2 |
| MOL004824 | F7 |
| MOL004824 | KDR |
| MOL004824 | ACHE |
| MOL004824 | PTPN1 |
| MOL004824 | TOP2 |
| MOL004824 | ESR2 |
| MOL004824 | DPP4 |
| MOL004824 | MAPK14 |
| MOL004824 | GSK3B |
| MOL004824 | CDK2 |
| MOL004824 | CHEK1 |
| MOL004824 | PRSS1 |
| MOL004824 | PIM1 |
| MOL004824 | CCNA2 |
| MOL004824 | CALM1 |
| MOL004827 | NOS2 |
| MOL004827 | F2 |
| MOL004827 | ESR |
| MOL004827 | AR |
| MOL004827 | SCN5A |
| MOL004827 | PPARG |
| MOL004827 | F10 |
| MOL004827 | PTGS2 |
| MOL004827 | CA2 |
| MOL004827 | F7 |
| MOL004827 | ACHE |
| MOL004827 | PTPN1 |
| MOL004827 | TOP2 |
| MOL004827 | GSK3B |
| MOL004827 | HSP90 |
| MOL004827 | CDK2 |
| MOL004827 | CHEK1 |
| MOL004827 | PRSS1 |
| MOL004827 | PIM1 |
| MOL004827 | CCNA2 |
| MOL004827 | CALM1 |
| MOL004828 | NOS2 |
| MOL004828 | PTGS1 |
| MOL004828 | F2 |
| MOL004828 | ESR |
| MOL004828 | AR |
| MOL004828 | SCN5A |
| MOL004828 | PPARG |
| MOL004828 | F10 |
| MOL004828 | PTGS2 |
| MOL004828 | NOS3 |
| MOL004828 | CA2 |
| MOL004828 | F7 |
| MOL004828 | KDR |
| MOL004828 | RXRA |
| MOL004828 | PDE3A |
| MOL004828 | PTPN1 |
| MOL004828 | TOP2 |
| MOL004828 | ESR2 |
| MOL004828 | DPP4 |
| MOL004828 | MAPK14 |
| MOL004828 | GSK3B |
| MOL004828 | HSP90 |
| MOL004828 | CDK2 |
| MOL004828 | CHEK1 |
| MOL004828 | IGHG1 |
| MOL004828 | PRSS1 |
| MOL004828 | PIM1 |
| MOL004828 | CCNA2 |
| MOL004828 | CALM1 |
| MOL004829 | NOS2 |
| MOL004829 | PTGS1 |
| MOL004829 | F2 |
| MOL004829 | ESR |
| MOL004829 | AR |
| MOL004829 | SCN5A |
| MOL004829 | PPARG |
| MOL004829 | F10 |
| MOL004829 | PTGS2 |
| MOL004829 | NOS3 |
| MOL004829 | CA2 |
| MOL004829 | F7 |
| MOL004829 | RXRA |
| MOL004829 | PDE3A |
| MOL004829 | ADRA1B |
| MOL004829 | PTPN1 |
| MOL004829 | TOP2 |
| MOL004829 | ESR2 |
| MOL004829 | DPP4 |
| MOL004829 | MAPK14 |
| MOL004829 | GSK3B |
| MOL004829 | HSP90 |
| MOL004829 | CDK2 |
| MOL004829 | CHEK1 |
| MOL004829 | IGHG1 |
| MOL004829 | PRSS1 |
| MOL004829 | PIM1 |
| MOL004829 | NCOA1 |
| MOL004829 | CALM1 |
| MOL004833 | NOS2 |
| MOL004833 | F2 |
| MOL004833 | CHRM1 |
| MOL004833 | ESR |
| MOL004833 | AR |
| MOL004833 | SCN5A |
| MOL004833 | PPARG |
| MOL004833 | F10 |
| MOL004833 | PTGS2 |
| MOL004833 | CA2 |
| MOL004833 | RXRA |
| MOL004833 | ACHE |
| MOL004833 | ADRA1B |
| MOL004833 | PTPN1 |
| MOL004833 | ADRB2 |
| MOL004833 | ESR2 |
| MOL004833 | DPP4 |
| MOL004833 | MAPK14 |
| MOL004833 | GSK3B |
| MOL004833 | CDK2 |
| MOL004833 | CHEK1 |
| MOL004833 | PRSS1 |
| MOL004833 | PIM1 |
| MOL004833 | CCNA2 |
| MOL004833 | NCOA1 |
| MOL004833 | CALM1 |
| MOL004835 | NOS2 |
| MOL004835 | PTGS1 |
| MOL004835 | F2 |
| MOL004835 | CHRM1 |
| MOL004835 | ESR |
| MOL004835 | AR |
| MOL004835 | SCN5A |
| MOL004835 | PPARG |
| MOL004835 | PTGS2 |
| MOL004835 | CA2 |
| MOL004835 | PDE3A |
| MOL004835 | ADRA1B |
| MOL004835 | SLC6A3 |
| MOL004835 | ADRB2 |
| MOL004835 | SLC6A4 |
| MOL004835 | ESR2 |
| MOL004835 | DPP4 |
| MOL004835 | MAPK14 |
| MOL004835 | GSK3B |
| MOL004835 | HSP90 |
| MOL004835 | CDK2 |
| MOL004835 | LTA4H |
| MOL004835 | MAOB |
| MOL004835 | CHEK1 |
| MOL004835 | PRKACA |
| MOL004835 | PRSS1 |
| MOL004835 | CCNA2 |
| MOL004835 | NCOA1 |
| MOL004835 | PKIA |
| MOL004835 | CALM1 |
| MOL004838 | NOS2 |
| MOL004838 | ESR |
| MOL004838 | AR |
| MOL004838 | PPARG |
| MOL004838 | PTGS2 |
| MOL004838 | RXRA |
| MOL004838 | PTPN1 |
| MOL004838 | ESR2 |
| MOL004838 | MAPK14 |
| MOL004838 | GSK3B |
| MOL004838 | HSP90 |
| MOL004838 | CDK2 |
| MOL004838 | PIK3CG |
| MOL004838 | CHEK1 |
| MOL004838 | PIM1 |
| MOL004838 | CCNA2 |
| MOL004841 | NOS2 |
| MOL004841 | PTGS1 |
| MOL004841 | F2 |
| MOL004841 | ESR |
| MOL004841 | AR |
| MOL004841 | PPARG |
| MOL004841 | PTGS2 |
| MOL004841 | CA2 |
| MOL004841 | PDE3A |
| MOL004841 | PTPN1 |
| MOL004841 | ADRB2 |
| MOL004841 | ESR2 |
| MOL004841 | DPP4 |
| MOL004841 | MAPK14 |
| MOL004841 | GSK3B |
| MOL004841 | HSP90 |
| MOL004841 | CDK2 |
| MOL004841 | CHEK1 |
| MOL004841 | PRKACA |
| MOL004841 | PRSS1 |
| MOL004841 | PIM1 |
| MOL004841 | CCNA2 |
| MOL004841 | CALM1 |
| MOL004848 | NOS2 |
| MOL004848 | F2 |
| MOL004848 | ESR |
| MOL004848 | AR |
| MOL004848 | PPARG |
| MOL004848 | F10 |
| MOL004848 | PTGS2 |
| MOL004848 | KDR |
| MOL004848 | PTPN1 |
| MOL004848 | ESR2 |
| MOL004848 | DPP4 |
| MOL004848 | MAPK14 |
| MOL004848 | GSK3B |
| MOL004848 | HSP90 |
| MOL004848 | CDK2 |
| MOL004848 | IGHG1 |
| MOL004848 | PRSS1 |
| MOL004848 | PIM1 |
| MOL004848 | CCNA2 |
| MOL004848 | NCOA2 |
| MOL004848 | CALM1 |
| MOL004849 | NOS2 |
| MOL004849 | F2 |
| MOL004849 | KCNH2 |
| MOL004849 | ESR |
| MOL004849 | AR |
| MOL004849 | PPARG |
| MOL004849 | F10 |
| MOL004849 | PTGS2 |
| MOL004849 | CA2 |
| MOL004849 | F7 |
| MOL004849 | KDR |
| MOL004849 | PTPN1 |
| MOL004849 | TOP2 |
| MOL004849 | ESR2 |
| MOL004849 | DPP4 |
| MOL004849 | MAPK14 |
| MOL004849 | GSK3B |
| MOL004849 | HSP90 |
| MOL004849 | CDK2 |
| MOL004849 | CHEK1 |
| MOL004849 | PRSS1 |
| MOL004849 | PIM1 |
| MOL004849 | NCOA2 |
| MOL004849 | NCOA1 |
| MOL004849 | CALM1 |
| MOL004855 | NOS2 |
| MOL004855 | F2 |
| MOL004855 | KCNH2 |
| MOL004855 | ESR |
| MOL004855 | AR |
| MOL004855 | PPARG |
| MOL004855 | F10 |
| MOL004855 | PTGS2 |
| MOL004855 | CA2 |
| MOL004855 | KDR |
| MOL004855 | PTPN1 |
| MOL004855 | TOP2 |
| MOL004855 | CHEK1 |
| MOL004855 | PRSS1 |
| MOL004855 | PIM1 |
| MOL004855 | NCOA2 |
| MOL004855 | CALM1 |
| MOL004856 | NOS2 |
| MOL004856 | F2 |
| MOL004856 | ESR |
| MOL004856 | AR |
| MOL004856 | SCN5A |
| MOL004856 | PPARG |
| MOL004856 | F10 |
| MOL004856 | PTGS2 |
| MOL004856 | CA2 |
| MOL004856 | ACHE |
| MOL004856 | PTPN1 |
| MOL004856 | TOP2 |
| MOL004856 | ESR2 |
| MOL004856 | DPP4 |
| MOL004856 | GSK3B |
| MOL004856 | HSP90 |
| MOL004856 | CHEK1 |
| MOL004856 | PRSS1 |
| MOL004856 | PIM1 |
| MOL004856 | CCNA2 |
| MOL004856 | NCOA2 |
| MOL004856 | CALM1 |
| MOL004857 | NOS2 |
| MOL004857 | F2 |
| MOL004857 | ESR |
| MOL004857 | AR |
| MOL004857 | PPARG |
| MOL004857 | F10 |
| MOL004857 | PTGS2 |
| MOL004857 | CA2 |
| MOL004857 | F7 |
| MOL004857 | KDR |
| MOL004857 | ADRA1B |
| MOL004857 | PTPN1 |
| MOL004857 | ADRB2 |
| MOL004857 | TOP2 |
| MOL004857 | ESR2 |
| MOL004857 | DPP4 |
| MOL004857 | GSK3B |
| MOL004857 | HSP90 |
| MOL004857 | CHEK1 |
| MOL004857 | PRSS1 |
| MOL004857 | PIM1 |
| MOL004857 | CCNA2 |
| MOL004857 | NCOA2 |
| MOL004857 | CALM1 |
| MOL004863 | NOS2 |
| MOL004863 | F2 |
| MOL004863 | ESR |
| MOL004863 | AR |
| MOL004863 | PPARG |
| MOL004863 | F10 |
| MOL004863 | PTGS2 |
| MOL004863 | PTPN1 |
| MOL004863 | MAPK14 |
| MOL004863 | GSK3B |
| MOL004863 | HSP90 |
| MOL004863 | CDK2 |
| MOL004863 | CHEK1 |
| MOL004863 | PRSS1 |
| MOL004863 | PIM1 |
| MOL004863 | CCNA2 |
| MOL004863 | NCOA2 |
| MOL004863 | CALM1 |
| MOL004864 | NOS2 |
| MOL004864 | KCNH2 |
| MOL004864 | ESR |
| MOL004864 | AR |
| MOL004864 | PPARG |
| MOL004864 | F10 |
| MOL004864 | PTGS2 |
| MOL004864 | CA2 |
| MOL004864 | PTPN1 |
| MOL004864 | TOP2 |
| MOL004864 | ESR2 |
| MOL004864 | DPP4 |
| MOL004864 | MAPK14 |
| MOL004864 | GSK3B |
| MOL004864 | HSP90 |
| MOL004864 | CDK2 |
| MOL004864 | CHEK1 |
| MOL004864 | PRSS1 |
| MOL004864 | PIM1 |
| MOL004864 | CCNA2 |
| MOL004864 | NCOA2 |
| MOL004864 | CALM1 |
| MOL004866 | NOS2 |
| MOL004866 | F2 |
| MOL004866 | ESR |
| MOL004866 | AR |
| MOL004866 | SCN5A |
| MOL004866 | PPARG |
| MOL004866 | F10 |
| MOL004866 | PTGS2 |
| MOL004866 | CA2 |
| MOL004866 | F7 |
| MOL004866 | PTPN1 |
| MOL004866 | ADRB2 |
| MOL004866 | DPP4 |
| MOL004866 | GSK3B |
| MOL004866 | HSP90 |
| MOL004866 | CDK2 |
| MOL004866 | CHEK1 |
| MOL004866 | PRSS1 |
| MOL004866 | PIM1 |
| MOL004866 | CCNA2 |
| MOL004866 | CALM1 |
| MOL004879 | NOS2 |
| MOL004879 | F2 |
| MOL004879 | KCNH2 |
| MOL004879 | ESR |
| MOL004879 | AR |
| MOL004879 | PPARG |
| MOL004879 | F10 |
| MOL004879 | PTGS2 |
| MOL004879 | CA2 |
| MOL004879 | KDR |
| MOL004879 | TOP2 |
| MOL004879 | ESR2 |
| MOL004879 | DPP4 |
| MOL004879 | CHEK1 |
| MOL004879 | PRSS1 |
| MOL004879 | PIM1 |
| MOL004879 | NCOA2 |
| MOL004879 | CALM1 |
| MOL004882 | ESR |
| MOL004882 | AR |
| MOL004882 | PPARG |
| MOL004882 | PTPN1 |
| MOL004882 | ESR2 |
| MOL004882 | MAPK14 |
| MOL004882 | GSK3B |
| MOL004882 | HSP90 |
| MOL004882 | CDK2 |
| MOL004882 | CHEK1 |
| MOL004882 | CCNA2 |
| MOL004883 | NOS2 |
| MOL004883 | F2 |
| MOL004883 | ESR |
| MOL004883 | AR |
| MOL004883 | PPARG |
| MOL004883 | F10 |
| MOL004883 | PTGS2 |
| MOL004883 | KDR |
| MOL004883 | PTPN1 |
| MOL004883 | TOP2 |
| MOL004883 | DPP4 |
| MOL004883 | MAPK14 |
| MOL004883 | HSP90 |
| MOL004883 | CDK2 |
| MOL004883 | CHEK1 |
| MOL004883 | PRSS1 |
| MOL004883 | PIM1 |
| MOL004883 | CCNA2 |
| MOL004883 | NCOA2 |
| MOL004883 | CALM1 |
| MOL004884 | NOS2 |
| MOL004884 | F2 |
| MOL004884 | ESR |
| MOL004884 | AR |
| MOL004884 | PPARG |
| MOL004884 | F10 |
| MOL004884 | PTGS2 |
| MOL004884 | CA2 |
| MOL004884 | ACHE |
| MOL004884 | PTPN1 |
| MOL004884 | TOP2 |
| MOL004884 | ESR2 |
| MOL004884 | GSK3B |
| MOL004884 | CDK2 |
| MOL004884 | CHEK1 |
| MOL004884 | PRSS1 |
| MOL004884 | PIM1 |
| MOL004884 | CCNA2 |
| MOL004884 | CALM1 |
| MOL004885 | NOS2 |
| MOL004885 | PTGS1 |
| MOL004885 | F2 |
| MOL004885 | ESR |
| MOL004885 | AR |
| MOL004885 | SCN5A |
| MOL004885 | PPARG |
| MOL004885 | F10 |
| MOL004885 | PTGS2 |
| MOL004885 | CA2 |
| MOL004885 | F7 |
| MOL004885 | ACHE |
| MOL004885 | PTPN1 |
| MOL004885 | TOP2 |
| MOL004885 | ESR2 |
| MOL004885 | GSK3B |
| MOL004885 | HSP90 |
| MOL004885 | CDK2 |
| MOL004885 | CHEK1 |
| MOL004885 | PRSS1 |
| MOL004885 | PIM1 |
| MOL004885 | CCNA2 |
| MOL004885 | NCOA1 |
| MOL004885 | CALM1 |
| MOL004891 | NOS2 |
| MOL004891 | PTGS1 |
| MOL004891 | CHRM3 |
| MOL004891 | F2 |
| MOL004891 | KCNH2 |
| MOL004891 | CHRM1 |
| MOL004891 | ESR |
| MOL004891 | AR |
| MOL004891 | SCN5A |
| MOL004891 | PPARG |
| MOL004891 | PTGS2 |
| MOL004891 | HTR3A |
| MOL004891 | CA2 |
| MOL004891 | RXRA |
| MOL004891 | OPRD1 |
| MOL004891 | ACHE |
| MOL004891 | ADRA1B |
| MOL004891 | PTPN1 |
| MOL004891 | ADRB2 |
| MOL004891 | ADRA1D |
| MOL004891 | OPRM1 |
| MOL004891 | ESR2 |
| MOL004891 | DPP4 |
| MOL004891 | MAPK14 |
| MOL004891 | GSK3B |
| MOL004891 | CDK2 |
| MOL004891 | PIK3CG |
| MOL004891 | CHRNA7 |
| MOL004891 | CHEK1 |
| MOL004891 | PRKACA |
| MOL004891 | RXRB |
| MOL004891 | PRSS1 |
| MOL004891 | PIM1 |
| MOL004891 | CCNA2 |
| MOL004891 | NCOA1 |
| MOL004891 | CALM1 |
| MOL004898 | NOS2 |
| MOL004898 | F2 |
| MOL004898 | ESR |
| MOL004898 | AR |
| MOL004898 | PPARG |
| MOL004898 | PTGS2 |
| MOL004898 | PTPN1 |
| MOL004898 | ESR2 |
| MOL004898 | DPP4 |
| MOL004898 | MAPK14 |
| MOL004898 | GSK3B |
| MOL004898 | HSP90 |
| MOL004898 | CDK2 |
| MOL004898 | PRSS1 |
| MOL004898 | PIM1 |
| MOL004898 | CCNA2 |
| MOL004898 | NCOA2 |
| MOL004898 | CALM1 |
| MOL004903 | SOD1 |
| MOL004903 | NOS2 |
| MOL004903 | F2 |
| MOL004903 | ESR |
| MOL004903 | AR |
| MOL004903 | PPARG |
| MOL004903 | F10 |
| MOL004903 | PTGS2 |
| MOL004903 | CA2 |
| MOL004903 | F7 |
| MOL004903 | KDR |
| MOL004903 | DPP4 |
| MOL004903 | PIM1 |
| MOL004903 | CCNA2 |
| MOL004903 | CALM1 |
| MOL004904 | NOS2 |
| MOL004904 | F2 |
| MOL004904 | ESR |
| MOL004904 | AR |
| MOL004904 | PPARG |
| MOL004904 | F10 |
| MOL004904 | PTGS2 |
| MOL004904 | CA2 |
| MOL004904 | F7 |
| MOL004904 | KDR |
| MOL004904 | ACHE |
| MOL004904 | PTPN1 |
| MOL004904 | TOP2 |
| MOL004904 | CDK2 |
| MOL004904 | PRSS1 |
| MOL004904 | PIM1 |
| MOL004904 | CCNA2 |
| MOL004904 | CALM1 |
| MOL004907 | NOS2 |
| MOL004907 | PTGS1 |
| MOL004907 | ESR |
| MOL004907 | AR |
| MOL004907 | PPARG |
| MOL004907 | PTGS2 |
| MOL004907 | CA2 |
| MOL004907 | PTPN1 |
| MOL004907 | ESR2 |
| MOL004907 | DPP4 |
| MOL004907 | MAPK14 |
| MOL004907 | GSK3B |
| MOL004907 | HSP90 |
| MOL004907 | CDK2 |
| MOL004907 | PIK3CG |
| MOL004907 | CHEK1 |
| MOL004907 | PRKACA |
| MOL004907 | PRSS1 |
| MOL004907 | PIM1 |
| MOL004907 | CCNA2 |
| MOL004908 | NOS2 |
| MOL004908 | F2 |
| MOL004908 | CHRM1 |
| MOL004908 | ESR |
| MOL004908 | AR |
| MOL004908 | SCN5A |
| MOL004908 | PPARG |
| MOL004908 | PTGS2 |
| MOL004908 | CA2 |
| MOL004908 | RXRA |
| MOL004908 | ACHE |
| MOL004908 | ADRA1B |
| MOL004908 | PTPN1 |
| MOL004908 | ADRB2 |
| MOL004908 | ESR2 |
| MOL004908 | DPP4 |
| MOL004908 | MAPK14 |
| MOL004908 | GSK3B |
| MOL004908 | CDK2 |
| MOL004908 | CHEK1 |
| MOL004908 | PRKACA |
| MOL004908 | RXRB |
| MOL004908 | IGHG1 |
| MOL004908 | PRSS1 |
| MOL004908 | PIM1 |
| MOL004908 | CCNA2 |
| MOL004908 | NCOA2 |
| MOL004908 | NCOA1 |
| MOL004908 | CALM1 |
| MOL004910 | NOS2 |
| MOL004910 | PTGS1 |
| MOL004910 | F2 |
| MOL004910 | ESR |
| MOL004910 | AR |
| MOL004910 | SCN5A |
| MOL004910 | PPARG |
| MOL004910 | F10 |
| MOL004910 | PTGS2 |
| MOL004910 | NOS3 |
| MOL004910 | PDE3A |
| MOL004910 | PTPN1 |
| MOL004910 | ESR2 |
| MOL004910 | DPP4 |
| MOL004910 | MAPK14 |
| MOL004910 | GSK3B |
| MOL004910 | HSP90 |
| MOL004910 | CDK2 |
| MOL004910 | CHEK1 |
| MOL004910 | PRKACA |
| MOL004910 | PRSS1 |
| MOL004910 | PIM1 |
| MOL004910 | CCNA2 |
| MOL004910 | CALM1 |
| MOL004911 | NOS2 |
| MOL004911 | PTGS1 |
| MOL004911 | F2 |
| MOL004911 | ESR |
| MOL004911 | AR |
| MOL004911 | SCN5A |
| MOL004911 | PPARG |
| MOL004911 | F10 |
| MOL004911 | PTGS2 |
| MOL004911 | CA2 |
| MOL004911 | RXRA |
| MOL004911 | PTPN1 |
| MOL004911 | ADRB2 |
| MOL004911 | ESR2 |
| MOL004911 | DPP4 |
| MOL004911 | MAPK14 |
| MOL004911 | GSK3B |
| MOL004911 | HSP90 |
| MOL004911 | CDK2 |
| MOL004911 | CHEK1 |
| MOL004911 | PRSS1 |
| MOL004911 | PIM1 |
| MOL004911 | NCOA2 |
| MOL004911 | CALM1 |
| MOL004912 | NOS2 |
| MOL004912 | PTGS1 |
| MOL004912 | F2 |
| MOL004912 | ESR |
| MOL004912 | AR |
| MOL004912 | SCN5A |
| MOL004912 | PPARG |
| MOL004912 | F10 |
| MOL004912 | PTGS2 |
| MOL004912 | CA2 |
| MOL004912 | RXRA |
| MOL004912 | ACHE |
| MOL004912 | PTPN1 |
| MOL004912 | ESR2 |
| MOL004912 | DPP4 |
| MOL004912 | MAPK14 |
| MOL004912 | GSK3B |
| MOL004912 | CDK2 |
| MOL004912 | CHEK1 |
| MOL004912 | PRSS1 |
| MOL004912 | PIM1 |
| MOL004912 | CCNA2 |
| MOL004912 | CALM1 |
| MOL004913 | ESR |
| MOL004913 | PPARG |
| MOL004913 | PTPN1 |
| MOL004913 | ESR2 |
| MOL004913 | MAPK14 |
| MOL004913 | GSK3B |
| MOL004913 | HSP90 |
| MOL004913 | CDK2 |
| MOL004913 | CHEK1 |
| MOL004913 | PRKACA |
| MOL004913 | CCNA2 |
| MOL004914 | ESR |
| MOL004914 | AR |
| MOL004914 | PPARG |
| MOL004914 | PTPN1 |
| MOL004914 | ESR2 |
| MOL004914 | MAPK14 |
| MOL004914 | GSK3B |
| MOL004914 | HSP90 |
| MOL004914 | CDK2 |
| MOL004914 | CHEK1 |
| MOL004914 | PRKACA |
| MOL004914 | CCNA2 |
| MOL004915 | NOS2 |
| MOL004915 | F2 |
| MOL004915 | ESR |
| MOL004915 | AR |
| MOL004915 | SCN5A |
| MOL004915 | PPARG |
| MOL004915 | F10 |
| MOL004915 | PTGS2 |
| MOL004915 | PTPN1 |
| MOL004915 | ESR2 |
| MOL004915 | DPP4 |
| MOL004915 | MAPK14 |
| MOL004915 | GSK3B |
| MOL004915 | HSP90 |
| MOL004915 | CDK2 |
| MOL004915 | CHEK1 |
| MOL004915 | PRSS1 |
| MOL004915 | PIM1 |
| MOL004915 | CCNA2 |
| MOL004915 | CALM1 |
| MOL004924 | ESR |
| MOL004924 | AR |
| MOL004924 | PPARG |
| MOL004924 | PTGS2 |
| MOL004924 | CA2 |
| MOL004924 | ACHE |
| MOL004924 | CCNA2 |
| MOL004935 | NOS2 |
| MOL004935 | F2 |
| MOL004935 | ESR |
| MOL004935 | AR |
| MOL004935 | PPARG |
| MOL004935 | F10 |
| MOL004935 | PTGS2 |
| MOL004935 | KDR |
| MOL004935 | PTPN1 |
| MOL004935 | ESR2 |
| MOL004935 | DPP4 |
| MOL004935 | MAPK14 |
| MOL004935 | GSK3B |
| MOL004935 | HSP90 |
| MOL004935 | CDK2 |
| MOL004935 | PRSS1 |
| MOL004935 | PIM1 |
| MOL004935 | CCNA2 |
| MOL004935 | CALM1 |
| MOL004941 | NOS2 |
| MOL004941 | PTGS1 |
| MOL004941 | ESR |
| MOL004941 | AR |
| MOL004941 | PPARG |
| MOL004941 | PTGS2 |
| MOL004941 | CA2 |
| MOL004941 | RXRA |
| MOL004941 | PDE3A |
| MOL004941 | PTPN1 |
| MOL004941 | ADRB2 |
| MOL004941 | SLC6A4 |
| MOL004941 | ESR2 |
| MOL004941 | GABRA1 |
| MOL004941 | DPP4 |
| MOL004941 | MAPK14 |
| MOL004941 | GSK3B |
| MOL004941 | HSP90 |
| MOL004941 | CDK2 |
| MOL004941 | PIK3CG |
| MOL004941 | #N/A |
| MOL004941 | MAOB |
| MOL004941 | CHEK1 |
| MOL004941 | PRKACA |
| MOL004941 | PIM1 |
| MOL004941 | CCNA2 |
| MOL004941 | PKIA |
| MOL004941 | CALM1 |
| MOL004945 | NOS2 |
| MOL004945 | PTGS1 |
| MOL004945 | F2 |
| MOL004945 | ESR |
| MOL004945 | AR |
| MOL004945 | SCN5A |
| MOL004945 | PPARG |
| MOL004945 | F10 |
| MOL004945 | PTGS2 |
| MOL004945 | CA2 |
| MOL004945 | PDE3A |
| MOL004945 | ADRA1B |
| MOL004945 | PTPN1 |
| MOL004945 | ADRB2 |
| MOL004945 | ESR2 |
| MOL004945 | DPP4 |
| MOL004945 | MAPK14 |
| MOL004945 | GSK3B |
| MOL004945 | HSP90 |
| MOL004945 | CDK2 |
| MOL004945 | CHEK1 |
| MOL004945 | PRSS1 |
| MOL004945 | PIM1 |
| MOL004945 | CCNA2 |
| MOL004945 | CALM1 |
| MOL004948 | NOS2 |
| MOL004948 | ESR |
| MOL004948 | AR |
| MOL004948 | PTGS2 |
| MOL004948 | PTPN1 |
| MOL004948 | DPP4 |
| MOL004948 | GSK3B |
| MOL004948 | PRSS1 |
| MOL004948 | PIM1 |
| MOL004949 | NOS2 |
| MOL004949 | F2 |
| MOL004949 | ESR |
| MOL004949 | AR |
| MOL004949 | PPARG |
| MOL004949 | F10 |
| MOL004949 | PTGS2 |
| MOL004949 | CA2 |
| MOL004949 | PTPN1 |
| MOL004949 | ESR2 |
| MOL004949 | GSK3B |
| MOL004949 | HSP90 |
| MOL004949 | CDK2 |
| MOL004949 | PRSS1 |
| MOL004949 | PIM1 |
| MOL004949 | CCNA2 |
| MOL004949 | NCOA2 |
| MOL004949 | CALM1 |
| MOL004957 | NOS2 |
| MOL004957 | PTGS1 |
| MOL004957 | CHRM1 |
| MOL004957 | ESR |
| MOL004957 | AR |
| MOL004957 | SCN5A |
| MOL004957 | PPARG |
| MOL004957 | PTGS2 |
| MOL004957 | CA2 |
| MOL004957 | RXRA |
| MOL004957 | PDE3A |
| MOL004957 | PTPN1 |
| MOL004957 | SLC6A3 |
| MOL004957 | ADRB2 |
| MOL004957 | SLC6A4 |
| MOL004957 | ESR2 |
| MOL004957 | DPP4 |
| MOL004957 | MAPK14 |
| MOL004957 | GSK3B |
| MOL004957 | CDK2 |
| MOL004957 | MAOB |
| MOL004957 | CHEK1 |
| MOL004957 | PRKACA |
| MOL004957 | IGHG1 |
| MOL004957 | PRSS1 |
| MOL004957 | PIM1 |
| MOL004957 | CCNA2 |
| MOL004957 | PKIA |
| MOL004957 | CALM1 |
| MOL004959 | NOS2 |
| MOL004959 | PTGS1 |
| MOL004959 | F2 |
| MOL004959 | KCNH2 |
| MOL004959 | ESR |
| MOL004959 | AR |
| MOL004959 | SCN5A |
| MOL004959 | PPARG |
| MOL004959 | F10 |
| MOL004959 | PTGS2 |
| MOL004959 | NOS3 |
| MOL004959 | KDR |
| MOL004959 | RXRA |
| MOL004959 | ADRA1B |
| MOL004959 | ADRB2 |
| MOL004959 | ADRA1D |
| MOL004959 | TOP2 |
| MOL004959 | ESR2 |
| MOL004959 | DPP4 |
| MOL004959 | MAPK14 |
| MOL004959 | GSK3B |
| MOL004959 | HSP90 |
| MOL004959 | CDK2 |
| MOL004959 | PIK3CG |
| MOL004959 | CHEK1 |
| MOL004959 | PRSS1 |
| MOL004959 | PIM1 |
| MOL004959 | CCNA2 |
| MOL004959 | NCOA2 |
| MOL004959 | NCOA1 |
| MOL004959 | CALM1 |
| MOL004961 | NOS2 |
| MOL004961 | PTGS1 |
| MOL004961 | ESR |
| MOL004961 | AR |
| MOL004961 | SCN5A |
| MOL004961 | PPARG |
| MOL004961 | PTGS2 |
| MOL004961 | CA2 |
| MOL004961 | PTPN1 |
| MOL004961 | ESR2 |
| MOL004961 | DPP4 |
| MOL004961 | MAPK14 |
| MOL004961 | GSK3B |
| MOL004961 | HSP90 |
| MOL004961 | CDK2 |
| MOL004961 | PRSS1 |
| MOL004961 | PIM1 |
| MOL004961 | CCNA2 |
| MOL004961 | NCOA2 |
| MOL004961 | CALM1 |
| MOL004966 | NOS2 |
| MOL004966 | PTGS1 |
| MOL004966 | F2 |
| MOL004966 | KCNH2 |
| MOL004966 | ESR |
| MOL004966 | AR |
| MOL004966 | SCN5A |
| MOL004966 | PPARG |
| MOL004966 | F10 |
| MOL004966 | PTGS2 |
| MOL004966 | CA2 |
| MOL004966 | F7 |
| MOL004966 | KDR |
| MOL004966 | ADRA1B |
| MOL004966 | PTPN1 |
| MOL004966 | ADRB2 |
| MOL004966 | TOP2 |
| MOL004966 | ESR2 |
| MOL004966 | DPP4 |
| MOL004966 | MAPK14 |
| MOL004966 | GSK3B |
| MOL004966 | HSP90 |
| MOL004966 | CDK2 |
| MOL004966 | CHEK1 |
| MOL004966 | PRKACA |
| MOL004966 | PRSS1 |
| MOL004966 | PIM1 |
| MOL004966 | CCNA2 |
| MOL004966 | NCOA2 |
| MOL004966 | NCOA1 |
| MOL004966 | KCNMA1 |
| MOL004966 | CALM1 |
| MOL004974 | NOS2 |
| MOL004974 | PTGS1 |
| MOL004974 | F2 |
| MOL004974 | KCNH2 |
| MOL004974 | ESR |
| MOL004974 | AR |
| MOL004974 | SCN5A |
| MOL004974 | PPARG |
| MOL004974 | F10 |
| MOL004974 | PTGS2 |
| MOL004974 | CA2 |
| MOL004974 | F7 |
| MOL004974 | RXRA |
| MOL004974 | ACHE |
| MOL004974 | ADRA1B |
| MOL004974 | PTPN1 |
| MOL004974 | ADRB2 |
| MOL004974 | TOP2 |
| MOL004974 | ESR2 |
| MOL004974 | DPP4 |
| MOL004974 | MAPK14 |
| MOL004974 | GSK3B |
| MOL004974 | HSP90 |
| MOL004974 | CDK2 |
| MOL004974 | CHEK1 |
| MOL004974 | PRSS1 |
| MOL004974 | PIM1 |
| MOL004974 | CCNA2 |
| MOL004974 | NCOA2 |
| MOL004974 | NCOA1 |
| MOL004974 | KCNMA1 |
| MOL004974 | CALM1 |
| MOL004978 | NOS2 |
| MOL004978 | PTGS1 |
| MOL004978 | CHRM3 |
| MOL004978 | F2 |
| MOL004978 | KCNH2 |
| MOL004978 | CHRM1 |
| MOL004978 | ESR |
| MOL004978 | AR |
| MOL004978 | SCN5A |
| MOL004978 | PPARG |
| MOL004978 | F10 |
| MOL004978 | PTGS2 |
| MOL004978 | NOS3 |
| MOL004978 | CA2 |
| MOL004978 | RXRA |
| MOL004978 | ACHE |
| MOL004978 | ADRA1B |
| MOL004978 | PTPN1 |
| MOL004978 | SLC6A3 |
| MOL004978 | ADRB2 |
| MOL004978 | ESR2 |
| MOL004978 | DPP4 |
| MOL004978 | MAPK14 |
| MOL004978 | GSK3B |
| MOL004978 | CDK2 |
| MOL004978 | CHEK1 |
| MOL004978 | PRKACA |
| MOL004978 | RXRB |
| MOL004978 | PRSS1 |
| MOL004978 | PIM1 |
| MOL004978 | CCNA2 |
| MOL004978 | NCOA2 |
| MOL004978 | NCOA1 |
| MOL004978 | KCNMA1 |
| MOL004978 | CALM1 |
| MOL004980 | NOS2 |
| MOL004980 | PTGS1 |
| MOL004980 | F2 |
| MOL004980 | ESR |
| MOL004980 | AR |
| MOL004980 | SCN5A |
| MOL004980 | PPARG |
| MOL004980 | F10 |
| MOL004980 | PTGS2 |
| MOL004980 | CA2 |
| MOL004980 | PTPN1 |
| MOL004980 | ADRB2 |
| MOL004980 | ESR2 |
| MOL004980 | DPP4 |
| MOL004980 | MAPK14 |
| MOL004980 | GSK3B |
| MOL004980 | HSP90 |
| MOL004980 | CDK2 |
| MOL004980 | CHEK1 |
| MOL004980 | PRSS1 |
| MOL004980 | PIM1 |
| MOL004980 | CCNA2 |
| MOL004980 | NCOA2 |
| MOL004980 | CALM1 |
| MOL004985 | F2 |
| MOL004985 | PPARG |
| MOL004985 | NOS3 |
| MOL004985 | ACHE |
| MOL004985 | NCOA2 |
| MOL004988 | ESR |
| MOL004988 | AR |
| MOL004988 | F10 |
| MOL004988 | PTGS2 |
| MOL004988 | CA2 |
| MOL004988 | ESR2 |
| MOL004988 | PIM1 |
| MOL004988 | NCOA2 |
| MOL004988 | CALM1 |
| MOL004989 | NOS2 |
| MOL004989 | F2 |
| MOL004989 | ESR |
| MOL004989 | AR |
| MOL004989 | SCN5A |
| MOL004989 | PPARG |
| MOL004989 | F10 |
| MOL004989 | PTGS2 |
| MOL004989 | CA2 |
| MOL004989 | F7 |
| MOL004989 | PTPN1 |
| MOL004989 | ESR2 |
| MOL004989 | DPP4 |
| MOL004989 | MAPK14 |
| MOL004989 | GSK3B |
| MOL004989 | HSP90 |
| MOL004989 | CDK2 |
| MOL004989 | CHEK1 |
| MOL004989 | PRSS1 |
| MOL004989 | PIM1 |
| MOL004989 | CCNA2 |
| MOL004989 | CALM1 |
| MOL004990 | NOS2 |
| MOL004990 | PTGS1 |
| MOL004990 | ESR |
| MOL004990 | AR |
| MOL004990 | PPARG |
| MOL004990 | PTGS2 |
| MOL004990 | CA2 |
| MOL004990 | PTPN1 |
| MOL004990 | ESR2 |
| MOL004990 | DPP4 |
| MOL004990 | MAPK14 |
| MOL004990 | GSK3B |
| MOL004990 | HSP90 |
| MOL004990 | CDK2 |
| MOL004990 | CHEK1 |
| MOL004990 | PRKACA |
| MOL004990 | PIM1 |
| MOL004990 | CCNA2 |
| MOL004991 | NOS2 |
| MOL004991 | PTGS1 |
| MOL004991 | F2 |
| MOL004991 | ESR |
| MOL004991 | AR |
| MOL004991 | SCN5A |
| MOL004991 | PPARG |
| MOL004991 | PTGS2 |
| MOL004991 | NOS3 |
| MOL004991 | CA2 |
| MOL004991 | RXRA |
| MOL004991 | ACHE |
| MOL004991 | PDE3A |
| MOL004991 | ADRA1B |
| MOL004991 | PTPN1 |
| MOL004991 | ADRB2 |
| MOL004991 | ADRA1D |
| MOL004991 | ESR2 |
| MOL004991 | GABRA1 |
| MOL004991 | DPP4 |
| MOL004991 | MAPK14 |
| MOL004991 | GSK3B |
| MOL004991 | HSP90 |
| MOL004991 | CDK2 |
| MOL004991 | CHEK1 |
| MOL004991 | PRSS1 |
| MOL004991 | PIM1 |
| MOL004991 | CCNA2 |
| MOL004991 | NCOA2 |
| MOL004991 | CALM1 |
| MOL004993 | NOS2 |
| MOL004993 | F2 |
| MOL004993 | ESR |
| MOL004993 | AR |
| MOL004993 | SCN5A |
| MOL004993 | PPARG |
| MOL004993 | F10 |
| MOL004993 | PTGS2 |
| MOL004993 | F7 |
| MOL004993 | PTPN1 |
| MOL004993 | ESR2 |
| MOL004993 | DPP4 |
| MOL004993 | MAPK14 |
| MOL004993 | GSK3B |
| MOL004993 | HSP90 |
| MOL004993 | CDK2 |
| MOL004993 | CHEK1 |
| MOL004993 | PRSS1 |
| MOL004993 | PIM1 |
| MOL004993 | NCOA1 |
| MOL004993 | CALM1 |
| MOL004996 | F2 |
| MOL004996 | PPARG |
| MOL004996 | NOS3 |
| MOL004996 | ACHE |
| MOL004996 | NCOA2 |
| MOL005000 | NOS2 |
| MOL005000 | F2 |
| MOL005000 | ESR |
| MOL005000 | AR |
| MOL005000 | PPARG |
| MOL005000 | F10 |
| MOL005000 | PTGS2 |
| MOL005000 | NOS3 |
| MOL005000 | CA2 |
| MOL005000 | PTPN1 |
| MOL005000 | TOP2 |
| MOL005000 | ESR2 |
| MOL005000 | DPP4 |
| MOL005000 | MAPK14 |
| MOL005000 | GSK3B |
| MOL005000 | HSP90 |
| MOL005000 | CHEK1 |
| MOL005000 | PRSS1 |
| MOL005000 | PIM1 |
| MOL005000 | CCNA2 |
| MOL005000 | NCOA2 |
| MOL005000 | CALM1 |
| MOL005001 | ESR |
| MOL005001 | AR |
| MOL005001 | F10 |
| MOL005001 | PTGS2 |
| MOL005001 | CA2 |
| MOL005001 | KDR |
| MOL005001 | PTPN1 |
| MOL005001 | TOP2 |
| MOL005001 | HSP90 |
| MOL005001 | PRSS1 |
| MOL005001 | PIM1 |
| MOL005001 | CCNA2 |
| MOL005001 | NCOA2 |
| MOL005001 | CALM1 |
| MOL005003 | NOS2 |
| MOL005003 | PTGS1 |
| MOL005003 | CHRM3 |
| MOL005003 | F2 |
| MOL005003 | KCNH2 |
| MOL005003 | CHRM1 |
| MOL005003 | ESR |
| MOL005003 | AR |
| MOL005003 | SCN5A |
| MOL005003 | PPARG |
| MOL005003 | F10 |
| MOL005003 | CHRM5 |
| MOL005003 | PTGS2 |
| MOL005003 | NOS3 |
| MOL005003 | CA2 |
| MOL005003 | RXRA |
| MOL005003 | ACHE |
| MOL005003 | ADRA1B |
| MOL005003 | ADRB2 |
| MOL005003 | ESR2 |
| MOL005003 | DPP4 |
| MOL005003 | MAPK14 |
| MOL005003 | GSK3B |
| MOL005003 | HSP90 |
| MOL005003 | CDK2 |
| MOL005003 | CHEK1 |
| MOL005003 | RXRB |
| MOL005003 | PRSS1 |
| MOL005003 | PIM1 |
| MOL005003 | CCNA2 |
| MOL005003 | NCOA2 |
| MOL005003 | CALM1 |
| MOL005007 | NOS2 |
| MOL005007 | PTGS1 |
| MOL005007 | F2 |
| MOL005007 | KCNH2 |
| MOL005007 | ESR |
| MOL005007 | AR |
| MOL005007 | SCN5A |
| MOL005007 | PPARG |
| MOL005007 | F10 |
| MOL005007 | PTGS2 |
| MOL005007 | CA2 |
| MOL005007 | F7 |
| MOL005007 | KDR |
| MOL005007 | ACHE |
| MOL005007 | PTPN1 |
| MOL005007 | TOP2 |
| MOL005007 | ESR2 |
| MOL005007 | DPP4 |
| MOL005007 | PPARD |
| MOL005007 | GSK3B |
| MOL005007 | HSP90 |
| MOL005007 | CDK2 |
| MOL005007 | CHEK1 |
| MOL005007 | PRKACA |
| MOL005007 | PRSS1 |
| MOL005007 | PIM1 |
| MOL005007 | CCNA2 |
| MOL005007 | NCOA2 |
| MOL005007 | NCOA1 |
| MOL005007 | KCNMA1 |
| MOL005007 | CALM1 |
| MOL005008 | NOS2 |
| MOL005008 | ESR |
| MOL005008 | AR |
| MOL005008 | F10 |
| MOL005008 | PTGS2 |
| MOL005008 | CA2 |
| MOL005008 | F7 |
| MOL005008 | ACHE |
| MOL005008 | PTPN1 |
| MOL005008 | TOP2 |
| MOL005008 | ESR2 |
| MOL005008 | DPP4 |
| MOL005008 | GSK3B |
| MOL005008 | HSP90 |
| MOL005008 | CDK2 |
| MOL005008 | PRSS1 |
| MOL005008 | PIM1 |
| MOL005008 | CCNA2 |
| MOL005008 | CALM1 |
| MOL005012 | NOS2 |
| MOL005012 | F2 |
| MOL005012 | ESR |
| MOL005012 | AR |
| MOL005012 | SCN5A |
| MOL005012 | PPARG |
| MOL005012 | F10 |
| MOL005012 | PTGS2 |
| MOL005012 | CA2 |
| MOL005012 | PTPN1 |
| MOL005012 | ESR2 |
| MOL005012 | DPP4 |
| MOL005012 | MAPK14 |
| MOL005012 | GSK3B |
| MOL005012 | CDK2 |
| MOL005012 | CHEK1 |
| MOL005012 | PRSS1 |
| MOL005012 | PIM1 |
| MOL005012 | CCNA2 |
| MOL005012 | CALM1 |
| MOL005013 | AR |
| MOL005013 | NR3C1 |
| MOL005016 | NOS2 |
| MOL005016 | PTGS1 |
| MOL005016 | ESR |
| MOL005016 | AR |
| MOL005016 | SCN5A |
| MOL005016 | PPARG |
| MOL005016 | PTGS2 |
| MOL005016 | CA2 |
| MOL005016 | RXRA |
| MOL005016 | PTPN1 |
| MOL005016 | ESR2 |
| MOL005016 | DPP4 |
| MOL005016 | MAPK14 |
| MOL005016 | GSK3B |
| MOL005016 | HSP90 |
| MOL005016 | CDK2 |
| MOL005016 | CHEK1 |
| MOL005016 | PRSS1 |
| MOL005016 | PIM1 |
| MOL005016 | CCNA2 |
| MOL005016 | NCOA2 |
| MOL005016 | CALM1 |
| MOL005017 | NOS2 |
| MOL005017 | F2 |
| MOL005017 | ESR |
| MOL005017 | AR |
| MOL005017 | PPARG |
| MOL005017 | PTGS2 |
| MOL005017 | KDR |
| MOL005017 | PTPN1 |
| MOL005017 | ESR2 |
| MOL005017 | MAPK14 |
| MOL005017 | GSK3B |
| MOL005017 | HSP90 |
| MOL005017 | CDK2 |
| MOL005017 | CHEK1 |
| MOL005017 | PRKACA |
| MOL005017 | PIM1 |
| MOL005017 | CCNA2 |
| MOL005018 | NOS2 |
| MOL005018 | F2 |
| MOL005018 | ESR |
| MOL005018 | AR |
| MOL005018 | F10 |
| MOL005018 | PTGS2 |
| MOL005018 | CA2 |
| MOL005018 | ESR2 |
| MOL005018 | GSK3B |
| MOL005018 | PIM1 |
| MOL005018 | NCOA2 |
| MOL005018 | CALM1 |
| MOL005020 | NOS2 |
| MOL005020 | F2 |
| MOL005020 | ESR |
| MOL005020 | AR |
| MOL005020 | SCN5A |
| MOL005020 | PPARG |
| MOL005020 | F10 |
| MOL005020 | PTGS2 |
| MOL005020 | CA2 |
| MOL005020 | PTPN1 |
| MOL005020 | ADRB2 |
| MOL005020 | ESR2 |
| MOL005020 | DPP4 |
| MOL005020 | MAPK14 |
| MOL005020 | HSP90 |
| MOL005020 | CDK2 |
| MOL005020 | CHEK1 |
| MOL005020 | PRSS1 |
| MOL005020 | PIM1 |
| MOL005020 | CCNA2 |
| MOL005020 | NCOA2 |
| MOL005020 | CALM1 |
| MOL000098 | NOS2 |
| MOL000098 | PTGS1 |
| MOL000098 | INSR |
| MOL000098 | ESR |
| MOL000098 | AR |
| MOL000098 | BCL2 |
| MOL000098 | ALOX5 |
| MOL000098 | PTGS2 |
| MOL000098 | ODC1 |
| MOL000098 | ACACA |
| MOL000098 | MMP2 |
| MOL000098 | TNF |
| MOL000098 | EGFR |
| MOL000098 | ESR2 |
| MOL000098 | MGAM |
| MOL000098 | PLAU |
| MOL000098 | IL6 |
| MOL000098 | MMP1 |
| MOL000098 | MAPK1 |
| MOL000098 | PON1 |
| MOL000098 | CTSD |
| MOL000098 | IFNG |
| MOL000098 | JUN |
| MOL000098 | CCL2 |
| MOL000098 | IL1B |
| MOL000098 | GSK3B |
| MOL000098 | SELE |
| MOL000098 | MPO |
| MOL000098 | CDK1 |
| MOL000098 | PLAT |
| MOL000098 | GJA1 |
| MOL000098 | VCAM1 |
| MOL000098 | THBD |
| MOL000098 | F3 |
| MOL000098 | NQO1 |
| MOL000098 | XDH |
| MOL000098 | PTGER3 |
| MOL000098 | SOD1 |
| MOL000098 | CYP3A4 |
| MOL000098 | TP53 |
| MOL000098 | CYP1A2 |
| MOL000098 | COL1A1 |
| MOL000098 | GSTP1 |
| MOL000098 | EGF |
| MOL000098 | VEGFA |
| MOL000098 | POR |
| MOL000098 | SULT1E1 |
| MOL000098 | TOP1 |
| MOL000098 | HMOX1 |
| MOL000098 | COL3A1 |
| MOL000098 | RB1 |
| MOL000098 | GSTM1 |
| MOL000098 | HSPA5 |
| MOL000098 | ACPP |
| MOL000098 | AHR |
| MOL000098 | GSTM2 |
| MOL000098 | IL2 |
| MOL000098 | F2 |
| MOL000098 | KCNH2 |
| MOL000098 | SCN5A |
| MOL000098 | PPARG |
| MOL000098 | F10 |
| MOL000098 | NOS3 |
| MOL000098 | CA2 |
| MOL000098 | F7 |
| MOL000098 | RXRA |
| MOL000098 | ACHE |
| MOL000098 | PTPN1 |
| MOL000098 | ADRB2 |
| MOL000098 | TOP2 |
| MOL000098 | AKR1B1 |
| MOL000098 | GABRA1 |
| MOL000098 | DPP4 |
| MOL000098 | MAPK14 |
| MOL000098 | MMP3 |
| MOL000098 | HSP90 |
| MOL000098 | CDK2 |
| MOL000098 | PIK3CG |
| MOL000098 | MAOB |
| MOL000098 | CHEK1 |
| MOL000098 | PRKACA |
| MOL000098 | PRSS1 |
| MOL000098 | PIM1 |
| MOL000098 | CCNA2 |
| MOL000098 | NCOA2 |
| MOL000354 | NOS2 |
| MOL000354 | XDH |
| MOL000354 | PTGS1 |
| MOL000354 | F2 |
| MOL000354 | ESR |
| MOL000354 | AR |
| MOL000354 | PPARG |
| MOL000354 | PTGS2 |
| MOL000354 | NOS3 |
| MOL000354 | CA2 |
| MOL000354 | F7 |
| MOL000354 | ACHE |
| MOL000354 | PTPN1 |
| MOL000354 | AKR1B1 |
| MOL000354 | ESR2 |
| MOL000354 | GABRA1 |
| MOL000354 | DPP4 |
| MOL000354 | PYGM |
| MOL000354 | PPARD |
| MOL000354 | MAPK14 |
| MOL000354 | GSK3B |
| MOL000354 | HSP90 |
| MOL000354 | CDK2 |
| MOL000354 | PIK3CG |
| MOL000354 | MAOB |
| MOL000354 | CHEK1 |
| MOL000354 | PRKACA |
| MOL000354 | PRSS1 |
| MOL000354 | PIM1 |
| MOL000354 | CCNA2 |
| MOL000354 | GRIA2 |
| MOL000354 | NCOA2 |
| MOL000354 | NCOA1 |
| MOL000354 | CALM1 |
| MOL000422 | NOS2 |
| MOL000422 | INSR |
| MOL000422 | ESR |
| MOL000422 | BCL2 |
| MOL000422 | ALOX5 |
| MOL000422 | PTGS2 |
| MOL000422 | AKR1C3 |
| MOL000422 | TNF |
| MOL000422 | ESR2 |
| MOL000422 | MMP1 |
| MOL000422 | JUN |
| MOL000422 | SELE |
| MOL000422 | CDK1 |
| MOL000422 | VCAM1 |
| MOL000422 | XDH |
| MOL000422 | CYP3A4 |
| MOL000422 | MAPK8 |
| MOL000422 | CYP1A2 |
| MOL000422 | GSTP1 |
| MOL000422 | HMOX1 |
| MOL000422 | GSTM1 |
| MOL000422 | AHR |
| MOL000422 | GSTM2 |
| MOL000422 | PPP3CA |
| MOL000422 | PTGS1 |
| MOL000422 | F2 |
| MOL000422 | CHRM1 |
| MOL000422 | AR |
| MOL000422 | PPARG |
| MOL000422 | NOS3 |
| MOL000422 | CA2 |
| MOL000422 | F7 |
| MOL000422 | GABRA2 |
| MOL000422 | ACHE |
| MOL000422 | SLC6A2 |
| MOL000422 | PGR |
| MOL000422 | CHRM2 |
| MOL000422 | ADRA1B |
| MOL000422 | PTPN1 |
| MOL000422 | TOP2 |
| MOL000422 | GABRA1 |
| MOL000422 | DPP4 |
| MOL000422 | MAPK14 |
| MOL000422 | GSK3B |
| MOL000422 | HSP90 |
| MOL000422 | CDK2 |
| MOL000422 | PIK3CG |
| MOL000422 | CHEK1 |
| MOL000422 | PRKACA |
| MOL000422 | PRSS1 |
| MOL000422 | PIM1 |
| MOL000422 | CCNA2 |
| MOL000422 | NCOA2 |
| MOL000422 | CALM1 |
| MOL001418 | F2 |
| MOL001418 | CHRM1 |
| MOL001418 | ESR |
| MOL001418 | ADRB1 |
| MOL001418 | PTGS2 |
| MOL001418 | ADRA2A |
| MOL001418 | CA2 |
| MOL001418 | RXRA |
| MOL001418 | ACHE |
| MOL001418 | SLC6A2 |
| MOL001418 | CHRM2 |
| MOL001418 | ADRA2B |
| MOL001418 | SLC6A3 |
| MOL001418 | ADRB2 |
| MOL001418 | SLC6A4 |
| MOL001418 | MAOB |
| MOL001420 | ESR |
| MOL001420 | AR |
| MOL001420 | PTGS2 |
| MOL001420 | ACHE |
| MOL001420 | PGR |
| MOL001420 | NR3C1 |
| MOL001420 | GABRA1 |
| MOL001420 | DPP4 |
| MOL001420 | ADH1C |
| MOL001420 | PRSS1 |
| MOL001421 | NOS2 |
| MOL001421 | CHRM3 |
| MOL001421 | F2 |
| MOL001421 | CHRM1 |
| MOL001421 | ESR |
| MOL001421 | AR |
| MOL001421 | PPARG |
| MOL001421 | F10 |
| MOL001421 | PTGS2 |
| MOL001421 | NOS3 |
| MOL001421 | CA2 |
| MOL001421 | PDE3A |
| MOL001421 | SLC6A2 |
| MOL001421 | ADRA1B |
| MOL001421 | ADRB2 |
| MOL001421 | SLC6A4 |
| MOL001421 | DPP4 |
| MOL001421 | MAPK14 |
| MOL001421 | GSK3B |
| MOL001421 | HSP90 |
| MOL001421 | CDK2 |
| MOL001421 | CHEK1 |
| MOL001421 | PRKACA |
| MOL001421 | PRSS1 |
| MOL001421 | PIM1 |
| MOL001421 | CCNA2 |
| MOL001421 | CALM1 |
| MOL001422 | NOS2 |
| MOL001422 | PTGS1 |
| MOL001422 | F2 |
| MOL001422 | CHRM1 |
| MOL001422 | PTGS2 |
| MOL001422 | NOS3 |
| MOL001422 | RXRA |
| MOL001422 | ACHE |
| MOL001422 | SLC6A2 |
| MOL001422 | GABRA1 |
| MOL001422 | DPP4 |
| MOL001422 | PRSS1 |
| MOL001422 | GRIA2 |
| MOL001439 | NOS2 |
| MOL001439 | ALDH2 |
| MOL001439 | ALOX5 |
| MOL001439 | PTGS2 |
| MOL001439 | PLA2G4A |
| MOL001439 | MAPK1 |
| MOL001439 | TNFRSF1B |
| MOL001439 | SELP |
| MOL001439 | CDK2 |
| MOL001439 | CDK4 |
| MOL001439 | EGF |
| MOL001439 | ABCG1 |
| MOL001439 | ALDH3A1 |
| MOL001439 | TNFRSF1A |
| MOL001439 | GLB1 |
| MOL001439 | G6PD |
| MOL001439 | C1R |
| MOL001439 | COL1A2 |
| MOL001439 | KCNK2 |
| MOL001439 | PTGS1 |
| MOL001439 | F2 |
| MOL001439 | ESR |
| MOL001439 | PPARG |
| MOL001439 | NOS3 |
| MOL001439 | RXRA |
| MOL001439 | ACHE |
| MOL001439 | SLC6A2 |
| MOL001439 | RXRG |
| MOL001439 | DPP4 |
| MOL001439 | TRPV1 |
| MOL000359 | ESR |
| MOL000359 | AR |
| MOL000359 | PGR |
| MOL000359 | NR3C2 |
| MOL000359 | NR3C1 |
| MOL000359 | NCOA2 |
| MOL000422 | NOS2 |
| MOL000422 | INSR |
| MOL000422 | ESR |
| MOL000422 | BCL2 |
| MOL000422 | ALOX5 |
| MOL000422 | PTGS2 |
| MOL000422 | AKR1C3 |
| MOL000422 | TNF |
| MOL000422 | ESR2 |
| MOL000422 | MMP1 |
| MOL000422 | JUN |
| MOL000422 | SELE |
| MOL000422 | CDK1 |
| MOL000422 | VCAM1 |
| MOL000422 | XDH |
| MOL000422 | CYP3A4 |
| MOL000422 | MAPK8 |
| MOL000422 | CYP1A2 |
| MOL000422 | GSTP1 |
| MOL000422 | HMOX1 |
| MOL000422 | GSTM1 |
| MOL000422 | AHR |
| MOL000422 | GSTM2 |
| MOL000422 | PPP3CA |
| MOL000422 | PTGS1 |
| MOL000422 | F2 |
| MOL000422 | CHRM1 |
| MOL000422 | AR |
| MOL000422 | PPARG |
| MOL000422 | NOS3 |
| MOL000422 | CA2 |
| MOL000422 | F7 |
| MOL000422 | GABRA2 |
| MOL000422 | ACHE |
| MOL000422 | SLC6A2 |
| MOL000422 | PGR |
| MOL000422 | CHRM2 |
| MOL000422 | ADRA1B |
| MOL000422 | PTPN1 |
| MOL000422 | TOP2 |
| MOL000422 | GABRA1 |
| MOL000422 | DPP4 |
| MOL000422 | MAPK14 |
| MOL000422 | GSK3B |
| MOL000422 | HSP90 |
| MOL000422 | CDK2 |
| MOL000422 | PIK3CG |
| MOL000422 | CHEK1 |
| MOL000422 | PRKACA |
| MOL000422 | PRSS1 |
| MOL000422 | PIM1 |
| MOL000422 | CCNA2 |
| MOL000422 | NCOA2 |
| MOL000422 | CALM1 |
| MOL001558 | NOS2 |
| MOL001558 | ACACA |
| MOL001558 | FASN |
| MOL001558 | CYP2B6 |
| MOL001558 | ECE1 |
| MOL001558 | G6PD |
| MOL001558 | ACADM |
| MOL001558 | ECI1 |
| MOL001558 | ACOX1 |
| MOL001558 | DECR1 |
| MOL001558 | EHHADH |
| MOL001558 | ESR |
| MOL001558 | AR |
| MOL001558 | SCN5A |
| MOL001558 | F10 |
| MOL001558 | PTGS2 |
| MOL001558 | ACHE |
| MOL001558 | PTPN1 |
| MOL001558 | ESR2 |
| MOL001558 | DPP4 |
| MOL001558 | MAPK14 |
| MOL001558 | GSK3B |
| MOL001558 | PRSS1 |
| MOL001558 | PIM1 |
| MOL002322 | NOS2 |
| MOL002322 | PTGS2 |
| MOL002322 | TNF |
| MOL002322 | AR |
| MOL002322 | CA2 |
| MOL002322 | PTPN1 |
| MOL002322 | TOP2 |
| MOL002322 | PIM1 |
| MOL003137 | CA2 |
| MOL003137 | PTPN1 |
| MOL003137 | TOP2 |
| MOL003137 | GSK3B |
| MOL003137 | PIM1 |
| MOL003152 | NOS2 |
| MOL003152 | PTGS1 |
| MOL003152 | ESR |
| MOL003152 | AR |
| MOL003152 | PPARG |
| MOL003152 | PTGS2 |
| MOL003152 | CA2 |
| MOL003152 | PTPN1 |
| MOL003152 | AKR1B1 |
| MOL003152 | ESR2 |
| MOL003152 | DPP4 |
| MOL003152 | MAPK14 |
| MOL003152 | GSK3B |
| MOL003152 | HSP90 |
| MOL003152 | CDK2 |
| MOL003152 | PIK3CG |
| MOL003152 | CHEK1 |
| MOL003152 | PRKACA |
| MOL003152 | PIM1 |
| MOL003152 | CCNA2 |
| MOL003152 | PKIA |
| MOL003155 | NOS2 |
| MOL003155 | PTGS1 |
| MOL003155 | CHRM3 |
| MOL003155 | F2 |
| MOL003155 | CHRM1 |
| MOL003155 | ESR |
| MOL003155 | AR |
| MOL003155 | SCN5A |
| MOL003155 | PTGS2 |
| MOL003155 | CHRM4 |
| MOL003155 | RXRA |
| MOL003155 | ADRB2 |
| MOL003155 | OPRM1 |
| MOL003155 | GABRA1 |
| MOL003155 | DPP4 |
| MOL003155 | GSK3B |
| MOL003155 | CDK2 |
| MOL003170 | NOS2 |
| MOL003170 | PTGS1 |
| MOL003170 | ESR |
| MOL003170 | PPARG |
| MOL003170 | PTGS2 |
| MOL003170 | CA2 |
| MOL003170 | PDE3A |
| MOL003170 | PTPN1 |
| MOL003170 | ESR2 |
| MOL003170 | MAPK14 |
| MOL003170 | HSP90 |
| MOL003170 | PRKACA |
| MOL003170 | PIM1 |
| MOL000006 | NOS2 |
| MOL000006 | INSR |
| MOL000006 | AR |
| MOL000006 | PTGS2 |
| MOL000006 | MMP2 |
| MOL000006 | TNF |
| MOL000006 | EGFR |
| MOL000006 | MET |
| MOL000006 | IL6 |
| MOL000006 | MMP1 |
| MOL000006 | MAPK1 |
| MOL000006 | IFNG |
| MOL000006 | JUN |
| MOL000006 | CDK2 |
| MOL000006 | XDH |
| MOL000006 | CDK4 |
| MOL000006 | TP53 |
| MOL000006 | APP |
| MOL000006 | GSTP1 |
| MOL000006 | VEGFA |
| MOL000006 | TOP1 |
| MOL000006 | HMOX1 |
| MOL000006 | RB1 |
| MOL000006 | CASP7 |
| MOL000006 | IL2 |
| MOL000006 | PTGS1 |
| MOL000006 | ESR |
| MOL000006 | PPARG |
| MOL000006 | CA2 |
| MOL000006 | PTPN1 |
| MOL000006 | ESR2 |
| MOL000006 | DPP4 |
| MOL000006 | MAPK14 |
| MOL000006 | GSK3B |
| MOL000006 | HSP90 |
| MOL000006 | PIK3CG |
| MOL000006 | PRKACA |
| MOL000006 | PRSS1 |
| MOL000006 | PIM1 |
| MOL000006 | CCNA2 |
| MOL000006 | NCOA2 |
| MOL000098 | NOS2 |
| MOL000098 | PTGS1 |
| MOL000098 | INSR |
| MOL000098 | ESR |
| MOL000098 | AR |
| MOL000098 | BCL2 |
| MOL000098 | ALOX5 |
| MOL000098 | PTGS2 |
| MOL000098 | ODC1 |
| MOL000098 | ACACA |
| MOL000098 | MMP2 |
| MOL000098 | TNF |
| MOL000098 | EGFR |
| MOL000098 | ESR2 |
| MOL000098 | MGAM |
| MOL000098 | PLAU |
| MOL000098 | IL6 |
| MOL000098 | MMP1 |
| MOL000098 | MAPK1 |
| MOL000098 | PON1 |
| MOL000098 | CTSD |
| MOL000098 | IFNG |
| MOL000098 | JUN |
| MOL000098 | CCL2 |
| MOL000098 | IL1B |
| MOL000098 | GSK3B |
| MOL000098 | SELE |
| MOL000098 | MPO |
| MOL000098 | CDK1 |
| MOL000098 | PLAT |
| MOL000098 | GJA1 |
| MOL000098 | VCAM1 |
| MOL000098 | THBD |
| MOL000098 | F3 |
| MOL000098 | NQO1 |
| MOL000098 | XDH |
| MOL000098 | PTGER3 |
| MOL000098 | SOD1 |
| MOL000098 | CYP3A4 |
| MOL000098 | TP53 |
| MOL000098 | CYP1A2 |
| MOL000098 | COL1A1 |
| MOL000098 | GSTP1 |
| MOL000098 | EGF |
| MOL000098 | VEGFA |
| MOL000098 | POR |
| MOL000098 | SULT1E1 |
| MOL000098 | TOP1 |
| MOL000098 | HMOX1 |
| MOL000098 | COL3A1 |
| MOL000098 | RB1 |
| MOL000098 | GSTM1 |
| MOL000098 | HSPA5 |
| MOL000098 | ACPP |
| MOL000098 | AHR |
| MOL000098 | GSTM2 |
| MOL000098 | IL2 |
| MOL000098 | F2 |
| MOL000098 | KCNH2 |
| MOL000098 | SCN5A |
| MOL000098 | PPARG |
| MOL000098 | F10 |
| MOL000098 | NOS3 |
| MOL000098 | CA2 |
| MOL000098 | F7 |
| MOL000098 | RXRA |
| MOL000098 | ACHE |
| MOL000098 | PTPN1 |
| MOL000098 | ADRB2 |
| MOL000098 | TOP2 |
| MOL000098 | AKR1B1 |
| MOL000098 | GABRA1 |
| MOL000098 | DPP4 |
| MOL000098 | MAPK14 |
| MOL000098 | MMP3 |
| MOL000098 | HSP90 |
| MOL000098 | CDK2 |
| MOL000098 | PIK3CG |
| MOL000098 | MAOB |
| MOL000098 | CHEK1 |
| MOL000098 | PRKACA |
| MOL000098 | PRSS1 |
| MOL000098 | PIM1 |
| MOL000098 | CCNA2 |
| MOL000098 | NCOA2 |
| MOL000358 | BCL2 |
| MOL000358 | PON1 |
| MOL000358 | JUN |
| MOL000358 | MAP2 |
| MOL000358 | NOS2 |
| MOL000358 | PTGS1 |
| MOL000358 | DRD1 |
| MOL000358 | CHRM3 |
| MOL000358 | F2 |
| MOL000358 | KCNH2 |
| MOL000358 | CHRM1 |
| MOL000358 | ESR |
| MOL000358 | AR |
| MOL000358 | SCN5A |
| MOL000358 | PPARG |
| MOL000358 | PTGS2 |
| MOL000358 | CA2 |
| MOL000358 | GABRA2 |
| MOL000358 | CHRM4 |
| MOL000358 | ACHE |
| MOL000358 | PDE3A |
| MOL000358 | HTR2A |
| MOL000358 | GABRA5 |
| MOL000358 | ADRA1A |
| MOL000358 | GABRA3 |
| MOL000358 | PGR |
| MOL000358 | CHRM2 |
| MOL000358 | ADRA1B |
| MOL000358 | PTPN1 |
| MOL000358 | ADRB2 |
| MOL000358 | CHRNA2 |
| MOL000358 | SLC6A4 |
| MOL000358 | OPRM1 |
| MOL000358 | ESR2 |
| MOL000358 | NR3C1 |
| MOL000358 | GABRA1 |
| MOL000358 | DPP4 |
| MOL000358 | MAPK14 |
| MOL000358 | GSK3B |
| MOL000358 | HSP90 |
| MOL000358 | CDK2 |
| MOL000358 | PIK3CG |
| MOL000358 | CHRNA7 |
| MOL000358 | CHEK1 |
| MOL000358 | PRKACA |
| MOL000358 | PRSS1 |
| MOL000358 | PIM1 |
| MOL000358 | CCNA2 |
| MOL000358 | NCOA2 |
| MOL000422 | NOS2 |
| MOL000422 | INSR |
| MOL000422 | ESR |
| MOL000422 | BCL2 |
| MOL000422 | ALOX5 |
| MOL000422 | PTGS2 |
| MOL000422 | AKR1C3 |
| MOL000422 | TNF |
| MOL000422 | ESR2 |
| MOL000422 | MMP1 |
| MOL000422 | JUN |
| MOL000422 | SELE |
| MOL000422 | CDK1 |
| MOL000422 | VCAM1 |
| MOL000422 | XDH |
| MOL000422 | CYP3A4 |
| MOL000422 | MAPK8 |
| MOL000422 | CYP1A2 |
| MOL000422 | GSTP1 |
| MOL000422 | HMOX1 |
| MOL000422 | GSTM1 |
| MOL000422 | AHR |
| MOL000422 | GSTM2 |
| MOL000422 | PPP3CA |
| MOL000422 | PTGS1 |
| MOL000422 | F2 |
| MOL000422 | CHRM1 |
| MOL000422 | AR |
| MOL000422 | PPARG |
| MOL000422 | NOS3 |
| MOL000422 | CA2 |
| MOL000422 | F7 |
| MOL000422 | GABRA2 |
| MOL000422 | ACHE |
| MOL000422 | SLC6A2 |
| MOL000422 | PGR |
| MOL000422 | CHRM2 |
| MOL000422 | ADRA1B |
| MOL000422 | PTPN1 |
| MOL000422 | TOP2 |
| MOL000422 | GABRA1 |
| MOL000422 | DPP4 |
| MOL000422 | MAPK14 |
| MOL000422 | GSK3B |
| MOL000422 | HSP90 |
| MOL000422 | CDK2 |
| MOL000422 | PIK3CG |
| MOL000422 | CHEK1 |
| MOL000422 | PRKACA |
| MOL000422 | PRSS1 |
| MOL000422 | PIM1 |
| MOL000422 | CCNA2 |
| MOL000422 | NCOA2 |
| MOL000422 | CALM1 |
| MOL000449 | NOS2 |
| MOL000449 | PTGS1 |
| MOL000449 | CHRM3 |
| MOL000449 | F2 |
| MOL000449 | CHRM1 |
| MOL000449 | ESR |
| MOL000449 | AR |
| MOL000449 | ADRB1 |
| MOL000449 | SCN5A |
| MOL000449 | PPARG |
| MOL000449 | PTGS2 |
| MOL000449 | NOS3 |
| MOL000449 | ADRA2A |
| MOL000449 | CA2 |
| MOL000449 | RXRA |
| MOL000449 | ACHE |
| MOL000449 | HTR2A |
| MOL000449 | SLC6A2 |
| MOL000449 | ADRA1A |
| MOL000449 | GABRA3 |
| MOL000449 | PGR |
| MOL000449 | CHRM2 |
| MOL000449 | ADRA1B |
| MOL000449 | SLC6A3 |
| MOL000449 | NR3C2 |
| MOL000449 | ADRB2 |
| MOL000449 | AKR1B1 |
| MOL000449 | NR3C1 |
| MOL000449 | GABRA1 |
| MOL000449 | DPP4 |
| MOL000449 | PLAU |
| MOL000449 | CDK2 |
| MOL000449 | LTA4H |
| MOL000449 | MAOB |
| MOL000449 | MAOA |
| MOL000449 | CHRNA7 |
| MOL000449 | PRKACA |
| MOL000449 | ADH1C |
| MOL000449 | IGHG1 |
| MOL000449 | CTRB1 |
| MOL000449 | PRSS1 |
| MOL000449 | NCOA2 |
| MOL000449 | NCOA1 |
| MOL000737 | ALOX5 |
| MOL000737 | GSR |
| MOL000737 | ABCB1 |
| MOL000737 | XDH |
| MOL000737 | TOP1 |
| MOL000737 | NOS2 |
| MOL000737 | PTGS1 |
| MOL000737 | ESR |
| MOL000737 | AR |
| MOL000737 | PPARG |
| MOL000737 | PTGS2 |
| MOL000737 | CA2 |
| MOL000737 | PTPN1 |
| MOL000737 | ESR2 |
| MOL000737 | DPP4 |
| MOL000737 | MAPK14 |
| MOL000737 | GSK3B |
| MOL000737 | HSP90 |
| MOL000737 | CDK2 |
| MOL000737 | PIK3CG |
| MOL000737 | PIM1 |
| MOL004355 | ESR |
| MOL004355 | AR |
| MOL004355 | PGR |
| MOL004355 | NR3C2 |
| MOL004355 | NR3C1 |
| MOL004355 | NCOA2 |
| MOL004798 | NOS2 |
| MOL004798 | PTGS1 |
| MOL004798 | ESR |
| MOL004798 | AR |
| MOL004798 | PPARG |
| MOL004798 | PTGS2 |
| MOL004798 | CA2 |
| MOL004798 | PTPN1 |
| MOL004798 | ESR2 |
| MOL004798 | DPP4 |
| MOL004798 | MAPK14 |
| MOL004798 | HSP90 |
| MOL004798 | CDK2 |
| MOL004798 | PIK3CG |
| MOL004798 | PIM1 |
| MOL004798 | NCOA2 |
| MOL006767 | NOS2 |
| MOL006767 | F2 |
| MOL006767 | PTGS2 |
| MOL006767 | CA2 |
| MOL006772 | ESR |
| MOL006772 | AR |
| MOL006772 | PGR |
| MOL006772 | NR3C1 |
| MOL006772 | NCOA2 |
| MOL006774 | ESR |
| MOL006774 | AR |
| MOL006774 | PGR |
| MOL006774 | NCOA2 |

Table S3 Detailed information on these known therapeutic targets

| Drugbank Database | GAD Database | OMIM Database | KEGG Database | TTD Database | T-HOD Database |
| --- | --- | --- | --- | --- | --- |
| ABP1 | HGF | BMPR2 | CYP11B2 | ADRA2B | A1BG |
| ACCN1 | GSTP1 | COMMD5 | CYP11B1 | DRD1 | ABCA1 |
| ACCN2 | GSTM1 | AGTR1 | HSD11B2 | DRD2 | ABCA3 |
| ACE | GRK4 | HSD11B2 | NR3C2 | ADRA2C | ABCB1 |
| ACE2 | GPX1 | ACVRL1 | SCNN1B | ADRA1A | ABCG5 |
| ACHE | GNB3 | ATP1A1 | SCNN1G | ADRA1B | ABCG8 |
| ADORA1 | GJA4 | REN |  | ADRA1D | ABO |
| ADORA2A | GCK | ADD1 |  | ADRA2A | ACADSB |
| ADRA1A | FMO3 | WNK4 |  | ADRB2 | ACAT1 |
| ADRA1B | FKBP1B | PPARG |  | ADRB1 | ACE |
| ADRA1D | FGB | CAV1 |  | ADA | ACE2 |
| ADRA2A | F5 | NR3C2 |  | HTR1A | ACHE |
| ADRA2B | F2 | TGFB1 |  | HTR2A | ACP1 |
| ADRA2C | ESR2 | PDE3A |  | HTR2B | ACSM1 |
| ADRB1 | ESR1 | CYP11B1 |  | AVPR1A | ACSM3 |
| ADRB2 | EPHX1 | SCNN1B |  | SLC12A3 | ACTG2 |
| ADRB3 | ENG | KLHL3 |  | SLC12A1 | ACTN2 |
| AGTR1 | EGF | ADM |  | SLC18A2 | ACVRL1 |
| AGTR2 | EDNRB | SMAD9 |  | KCNQ1 | ADAMTS13 |
| AKR1C3 | EDNRA | GRK4 |  | NKCC2 | ADCY10 |
| AOC3 | EDN2 | SCNN1G |  | SCNN1A | ADD1 |
| AR | EDN1 | CD36 |  | CACNA1G | ADD2 |
| ATP1A1 | DRD3 | KCNK3 |  | PTGER2 | ADD3 |
| CA1 | DRD1 | NCF1 |  | PTGFR | ADH5 |
| CA12 | CYP4B1 | NOTCH3 |  | PTGIR | ADIPOQ |
| CA2 | CYP4A11 | HTR2B |  | P4HA1 | ADM |
| CA4 | CYP2S1 | WNK1 |  | ACE | ADM2 |
| CA5A | CYP2J2 | ACSM3 |  | ADORA1 | ADORA1 |
| CA7 | CYP2C9 | SLC6A4 |  | ADORA2A | ADORA2A |
| CA9 | CYP2C8 | CYP11B2 |  | ADORA2B | ADRA1A |
| CACNA1A | CYP2C19 | SMAD1 |  | EDNRA | ADRA1B |
| CACNA1B | CYP11B2 | CALCRL |  | EDNRB | ADRA1D |
| CACNA1C | CYP11B1 | RAMP2 |  | NR3C2 | ADRA2A |
| CACNA1D | CYBA | GUCY1A3 |  | TUBB | ADRA2B |
| CACNA1F | CORIN | LEP |  | CNR1 | ADRA2C |
| CACNA1G | CMA1 | AKT1 |  | MAOB | ADRB1 |
| CACNA1H | CLU | PGF |  | CACNA2D1 | ADRB2 |
| CACNA1I | CLCNKB | CYP4A11 |  | ASIC1 | ADRB3 |
| CACNA1S | CETP | NPR1 |  | PDE3A | ADRBK1 |
| CACNA2D1 | CD36 | GNB3 |  | PDE3B | ADRBK2 |
| CACNA2D2 | CCR5 | RGS2 |  | AGTR1 | AGT |
| CACNA2D3 | CAT | TRPC6 |  | GUCY1B3 | AGTR1 |
| CACNB1 | CASR | ERAP1 |  | ELANE | AGTR2 |
| CACNB2 | CART | ALOX5 |  | MME | AHR |
| CACNB3 | CAPN10 | ELN |  | REN | AHSG |
| CACNB4 | CALCRL | FBN1 |  | CYP2D6 | AHSP |
| CACNG1 | MBOAT5 | SCNN1A |  | CALCR | AKT1 |
| CALM1 | ACSM1 | DGUOK |  | AGTR2 | ALB |
| CHRM1 | BMPR2 | SLC9A1 |  | PTGIR | ALDH2 |
| CHRM2 | BDKRB2 | ACE2 |  | ADRB3 | ALOX12 |
| CHRM3 | BDKRB1 | KYNU |  | HSD11B1 | ALOX15 |
| CHRM4 | ATP1A2 | LRP6 |  | NOS3 | ALOX5 |
| CHRM5 | ARTS-1 | NOS2A |  | SELE | ALOX5AP |
| CHRNA10 | APOE | NPPA |  | NPY1R | ALPP |
| CHRNA2 | APOB | ADRBK1 |  | SELP | AMY1A |
| CHRNA3 | APOA5 | CYP3A5 |  | ROCK1 | ANG |
| CHRNB4 | APOA1 | KCNMB1 |  | VIPR1 | ANGPT1 |
| CYP11B2 | ALDH2 | TNFRSF1B |  | ADRA1A | ANGPT2 |
| DDC | ALAD | VIP |  | ADRA2B | ANKRD1 |
| DRD1 | AGTR2 | KCNJ5 |  | ACE | ANPEP |
| DRD5 | AGTR1 | ADD2 |  | ATP1A3 | ANXA1 |
| EDNRA | AGT | CORIN |  | NOS1 | APEX1 |
| EDNRB | ADRB3 | ABCC6 |  | VIPR2 | APLN |
| FXYD2 | ADRB2 | APOL1 |  | PDK2 | APLNR |
| GJA1 | ADRB1 | YY1AP1 |  | ADRB2 | APOA1 |
| GUCY1A2 | ADRA2B | SLC39A12 |  | PRTN3 | APOB |
| HIF1A | ADRA2A | CYP17A1 |  | CYP11B2 | APOBEC3F |
| HRH1 | ADRA1B | MMACHC |  | MYBPC3 | APOC3 |
| HRH2 | ADRA1A | MEX3C |  | CTSD | APOE |
| HSD11B1 | ADORA2A | SARS2 |  | ENPEP | APOL1 |
| HTR1A | ADORA1 | GH1 |  | ADRA2C | APP |
| HTR1B | ADM | GNA11 |  | EPHX2 | AQP1 |
| JUN | ADD3 | PTPN1 |  | NPPB | AQP2 |
| KCNA1 | ADD2 | DRD1 |  | CYBB | AQP4 |
| KCNE1 | ADD1 | PTGIR |  | S1PR2 | AR |
| KCNH2 | ADA | CBLN2 |  | CA4 | ARAP1 |
| KCNH6 | ACE2 | HSD11B1 |  | BDKRB2 | ARHGEF1 |
| KCNH7 | ACE | GNAQ |  | GATA4 | ARHGEF12 |
| KCNJ1 | ADIPOQ | MYOC |  | CYBB | ARL6IP5 |
| KCNJ11 | ABCB1 | PTGIS |  | ERAP1 | ARRDC3 |
| KCNMA1 | TNF | ESR2 |  | NCF1 | ATF1 |
| KCNQ1 | TH | GNA13 |  | AGT | ATP1A1 |
| LTA4H | TGFB3 | ARHGEF12 |  | SLC12A6 | ATP1A2 |
| MAOA | TGFB1 | RETN |  | ABCC9 | ATP1B1 |
| MAOB | SPP1 | PDE5A |  | STK39 | ATP2A2 |
| MME | SOD3 | CTH |  |  | ATP2B1 |
| MMP2 | SOD2 | TNNT2 |  |  | ATP5G1 |
| NDUFC2 | SLCO1B1 | G6PC3 |  |  | ATP6AP2 |
| NPPB | SLCO1A2 | CETP |  |  | ATP6V0A1 |
| NPR1 | SLC9A3 | CMA1 |  |  | ATP6V1B1 |
| NR3C1 | SLC8A1 | MTHFR |  |  | ATP8 |
| NR3C2 | SLC6A4 | GNA12 |  |  | ATXN3 |
| P2RY12 | SLC6A2 | GYS1 |  |  | AVP |
| P4HA1 | SLC12A3 | GNAS |  |  | AVPR1A |
| PAH | SLC12A1 | KLK1 |  |  | AVPR2 |
| PDE11A | SGK | MDK |  |  | AXL |
| PDE1A | SERPINE1 | CBL |  |  | B2M |
| PDE1B | SERPINC1 | RHOA |  |  | BBS4 |
| PDE4A | SELE | ENPP1 |  |  | BCL2 |
| PDE4B | SCNN1G | S100A1 |  |  | BCL2L1 |
| PDE4C | SCNN1B | MMP2 |  |  | BCR |
| PDE4D | SCNN1A | PDE4D |  |  | BDKRB1 |
| PDE5A | SCN7A | ECE1 |  |  | BDKRB2 |
| PDE6G | RGS2 | PKD1 |  |  | BLK |
| PDE6H | RETN | ADD3 |  |  | BMP1 |
| PDXK | RENBP | APOE |  |  | BMP2 |
| PGR | REN | CAT |  |  | BMP4 |
| PLAT | PTPN1 | VAV3 |  |  | BMP6 |
| PLAU | PTGS2 | HIF1A |  |  | BMPR2 |
| PPARA | PTGIS | CACNA1H |  |  | BNIP1 |
| PPARD | PTGES | MIR155 |  |  | BRIP1 |
| PPARG | PPARGC1A | PF4 |  |  | BTN2A1 |
| PTGER1 | PPARG | ARHGEF1 |  |  | C2 |
| PTGER3 | PPARD | GCGR |  |  | C3 |
| PTGFR | PNMT | HLA-DPB1 |  |  | C4A |
| PTGIR | PLIN | FLT1 |  |  | C5AR1 |
| PTGIS | PLAT | PLA2G1B |  |  | CA2 |
| PTGS1 | PIK3R1 | SLC8A1 |  |  | CABIN1 |
| REN | PCSK1 | DRD3 |  |  | CACNA1C |
| SCN5A | P2RX4 | EDNRB |  |  | CACNA1D |
| SCNN1A | NR3C2 | FGA |  |  | CACNA1H |
| SCNN1B | NR3C1 | FMO3 |  |  | CACNB2 |
| SCNN1D | NPY | MTTI |  |  | CALCA |
| SCNN1G | NPR3 | BBS4 |  |  | CALCR |
| SELE | NPR2 | LEPR |  |  | CALM1 |
| SHBG | NPR1 | SCN2B |  |  | CAMK2G |
| SLC12A1 | NPPC | DYNLT1 |  |  | CAPN10 |
| SLC12A2 | NPPA | SERPINA1 |  |  | CARTPT |
| SLC12A3 | ANP | NPR3 |  |  | CASP3 |
| SLC18A1 | NPHS2 | HSD3B1 |  |  | CASP8 |
| SLC18A2 | NPHS1 | CAST |  |  | CASP9 |
| SLC6A2 | NOS2A | CACNA1D |  |  | CAST |
| SLC6A3 | NOS1 | HTRA1 |  |  | CAT |
| SLC6A4 | NEDD4L | CFTR |  |  | CAV1 |
| SLC9A1 | MYOC | DDAH1 |  |  | CAV2 |
| SMPD1 | MMP3 | MKKS |  |  | CCL2 |
| SRD5A3 | MMP1 | BIRC5 |  |  | CCL23 |
| TNNC1 | MC4R | BBS2 |  |  | CCL5 |
| TNNC2 | LTA | EIF2AK4 |  |  | CCNA2 |
| TRDMT1 | LPL | ARHGAP42 |  |  | CCND1 |
| VCAM1 | LIPC | CHGA |  |  | CCR5 |
| VEGFA | LEP | L3MBTL4 |  |  | CCR7 |
|  | LDLR | NR3C1 |  |  | CD19 |
|  | KLK2 | SLC2A4 |  |  | CD2 |
|  | KLK1 | INSR |  |  | CD200 |
|  | KCNMB1 | IL1A |  |  | CD34 |
|  | ITGA2 | LMNB2 |  |  | CD36 |
|  | IRS1 | MTR |  |  | CD4 |
|  | INSR | TAC3 |  |  | CD59 |
|  | INS | PAX2 |  |  | CD63 |
|  | IL1RN | SERPINE1 |  |  | CD8A |
|  | IL1B | POMC |  |  | CDH13 |
|  | IL1A | F2 |  |  | CDH15 |
|  | IGF1 | HRAS |  |  | CDH5 |
|  | HTR2A | COL4A2 |  |  | CDK5R1 |
|  | HSD3B1 | GJA4 |  |  | CDKN1A |
|  | HSD11B2 | CRH |  |  | CDKN2A |
|  | HSD11B1 | MSX2 |  |  | CDKN2B |
|  | HPCAL1 | COX8A |  |  | CEBPZ |
|  | HMOX1 | POR |  |  | CETP |
|  | HLA-DQA1 | EMILIN1 |  |  | CFH |
|  | HLA-DPB1 | EPOR |  |  | CHEK2 |
|  | HLA-B | F13A1 |  |  | CHGA |
|  | HLA-A | FBL |  |  | CIMT |
|  | TNFRSF1B | FGF2 |  |  | CKS1B |
|  | TRHR | SCN5A |  |  | CLCN3 |
|  | UCP2 | JAK3 |  |  | CLCNKB |
|  | VDR | NPPB |  |  | CLOCK |
|  | WNK1 | ABCC8 |  |  | CLU |
|  | PRKWNK1 | P2RY12 |  |  | CMA1 |
|  | WNK4 | NFKBIL1 |  |  | CMYA5 |
|  | APOC3 | BMPR1A |  |  | COG2 |
|  | ATP1A1 | SCNN1D |  |  | COL1A2 |
|  | CCR2 | VEGFC |  |  | COL4A1 |
|  | CNP | ABCA3 | 590 |  | COL4A3 |
|  | CYP3A4 | NHEDC2 |  |  | COMT |
|  | CYP3A5 | CC2D2A |  |  | CORIN |
|  | DBH | TMEM70 |  |  | COX1 |
|  | GNAS | LIPT1 |  |  | COX4NB |
|  | GYPA | SLC2A12 |  |  | CP |
|  | HLA-DRB1 | AGK |  |  | CPB2 |
|  | IL6 | PIEZO1 |  |  | CPE |
|  | LEPR | SLC30A10 |  |  | CREBBP |
|  | MTHFR | BICC1 |  |  | CRH |
|  | MTR | PLAT |  |  | CRHR2 |
|  | NOS3 | CRELD1 |  |  | CRP |
|  | NTRK3 | MYO18B |  |  | CRY1 |
|  | PPARA | MC2R |  |  | CSK |
|  | SAA1 | ADAMTS16 |  |  | CSMD1 |
|  | SA | NFU1 |  |  | CST3 |
|  | SAH | SATB2 |  |  | CTF1 |
|  | ACSM3 | CFI |  |  | CTGF |
|  | GHRL | STOX1 |  |  | CTLA4 |
|  | Hb | XPNPEP3 |  |  | CTSB |
|  | KYNU | HSD3B2 |  |  | CX3CL1 |
|  | ATM | ZFPM2 |  |  | CXCL10 |
|  | GCGR | GUCY1B2 |  |  | CXCL12 |
|  | ITGB3 | NDUFA10 |  |  | CXCL5 |
|  | ACADSB | TINF2 |  |  | CXCR1 |
|  | ALOX12 | BSCL2 |  |  | CXCR2 |
|  | ATP2A2 | MTPN |  |  | CXCR4 |
|  | BSND | EGLN1 |  |  | CYBA |
|  | C10orf59 | ABI2 |  |  | CYBB |
|  | CACNA1C | PKHD1 |  |  | CYP11A1 |
|  | CALCA | WT1 |  |  | CYP11B1 |
|  | CAPN5 | FLNA |  |  | CYP11B2 |
|  | CAST | MBTPS2 |  |  | CYP17A1 |
|  | CAV1 | LTBP4 |  |  | CYP19A1 |
|  | CD40 | NPHS2 |  |  | CYP1A1 |
|  | CES1 | PDE11A |  |  | CYP1A2 |
|  | CLCNKA | CRTAP |  |  | CYP1B1 |
|  | CNR2 | PRDM16 |  |  | CYP21A2 |
|  | COMT | CUL3 |  |  | CYP27B1 |
|  | CPS1 | RGS5 |  |  | CYP2C19 |
|  | CTNS | PDE8B |  |  | CYP2C8 |
|  | CX3CR1 | SCARB1 |  |  | CYP2C9 |
|  | CYP1B1 | RNU4ATAC |  |  | CYP2D6 |
|  | CYP4F2 | GATA6 |  |  | CYP2J2 |
|  | DIO2 | NPR2 |  |  | CYP3A4 |
|  | ELN | CALCA |  |  | CYP3A5 |
|  | ENPP1 | TBX1 |  |  | CYP3A7 |
|  | F11 | NPHS1 |  |  | CYP4A11 |
|  | FABP2 | KCNN4 |  |  | CYP4F2 |
|  | FSHR | KCNN3 |  |  | CYP7A1 |
|  | FYN | ATP2B3 |  |  | CYR61 |
|  | GH1 | SDHB |  |  | CYSLTR2 |
|  | GHR | COL4A1 |  |  | DBH |
|  | GJA5 | CD46 |  |  | DCN |
|  | GYS1 | SERPINA6 |  |  | DCP2 |
|  | HLA-DQB1 | SELE |  |  | DDAH1 |
|  | IKBKAP | FN1 |  |  | DDAH2 |
|  | INPPL1 | MTTL1 |  |  | DDIT3 |
|  | KCNJ11 | FGF8 |  |  | DEFA1 |
|  | KLKB1 | PTPRO |  |  | DMD |
|  | LIPE | PRLHR |  |  | DNAJB11 |
|  | LRP5 | GCK |  |  | DNASE1 |
|  | MMP9 | GUCY1B3 |  |  | DNTT |
|  | MT-ND2 | LMNA |  |  | DPP4 |
|  | NLRP3 | PRCP |  |  | DRD1 |
|  | P2RY2 | PRKG1 |  |  | DRD2 |
|  | PON1 | SFTPB |  |  | DRD3 |
|  | PON2 | ATP1B1 |  |  | DRD4 |
|  | SLC14A2 | IRS2 |  |  | DSP |
|  | SLC4A1 | TLR5 |  |  | E2F2 |
|  | SLC6A18 | ASL |  |  | ECE1 |
|  | SLC7A1 | UMOD |  |  | EDN1 |
|  | SLC9A2 | VCL |  |  | EDN2 |
|  | TRH | PLCE1 |  |  | EDN3 |
|  | UMOD | TRIM72 |  |  | EDNRA |
|  | UTS2 | ZNF260 |  |  | EDNRB |
|  | SLC22A2 | HSPB7 |  |  | EGF |
|  | ADRA2C | NOTCH1 |  |  | EGFR |
|  | COL1A2 | TGFB2 |  |  | EGLN1 |
|  | NPPB | SELP |  |  | EGLN3 |
|  | CFH | PKD2 |  |  | EGR1 |
|  | CHGA | ADAMTS13 |  |  | ELANE |
|  | QPCT | RALBP1 |  |  | ELN |
|  | VWF | SUCNR1 |  |  | EMILIN1 |
|  | RABIF | NEDD4L |  |  | ENG |
|  | PYY | GP1BA |  |  | ENPP1 |
|  | PYGB | LDLR |  |  | EP300 |
|  | PTPRN2 | UTP4 |  |  | EPAS1 |
|  | PTGS1 | CECR1 |  |  | EPHX1 |
|  | PSMB9 | TH |  |  | EPHX2 |
|  | PSMB8 | USF1 |  |  | EPO |
|  | PSMB4 | ESRRG |  |  | EPOR |
|  | PSMA6 | BRS3 |  |  | ERAP2 |
|  | PSMA4 | FHL1 |  |  | ERI1 |
|  | PRSS8 | IKBKG |  |  | ESR1 |
|  | PRKG1 | DYRK1B |  |  | ESR2 |
|  | PRKCQ | ACTN4 |  |  | F11R |
|  | ACAT2 | NFAT5 |  |  | F2 |
|  | PRELP | DLL4 |  |  | F3 |
|  | PRC1 | FSTL3 |  |  | F5 |
|  | PPFIA4 | MYOZ2 |  |  | FAAH |
|  | POU2F1 | GTF2I |  |  | FABP2 |
|  | PMS1 | ACCN1 |  |  | FABP3 |
|  | PML | IFNGR1 |  |  | FABP6 |
|  | PLAGL1 | ATP2A2 |  |  | FANCB |
|  | PKD1 | UCP1 |  |  | FAS |
|  | PKD2 | CDKN1A |  |  | FASLG |
|  | PIK3C2B | NEDD4 |  |  | FBN1 |
|  | SERPINE2 | OGN |  |  | FGF2 |
|  | SERPINA1 | SDHD |  |  | FGF5 |
|  | PGR | TNFSF12 |  |  | FGFBP1 |
|  | PECAM1 |  |  |  | FKBP1A |
|  | PDE4D |  |  |  | FLT3LG |
|  | PCSK2 |  |  |  | FMO3 |
|  | PBX1 |  |  |  | FN1 |
|  | PANX2 |  |  |  | FOS |
|  | P2RX7 |  |  |  | FSHR |
|  | ATP6V1B1 |  |  |  | FURIN |
|  | ATP2B4 |  |  |  | FUT1 |
|  | OPTC |  |  |  | G6PC |
|  | ATP2B1 |  |  |  | G6PD |
|  | NVL |  |  |  | GABBR1 |
|  | ATP1B1 |  |  |  | GABRA6 |
|  | NR2F1 |  |  |  | GAL |
|  | NPY2R |  |  |  | GAPDH |
|  | COX4NB |  |  |  | GATA4 |
|  | NFKBIL1 |  |  |  | GATA5 |
|  | NDUFAB1 |  |  |  | GATA6 |
|  | NDST1 |  |  |  | GC |
|  | NCOR2 |  |  |  | GCA |
|  | PPP1R12B |  |  |  | GCG |
|  | MYO7B |  |  |  | GCGR |
|  | MYLK |  |  |  | GCH1 |
|  | MYH9 |  |  |  | GCLC |
|  | MYBPH |  |  |  | GDF15 |
|  | MYB |  |  |  | GDNF |
|  | MTRR |  |  |  | GGT1 |
|  | MTTP |  |  |  | GH1 |
|  | MTAP |  |  |  | GHR |
|  | MS4A2 |  |  |  | GHRH |
|  | ARVCF |  |  |  | GHRL |
|  | MPZL1 |  |  |  | GJA1 |
|  | CIITA |  |  |  | GJA4 |
|  | MGST3 |  |  |  | GJA5 |
|  | MEF2D |  |  |  | GJB1 |
|  | MCC |  |  |  | GJC1 |
|  | MCCC2 |  |  |  | GLI1 |
|  | MBL2 |  |  |  | GLO1 |
|  | SMAD5 |  |  |  | GLUL |
|  | SMAD6 |  |  |  | GNA12 |
|  | SMAD4 |  |  |  | GNA14 |
|  | SMAD3 |  |  |  | GNAI1 |
|  | SMAD2 |  |  |  | GNAS |
|  | SMAD1 |  |  |  | GNB2L1 |
|  | LZTR1 |  |  |  | GNB3 |
|  | LTC4S |  |  |  | GNG2 |
|  | LTBP2 |  |  |  | GORASP1 |
|  | LRPAP1 |  |  |  | GPA33 |
|  | LRP8 |  |  |  | GPR25 |
|  | LPA |  |  |  | GPR98 |
|  | LMX1B |  |  |  | GPX1 |
|  | LNPEP |  |  |  | GRK1 |
|  | LMX1A |  |  |  | GRK4 |
|  | LLGL2 |  |  |  | GRK5 |
|  | LGALS2 |  |  |  | GRK6 |
|  | SLC25A5P2 |  |  |  | GRP |
|  | LOC100131938 |  |  |  | GSK3B |
|  | LOC730226 | |  |  | GSTM1 |
|  | NUF2 |  |  |  | GSTM3 |
|  | TMEM183A |  |  |  | GSTM5 |
|  | CCDC141 |  |  |  | GSTT1 |
|  | LOC100128751 |  |  |  | GTPBP4 |
|  | LOC100131402 | |  |  | GUCA2B |
|  | ILDR2 | |  |  | GYS1 |
|  | KRT18P16 |  |  |  | H19 |
|  | KRT18P42 |  |  |  | HAO2 |
|  | AQPEP |  |  |  | HBA1 |
|  | MEGF11 |  |  |  | HBB |
|  | TRAPPC9 |  |  |  | HCRT |
|  | CUL9 |  |  |  | HDAC2 |
|  | FAM13A |  |  |  | HDLBP |
|  | TRNM |  |  |  | HFE |
|  | ERAP2 |  |  |  | HGF |
|  | P2RX6 |  |  |  | HIF1A |
|  | APLNR |  |  |  | HLA-A |
|  | ATL1 |  |  |  | HLA-B |
|  | ADCY10 |  |  |  | HLA-DOA |
|  | TRNQ |  |  |  | HLA-DPB1 |
|  | TRNL1 |  |  |  | HLA-DQA1 |
|  | TRNK |  |  |  | HLA-DQA2 |
|  | TRNI |  |  |  | HLA-DQB1 |
|  | PALB2 |  |  |  | HLA-DRB1 |
|  | ERAP1 |  |  |  | HMGCR |
|  | CLEC16A |  |  |  | HMOX1 |
|  | ND2 |  |  |  | HMOX2 |
|  | HNF1A |  |  |  | HNF1A |
|  | TCF25 |  |  |  | HNRNPAB |
|  | SH2B3 |  |  |  | HOXA5 |
|  | CCDC86 |  |  |  | HP |
|  | CD247 |  |  |  | HPCAL1 |
|  | OSGIN1 |  |  |  | HPGD |
|  | ECOP |  |  |  | HRAS |
|  | LAMA3 |  |  |  | HRH2 |
|  | KLC1 |  |  |  | HSD11B1 |
|  | KNG1 |  |  |  | HSD11B2 |
|  | KL |  |  |  | HSD3B1 |
|  | KISS1 |  |  |  | HSF1 |
|  | ZFP30 |  |  |  | HSP90AA1 |
|  | NOS2 |  |  |  | HSPA1A |
|  | KCNMA1 |  |  |  | HSPA4 |
|  | GDF15 |  |  |  | HSPA8 |
|  | KCNJ1 |  |  |  | HSPB2 |
|  | KCNB1 |  |  |  | HSPB7 |
|  | IVD |  |  |  | HSPD1 |
|  | ITPK1 |  |  |  | HTR1A |
|  | ITM2C |  |  |  | HTR2A |
|  | IRF5 |  |  |  | HTR2B |
|  | IRAK1 |  |  |  | IAPP |
|  | FTO |  |  |  | ICAM1 |
|  | APOC1 |  |  |  | ID1 |
|  | IL9 |  |  |  | IER3 |
|  | TRDMT1 |  |  |  | IFNA1 |
|  | APOA4 |  |  |  | IFNG |
|  | IL5RA |  |  |  | IGF1 |
|  | IL5 |  |  |  | IGF1R |
|  | IL4R |  |  |  | IGF2 |
|  | IL4 |  |  |  | IGFBP1 |
|  | IL3 |  |  |  | IGFBP2 |
|  | IL21R |  |  |  | IGKV1D-39 |
|  | IL18 |  |  |  | IKBKAP |
|  | IL13 |  |  |  | IKBKB |
|  | IL10 |  |  |  | IL10 |
|  | AP3D1 |  |  |  | IL11 |
|  | IGF2 |  |  |  | IL13 |
|  | IGF1R |  |  |  | IL13RA1 |
|  | ICAM1 |  |  |  | IL13RA2 |
|  | HSPA8 |  |  |  | IL15 |
|  | HSPA1L |  |  |  | IL17A |
|  | HSPA1B |  |  |  | IL18 |
|  | HSD17B7 |  |  |  | IL1A |
|  | HP |  |  |  | IL1B |
|  | ABCC1 |  |  |  | IL1F8 |
|  | HMOX2 |  |  |  | IL1R1 |
|  | ANPEP |  |  |  | IL1RN |
|  | HFE |  |  |  | IL2 |
|  | HBS1L |  |  |  | IL2RA |
|  | ANG |  |  |  | IL4 |
|  | GSTT1 |  |  |  | IL6 |
|  | GSTM5 |  |  |  | IL6ST |
|  | GSTM4 |  |  |  | IL7 |
|  | GSTM3 |  |  |  | IL8 |
|  | GSTM2 |  |  |  | ILF3 |
|  | GPA33 |  |  |  | ILK |
|  | GOSR2 |  |  |  | INPPL1 |
|  | ALPL |  |  |  | INS |
|  | ALOX5AP |  |  |  | INSR |
|  | ALOX5 |  |  |  | IRS1 |
|  | GLRX |  |  |  | ISYNA1 |
|  | GC |  |  |  | ITGA2 |
|  | GAS7 |  |  |  | ITGA4 |
|  | ALDH9A1 |  |  |  | ITGAL |
|  | FUT7 |  |  |  | ITGAM |
|  | ALDH1L1 |  |  |  | ITGB1BP2 |
|  | FOXF1 |  |  |  | JAK1 |
|  | FOLH1 |  |  |  | JAK2 |
|  | FMOD |  |  |  | JUN |
|  | AKAP12 |  |  |  | KCNA5 |
|  | FGG |  |  |  | KCNB1 |
|  | FGA |  |  |  | KCNE1 |
|  | FABP3 |  |  |  | KCNJ1 |
|  | FAAH |  |  |  | KCNJ10 |
|  | F7 |  |  |  | KCNJ11 |
|  | F13A1 |  |  |  | KCNK2 |
|  | F12 |  |  |  | KCNMB1 |
|  | F10 |  |  |  | KCNMB4 |
|  | EPHX2 |  |  |  | KCNN3 |
|  | EPAS1 |  |  |  | KCNN4 |
|  | LRRC52 |  |  |  | KCNQ1 |
|  | EDIL3 |  |  |  | KDR |
|  | WDR37 |  |  |  | KISS1 |
|  | ZSWIM2 |  |  |  | KITLG |
|  | TADA1L |  |  |  | KLK1 |
|  | PDGFD |  |  |  | KLK11 |
|  | TIPRL |  |  |  | KLKB1 |
|  | DPT |  |  |  | KNG1 |
|  | NUP210 |  |  |  | KYNU |
|  | IQWD1 |  |  |  | LDLR |
|  | NTAN1 |  |  |  | LDOC1 |
|  | DOCK1 |  |  |  | LEP |
|  | NADSYN1 |  |  |  | LEPR |
|  | ZNF618 |  |  |  | LGALS2 |
|  | AADACL1 |  |  |  | LGALS3 |
|  | ERGIC1 |  |  |  | LINGO1 |
|  | DIP2C |  |  |  | LIPC |
|  | ZC3H11A |  |  |  | LMNA |
|  | ZC3H3 |  |  |  | LMX1B |
|  | C1ORF156 |  |  |  | LNPEP |
|  | C1ORF110 |  |  |  | LPA |
|  | DHFR |  |  |  | LPCAT3 |
|  | RCSD1 |  |  |  | LPIN1 |
|  | ZNF607 |  |  |  | LPL |
|  | C1ORF114 |  |  |  | MA |
|  | SLC6A19 |  |  |  | MAOB |
|  | ADAMTSL5 |  |  |  | MAP1LC3B |
|  | DDR2 |  |  |  | MAP2K1 |
|  | DDAH2 |  |  |  | MAPK1 |
|  | PLEKHA7 |  |  |  | MAPK14 |
|  | DIDO1 |  |  |  | MAPK3 |
|  | C1ORF125 |  |  |  | MAPK9 |
|  | CYP2D6 |  |  |  | MAPKAPK2 |
|  | CYP27B1 |  |  |  | MAPT |
|  | CYP24A1 |  |  |  | MARK2 |
|  | CASZ1 |  |  |  | MAS1 |
|  | LAX1 |  |  |  | MAT1A |
|  | PIGG |  |  |  | MAT2B |
|  | CYP1A2 |  |  |  | MC3R |
|  |  |  |  |  | MC4R |
|  |  |  |  |  | MCAT |
|  |  |  |  |  | MEF2A |
|  |  |  |  |  | MEOX2 |
|  |  |  |  |  | MFN2 |
|  |  |  |  |  | MGAM |
|  |  |  |  |  | MIR155 |
|  |  |  |  |  | MIR17 |
|  |  |  |  |  | MIR200A |
|  |  |  |  |  | MKKS |
|  |  |  |  |  | MMP1 |
|  |  |  |  |  | MMP14 |
|  |  |  |  |  | MMP2 |
|  |  |  |  |  | MMP3 |
|  |  |  |  |  | MMP7 |
|  |  |  |  |  | MMP8 |
|  |  |  |  |  | MMP9 |
|  |  |  |  |  | MPV17 |
|  |  |  |  |  | MRC1 |
|  |  |  |  |  | MSR1 |
|  |  |  |  |  | MTHFR |
|  |  |  |  |  | MTMR9 |
|  |  |  |  |  | MTPN |
|  |  |  |  |  | MTR |
|  |  |  |  |  | MUC5AC |
|  |  |  |  |  | MYC |
|  |  |  |  |  | MYH9 |
|  |  |  |  |  | MYOC |
|  |  |  |  |  | MYOCD |
|  |  |  |  |  | NA |
|  |  |  |  |  | NBN |
|  |  |  |  |  | NCAM1 |
|  |  |  |  |  | NCF1 |
|  |  |  |  |  | NDFIP2 |
|  |  |  |  |  | NDUFC2 |
|  |  |  |  |  | NEDD4 |
|  |  |  |  |  | NEDD4L |
|  |  |  |  |  | NEUROD1 |
|  |  |  |  |  | NF1 |
|  |  |  |  |  | NFATC2 |
|  |  |  |  |  | NFATC3 |
|  |  |  |  |  | NFKB1 |
|  |  |  |  |  | NFKBIB |
|  |  |  |  |  | NGF |
|  |  |  |  |  | NOS1 |
|  |  |  |  |  | NOS2 |
|  |  |  |  |  | NOS3 |
|  |  |  |  |  | NOTCH4 |
|  |  |  |  |  | NOX1 |
|  |  |  |  |  | NOX4 |
|  |  |  |  |  | NPCDR1 |
|  |  |  |  |  | NPHS1 |
|  |  |  |  |  | NPHS2 |
|  |  |  |  |  | NPPA |
|  |  |  |  |  | NPPB |
|  |  |  |  |  | NPPC |
|  |  |  |  |  | NPR1 |
|  |  |  |  |  | NPR3 |
|  |  |  |  |  | NPY |
|  |  |  |  |  | NPY1R |
|  |  |  |  |  | NPY2R |
|  |  |  |  |  | NPY5R |
|  |  |  |  |  | NPY6R |
|  |  |  |  |  | NR1H4 |
|  |  |  |  |  | NR1I2 |
|  |  |  |  |  | NR3C1 |
|  |  |  |  |  | NR3C2 |
|  |  |  |  |  | NR4A2 |
|  |  |  |  |  | NT5E |
|  |  |  |  |  | NTRK1 |
|  |  |  |  |  | OLR1 |
|  |  |  |  |  | OXT |
|  |  |  |  |  | P2RY2 |
|  |  |  |  |  | PAG1 |
|  |  |  |  |  | PAH |
|  |  |  |  |  | PAK1 |
|  |  |  |  |  | PAPPA |
|  |  |  |  |  | PAPSS1 |
|  |  |  |  |  | PARK2 |
|  |  |  |  |  | PARP1 |
|  |  |  |  |  | PCNA |
|  |  |  |  |  | PCSK6 |
|  |  |  |  |  | PDC |
|  |  |  |  |  | PDE3A |
|  |  |  |  |  | PDE4D |
|  |  |  |  |  | PDE5A |
|  |  |  |  |  | PDGFB |
|  |  |  |  |  | PDGFD |
|  |  |  |  |  | PDGFRB |
|  |  |  |  |  | PDIK1L |
|  |  |  |  |  | PDPK1 |
|  |  |  |  |  | PDYN |
|  |  |  |  |  | PECAM1 |
|  |  |  |  |  | PENK |
|  |  |  |  |  | PEPD |
|  |  |  |  |  | PFN1 |
|  |  |  |  |  | PGD |
|  |  |  |  |  | PHA2A |
|  |  |  |  |  | PHB2 |
|  |  |  |  |  | PHEX |
|  |  |  |  |  | PIK3R1 |
|  |  |  |  |  | PIM1 |
|  |  |  |  |  | PKD1 |
|  |  |  |  |  | PLA2G1B |
|  |  |  |  |  | PLA2G7 |
|  |  |  |  |  | PLAT |
|  |  |  |  |  | PLAU |
|  |  |  |  |  | PLAUR |
|  |  |  |  |  | PLCD1 |
|  |  |  |  |  | PLCD3 |
|  |  |  |  |  | PLD2 |
|  |  |  |  |  | PLEKHA7 |
|  |  |  |  |  | PLG |
|  |  |  |  |  | PNMT |
|  |  |  |  |  | POMC |
|  |  |  |  |  | PON1 |
|  |  |  |  |  | PON2 |
|  |  |  |  |  | POU5F1 |
|  |  |  |  |  | PPARA |
|  |  |  |  |  | PPARG |
|  |  |  |  |  | PPARGC1A |
|  |  |  |  |  | PPBP |
|  |  |  |  |  | PPIG |
|  |  |  |  |  | PPP1R12A |
|  |  |  |  |  | PPP1R14A |
|  |  |  |  |  | PPP3CA |
|  |  |  |  |  | PPP3R1 |
|  |  |  |  |  | PPY |
|  |  |  |  |  | PRAM1 |
|  |  |  |  |  | PRCP |
|  |  |  |  |  | PRDX3 |
|  |  |  |  |  | PRDX5 |
|  |  |  |  |  | PREP |
|  |  |  |  |  | PRKAA1 |
|  |  |  |  |  | PRKAA2 |
|  |  |  |  |  | PRKCA |
|  |  |  |  |  | PRKCG |
|  |  |  |  |  | PRKG1 |
|  |  |  |  |  | PRL |
|  |  |  |  |  | PROC |
|  |  |  |  |  | PROK1 |
|  |  |  |  |  | PROM1 |
|  |  |  |  |  | PROS1 |
|  |  |  |  |  | PRRX1 |
|  |  |  |  |  | PRSS8 |
|  |  |  |  |  | PSMA6 |
|  |  |  |  |  | PSMB9 |
|  |  |  |  |  | PTEN |
|  |  |  |  |  | PTGIR |
|  |  |  |  |  | PTGIS |
|  |  |  |  |  | PTGS1 |
|  |  |  |  |  | PTGS2 |
|  |  |  |  |  | PTH |
|  |  |  |  |  | PTHLH |
|  |  |  |  |  | PTPN1 |
|  |  |  |  |  | PTPRO |
|  |  |  |  |  | QPCT |
|  |  |  |  |  | RABGAP1L |
|  |  |  |  |  | RAC1 |
|  |  |  |  |  | RAF1 |
|  |  |  |  |  | RAMP1 |
|  |  |  |  |  | RAMP2 |
|  |  |  |  |  | RAMP3 |
|  |  |  |  |  | RARRES2 |
|  |  |  |  |  | RCAN1 |
|  |  |  |  |  | REN |
|  |  |  |  |  | RENBP |
|  |  |  |  |  | RET |
|  |  |  |  |  | RETN |
|  |  |  |  |  | RETNLB |
|  |  |  |  |  | RFC2 |
|  |  |  |  |  | RFFL |
|  |  |  |  |  | RGS2 |
|  |  |  |  |  | RGS20 |
|  |  |  |  |  | RGS5 |
|  |  |  |  |  | RHO |
|  |  |  |  |  | RHOA |
|  |  |  |  |  | RHOD |
|  |  |  |  |  | RLN1 |
|  |  |  |  |  | RNLS |
|  |  |  |  |  | ROCK1 |
|  |  |  |  |  | ROCK2 |
|  |  |  |  |  | ROS1 |
|  |  |  |  |  | RPS6KA3 |
|  |  |  |  |  | RTN4 |
|  |  |  |  |  | RYR2 |
|  |  |  |  |  | S100A1 |
|  |  |  |  |  | S100A4 |
|  |  |  |  |  | S100A6 |
|  |  |  |  |  | S100A8 |
|  |  |  |  |  | S1PR1 |
|  |  |  |  |  | S1PR2 |
|  |  |  |  |  | SCG2 |
|  |  |  |  |  | SCN2A |
|  |  |  |  |  | SCN7A |
|  |  |  |  |  | SCNN1A |
|  |  |  |  |  | SCNN1B |
|  |  |  |  |  | SCNN1G |
|  |  |  |  |  | SDK1 |
|  |  |  |  |  | SELE |
|  |  |  |  |  | SELL |
|  |  |  |  |  | SELP |
|  |  |  |  |  | SERPINA1 |
|  |  |  |  |  | SERPINA3 |
|  |  |  |  |  | SERPINA4 |
|  |  |  |  |  | SERPINA6 |
|  |  |  |  |  | SERPINB2 |
|  |  |  |  |  | SERPINE1 |
|  |  |  |  |  | SERPINE2 |
|  |  |  |  |  | SF1 |
|  |  |  |  |  | SGK1 |
|  |  |  |  |  | SGPL1 |
|  |  |  |  |  | SHC1 |
|  |  |  |  |  | SIRT1 |
|  |  |  |  |  | SLC12A1 |
|  |  |  |  |  | SLC12A2 |
|  |  |  |  |  | SLC12A3 |
|  |  |  |  |  | SLC12A6 |
|  |  |  |  |  | SLC14A2 |
|  |  |  |  |  | SLC16A1 |
|  |  |  |  |  | SLC17A5 |
|  |  |  |  |  | SLC22A2 |
|  |  |  |  |  | SLC22A3 |
|  |  |  |  |  | SLC22A6 |
|  |  |  |  |  | SLC24A4 |
|  |  |  |  |  | SLC26A4 |
|  |  |  |  |  | SLC26A6 |
|  |  |  |  |  | SLC2A1 |
|  |  |  |  |  | SLC2A2 |
|  |  |  |  |  | SLC2A4 |
|  |  |  |  |  | SLC2A5 |
|  |  |  |  |  | SLC2A9 |
|  |  |  |  |  | SLC34A2 |
|  |  |  |  |  | SLC4A1 |
|  |  |  |  |  | SLC4A2 |
|  |  |  |  |  | SLC4A4 |
|  |  |  |  |  | SLC4A8 |
|  |  |  |  |  | SLC5A1 |
|  |  |  |  |  | SLC6A2 |
|  |  |  |  |  | SLC6A4 |
|  |  |  |  |  | SLC6A9 |
|  |  |  |  |  | SLC8A1 |
|  |  |  |  |  | SLC9A1 |
|  |  |  |  |  | SLC9A3 |
|  |  |  |  |  | SLC9A3R1 |
|  |  |  |  |  | SLC9A3R2 |
|  |  |  |  |  | SMAD1 |
|  |  |  |  |  | SMAD2 |
|  |  |  |  |  | SMAD3 |
|  |  |  |  |  | SMAD7 |
|  |  |  |  |  | SMTN |
|  |  |  |  |  | SMURF1 |
|  |  |  |  |  | SOAT2 |
|  |  |  |  |  | SOD1 |
|  |  |  |  |  | SOD2 |
|  |  |  |  |  | SOD3 |
|  |  |  |  |  | SORBS1 |
|  |  |  |  |  | SP1 |
|  |  |  |  |  | SP2 |
|  |  |  |  |  | SPAG5 |
|  |  |  |  |  | SPANXC |
|  |  |  |  |  | SPP1 |
|  |  |  |  |  | SRC |
|  |  |  |  |  | SRY |
|  |  |  |  |  | SST |
|  |  |  |  |  | STAT1 |
|  |  |  |  |  | STAT3 |
|  |  |  |  |  | STIM1 |
|  |  |  |  |  | STK39 |
|  |  |  |  |  | SUCLG2 |
|  |  |  |  |  | SUCNR1 |
|  |  |  |  |  | SULT1A1 |
|  |  |  |  |  | T |
|  |  |  |  |  | TAC1 |
|  |  |  |  |  | TACR1 |
|  |  |  |  |  | TAP1 |
|  |  |  |  |  | TBK1 |
|  |  |  |  |  | TBRG1 |
|  |  |  |  |  | TBXA2R |
|  |  |  |  |  | TBXAS1 |
|  |  |  |  |  | TF |
|  |  |  |  |  | TFRC |
|  |  |  |  |  | TG |
|  |  |  |  |  | TGFA |
|  |  |  |  |  | TGFB1 |
|  |  |  |  |  | TGFB3 |
|  |  |  |  |  | TGFBR1 |
|  |  |  |  |  | TGFBR2 |
|  |  |  |  |  | TGM2 |
|  |  |  |  |  | TH |
|  |  |  |  |  | THBD |
|  |  |  |  |  | THBS1 |
|  |  |  |  |  | THBS4 |
|  |  |  |  |  | THRA |
|  |  |  |  |  | TIMP1 |
|  |  |  |  |  | TIMP2 |
|  |  |  |  |  | TLR4 |
|  |  |  |  |  | TLX2 |
|  |  |  |  |  | TNC |
|  |  |  |  |  | TNF |
|  |  |  |  |  | TNFRSF10A |
|  |  |  |  |  | TNFRSF11B |
|  |  |  |  |  | TNFRSF1B |
|  |  |  |  |  | TNFRSF4 |
|  |  |  |  |  | TNNI3 |
|  |  |  |  |  | TPH1 |
|  |  |  |  |  | TPH2 |
|  |  |  |  |  | TRH |
|  |  |  |  |  | TRHR |
|  |  |  |  |  | TRPC5 |
|  |  |  |  |  | TRPC6 |
|  |  |  |  |  | TRPM4 |
|  |  |  |  |  | TRPM6 |
|  |  |  |  |  | TRPV1 |
|  |  |  |  |  | TXK |
|  |  |  |  |  | TXN |
|  |  |  |  |  | TXN2 |
|  |  |  |  |  | TXNL4B |
|  |  |  |  |  | UCN |
|  |  |  |  |  | UCP1 |
|  |  |  |  |  | UCP2 |
|  |  |  |  |  | UCP3 |
|  |  |  |  |  | UMOD |
|  |  |  |  |  | USP1 |
|  |  |  |  |  | USP2 |
|  |  |  |  |  | UTS2 |
|  |  |  |  |  | VAV2 |
|  |  |  |  |  | VCAM1 |
|  |  |  |  |  | VDR |
|  |  |  |  |  | VEGFA |
|  |  |  |  |  | VEGFB |
|  |  |  |  |  | VHL |
|  |  |  |  |  | VIM |
|  |  |  |  |  | VIP |
|  |  |  |  |  | VNN1 |
|  |  |  |  |  | VWF |
|  |  |  |  |  | WISP1 |
|  |  |  |  |  | WNK1 |
|  |  |  |  |  | WNK2 |
|  |  |  |  |  | WNK4 |
|  |  |  |  |  | WT1 |
|  |  |  |  |  | XDH |
|  |  |  |  |  | XYLT1 |
|  |  |  |  |  | XYLT2 |
|  |  |  |  |  | YEATS4 |
|  |  |  |  |  | YY1 |
|  |  |  |  |  | ZNF652 |

Table S4 Topological features of anti-hypertension targets related active compounds in SCD

| Molecule_ID | Molecule_Name | Degree | Herb |
| --- | --- | --- | --- |
| MOL000098 | quercetin | 41 | Herba Leonuri/Radix Glycyrrhizae/Spica Prunellae |
| MOL000422 | kaempferol | 31 | Herba Leonuri/Radix Paeoniae Alba/Radix Glycyrrhizae/Radix Gentianae/Spica Prunellae |
| MOL000449 | 8-Prenylwighteone | 28 | Spica Prunellae |
| MOL000358 | isorhamnetin | 28 | Radix Paeoniae Alba/Spica Prunellae |
| MOL003896 | 7-Methoxy-2-methyl isoflavone | 26 | Radix Glycyrrhizae |
| MOL002565 | Medicarpin | 24 | Radix Glycyrrhizae |
| MOL004978 | 2-[(3R)-8,8-dimethyl-3,4-dihydro-2H-pyrano[6,5-f]chromen-3-yl]-5-methoxyphenol | 21 | Radix Glycyrrhizae |
| MOL000392 | formononetin | 20 | Radix Glycyrrhizae |
| MOL005003 | Licoagrocarpin | 19 | Radix Glycyrrhizae |
| MOL000500 | Vestitol | 19 | Radix Glycyrrhizae |
| MOL004974 | 3'-Methoxyglabridin | 18 | Radix Glycyrrhizae |
| MOL004891 | shinpterocarpin | 18 | Radix Glycyrrhizae |
| MOL004835 | Glypallichalcone | 18 | Radix Glycyrrhizae |
| MOL004991 | 7-Acetoxy-2-methylisoflavone | 17 | Radix Glycyrrhizae |
| MOL004966 | 3'-Hydroxy-4'-O-Methylglabridin | 17 | Radix Glycyrrhizae |
| MOL000006 | molecule_name | 16 | Spica Prunellae |
| MOL001421 | p-xylene | 16 | Herba Leonuri |
| MOL005007 | Glyasperins M | 16 | Radix Glycyrrhizae |
| MOL004957 | HMO | 16 | Radix Glycyrrhizae |
| MOL004829 | Glepidotin B | 16 | Radix Glycyrrhizae |
| MOL000497 | licochalcone a | 16 | Radix Glycyrrhizae |
| MOL004959 | 1-Methoxyphaseollidin | 15 | Radix Glycyrrhizae |
| MOL004945 | (2S)-7-hydroxy-2-(4-hydroxyphenyl)-8-(3-methylbut-2-enyl)chroman-4-one | 15 | Radix Glycyrrhizae |
| MOL004833 | Phaseolinisoflavan | 15 | Radix Glycyrrhizae |
| MOL004828 | Glepidotin A | 15 | Radix Glycyrrhizae |
| MOL001484 | Inermine | 15 | Radix Glycyrrhizae |
| MOL001439 | p-xylene | 14 | Herba Leonuri |
| MOL001418 | p-xylene | 14 | Herba Leonuri |
| MOL004908 | Glabridin | 14 | Radix Glycyrrhizae |
| MOL004885 | licoisoflavanone | 14 | Radix Glycyrrhizae |
| MOL000354 | isorhamnetin | 14 | Herba Leonuri/Radix Glycyrrhizae |
| MOL004980 | Inflacoumarin A | 13 | Radix Glycyrrhizae |
| MOL004941 | (2R)-7-hydroxy-2-(4-hydroxyphenyl)chroman-4-one | 13 | Radix Glycyrrhizae |
| MOL004912 | Glabrone | 13 | Radix Glycyrrhizae |
| MOL004911 | Glabrene | 13 | Radix Glycyrrhizae |
| MOL004910 | Glabranin | 13 | Radix Glycyrrhizae |
| MOL004857 | Gancaonin B | 13 | Radix Glycyrrhizae |
| MOL004820 | kanzonols W | 13 | Radix Glycyrrhizae |
| MOL004815 | (E)-1-(2,4-dihydroxyphenyl)-3-(2,2-dimethylchromen-6-yl)prop-2-en-1-one | 13 | Radix Glycyrrhizae |
| MOL004811 | Glyasperin C | 13 | Radix Glycyrrhizae |
| MOL005020 | dehydroglyasperins C | 12 | Radix Glycyrrhizae |
| MOL004989 | 6-prenylated eriodictyol | 12 | Radix Glycyrrhizae |
| MOL004866 | 2-(3,4-dihydroxyphenyl)-5,7-dihydroxy-6-(3-methylbut-2-enyl)chromone | 12 | Radix Glycyrrhizae |
| MOL004856 | Gancaonin A | 12 | Radix Glycyrrhizae |
| MOL004849 | 3-(2,4-dihydroxyphenyl)-8-(1,1-dimethylprop-2-enyl)-7-hydroxy-5-methoxy-coumarin | 12 | Radix Glycyrrhizae |
| MOL004841 | Licochalcone B | 12 | Radix Glycyrrhizae |
| MOL004827 | Semilicoisoflavone B | 12 | Radix Glycyrrhizae |
| MOL004824 | (2S)-6-(2,4-dihydroxyphenyl)-2-(2-hydroxypropan-2-yl)-4-methoxy-2,3-dihydrofuro[3,2-g]chromen-7-one | 12 | Radix Glycyrrhizae |
| MOL004810 | glyasperin F | 12 | Radix Glycyrrhizae |
| MOL004808 | glyasperin B | 12 | Radix Glycyrrhizae |
| MOL004328 | naringenin | 12 | Radix Glycyrrhizae |
| MOL005012 | Licoagroisoflavone | 11 | Radix Glycyrrhizae |
| MOL005000 | Gancaonin G | 11 | Radix Glycyrrhizae |
| MOL004993 | 8-prenylated eriodictyol | 11 | Radix Glycyrrhizae |
| MOL004904 | licopyranocoumarin | 11 | Radix Glycyrrhizae |
| MOL004884 | Licoisoflavone B | 11 | Radix Glycyrrhizae |
| MOL004806 | euchrenone | 11 | Radix Glycyrrhizae |
| MOL004805 | (2S)-2-[4-hydroxy-3-(3-methylbut-2-enyl)phenyl]-8,8-dimethyl-2,3-dihydropyrano[2,3-f]chromen-4-one | 11 | Radix Glycyrrhizae |
| MOL003656 | Lupiwighteone | 11 | Radix Glycyrrhizae |
| MOL001792 | DFV | 11 | Radix Glycyrrhizae |
| MOL000417 | Calycosin | 11 | Radix Glycyrrhizae |
| MOL000737 | WLN: VH6 | 10 | Spica Prunellae |
| MOL003155 | BuOH | 10 | Radix Gentianae |
| MOL005016 | Odoratin | 10 | Radix Glycyrrhizae |
| MOL005008 | Glycyrrhiza flavonol A | 10 | Radix Glycyrrhizae |
| MOL004961 | Quercetin der. | 10 | Radix Glycyrrhizae |
| MOL004949 | Isolicoflavonol | 10 | Radix Glycyrrhizae |
| MOL004915 | Eurycarpin A | 10 | Radix Glycyrrhizae |
| MOL004879 | Glycyrin | 10 | Radix Glycyrrhizae |
| MOL004864 | 5,7-dihydroxy-3-(4-methoxyphenyl)-8-(3-methylbut-2-enyl)chromone | 10 | Radix Glycyrrhizae |
| MOL004855 | Licoricone | 10 | Radix Glycyrrhizae |
| MOL000239 | Jaranol | 10 | Radix Glycyrrhizae |
| MOL000492 | Prunetin | 10 | Radix Paeoniae Alba |
| MOL004935 | Sigmoidin-B | 9 | Radix Glycyrrhizae |
| MOL004903 | liquiritin | 9 | Radix Glycyrrhizae |
| MOL004848 | licochalcone G | 9 | Radix Glycyrrhizae |
| MOL004798 | beta-Terpinene | 8 | Spica Prunellae |
| MOL003170 | BuOH | 8 | Radix Gentianae |
| MOL003152 | BuOH | 8 | Radix Gentianae |
| MOL001558 | Vicenin-2 | 8 | Radix Gentianae |
| MOL001422 | p-xylene | 8 | Herba Leonuri |
| MOL005018 | Xambioona | 8 | Radix Glycyrrhizae |
| MOL004990 | 7,2',4'-trihydroxy－5-methoxy-3－arylcoumarin | 8 | Radix Glycyrrhizae |
| MOL004907 | Glyzaglabrin | 8 | Radix Glycyrrhizae |
| MOL004898 | (E)-3-[3,4-dihydroxy-5-(3-methylbut-2-enyl)phenyl]-1-(2,4-dihydroxyphenyl)prop-2-en-1-one | 8 | Radix Glycyrrhizae |
| MOL004883 | Licoisoflavone | 8 | Radix Glycyrrhizae |
| MOL004863 | 3-(3,4-dihydroxyphenyl)-5,7-dihydroxy-8-(3-methylbut-2-enyl)chromone | 8 | Radix Glycyrrhizae |
| MOL005017 | Phaseol | 7 | Radix Glycyrrhizae |
| MOL005001 | Gancaonin H | 6 | Radix Glycyrrhizae |
| MOL004988 | Kanzonol F | 6 | Radix Glycyrrhizae |
| MOL004838 | 8-(6-hydroxy-2-benzofuranyl)-2,2-dimethyl-5-chromenol | 6 | Radix Glycyrrhizae |
| MOL002322 | Glycyrol | 5 | Radix Gentianae |
| MOL001420 | p-xylene | 5 | Herba Leonuri |
| MOL004924 | (-)-Medicocarpin | 5 | Radix Glycyrrhizae |
| MOL004814 | Isotrifoliol | 5 | Radix Glycyrrhizae |
| MOL002311 | Glycyrol | 5 | Radix Glycyrrhizae |
| MOL006767 | naringin | 4 | Spica Prunellae |
| MOL004355 | naringenin | 4 | Spica Prunellae |
| MOL004996 | gadelaidic acid | 4 | Radix Glycyrrhizae |
| MOL004985 | icos-5-enoic acid | 4 | Radix Glycyrrhizae |
| MOL004948 | Isoglycyrol | 4 | Radix Glycyrrhizae |
| MOL004914 | 1,3-dihydroxy-8,9-dimethoxy-6-benzofurano[3,2-c]chromenone | 4 | Radix Glycyrrhizae |
| MOL004882 | Licocoumarone | 4 | Radix Glycyrrhizae |
| MOL001919 | Izoforon | 4 | Radix Paeoniae Alba |
| MOL000359 | sitosterol | 4 | Radix Paeoniae Alba/Radix Glycyrrhizae/Radix Gentianae |
| MOL006772 | naringin | 3 | Spica Prunellae |
| MOL004913 | 1,3-dihydroxy-9-methoxy-6-benzofurano[3,2-c]chromenone | 3 | Radix Glycyrrhizae |
| MOL000211 | Mairin | 3 | Radix Paeoniae Alba/Radix Glycyrrhizae |
| MOL003137 | BuOH | 2 | Radix Gentianae |
| MOL005013 | 18α-hydroxyglycyrrhetic acid | 2 | Radix Glycyrrhizae |
| MOL006774 | naringin | 1 | Spica Prunellae |
| MOL001924 | Izoforon | 1 | Radix Paeoniae Alba |

Table S5 Topological features of 116 candidate targets

| Gene_Name | BC | DC | EC | CC | NC | LAC |
| --- | --- | --- | --- | --- | --- | --- |
| ABL1 | 2073.656 | 234 | 0.028296 | 0.52983 | 36.02132 | 24.40625 |
| ACTB | 2952.617 | 248 | 0.041934 | 0.538499 | 44.55552 | 29.09877 |
| AKT1 | 2591.916 | 277 | 0.030729 | 0.534384 | 37.69817 | 25.12925 |
| APP | 13238.49 | 1096 | 0.057084 | 0.567157 | 78.8549 | 29.16854 |
| AR | 3753.073 | 352 | 0.032974 | 0.539797 | 51.0164 | 28.1369 |
| ARRB2 | 3450.635 | 285 | 0.053889 | 0.547724 | 53.17191 | 30.905 |
| BMI1 | 1576.282 | 224 | 0.033895 | 0.530835 | 32.0093 | 23.87681 |
| BRCA1 | 6530.947 | 364 | 0.049647 | 0.55644 | 76.076 | 32.9 |
| CALM1 | 2228.102 | 382 | 0.034081 | 0.534895 | 37.84654 | 25.57047 |
| CALM2 | 2228.102 | 382 | 0.034081 | 0.534895 | 37.84654 | 25.57047 |
| CALM3 | 2228.102 | 382 | 0.034081 | 0.534895 | 37.84654 | 25.57047 |
| CAND1 | 5992.272 | 447 | 0.08731 | 0.574435 | 138.3955 | 57.58503 |
| CCDC8 | 6274.996 | 413 | 0.06585 | 0.565437 | 73.9829 | 31.98851 |
| CDC5L | 6059.328 | 389 | 0.060125 | 0.56006 | 85.87131 | 38.95473 |
| CDK1 | 1915.745 | 234 | 0.034296 | 0.52983 | 36.14376 | 26.18657 |
| CDK2 | 16357.92 | 578 | 0.090089 | 0.597756 | 148.1726 | 44.04632 |
| CLTC | 3080.148 | 236 | 0.040556 | 0.537464 | 47.18495 | 28.42138 |
| COMMD3-BMI1 | 1576.282 | 224 | 0.033895 | 0.530835 | 32.0093 | 23.87681 |
| COPS5 | 8753.259 | 508 | 0.092548 | 0.585864 | 145.8623 | 53.90881 |
| CREBBP | 3063.462 | 296 | 0.036119 | 0.53824 | 63.65877 | 36.24561 |
| CRK | 1392.679 | 227 | 0.022084 | 0.521435 | 28.45939 | 20.33654 |
| CSNK2A1 | 1982.561 | 265 | 0.041434 | 0.536691 | 43.49173 | 29.91083 |
| CSNK2A3 | 1982.561 | 265 | 0.041434 | 0.536691 | 43.49173 | 29.91083 |
| CTNNB1 | 5384.515 | 298 | 0.039655 | 0.545322 | 50.92915 | 25.43316 |
| CUL1 | 9174.744 | 482 | 0.091963 | 0.585251 | 146.5262 | 54.80428 |
| CUL2 | 3625.599 | 317 | 0.06761 | 0.556163 | 75.80027 | 40.47598 |
| CUL3 | 21980.02 | 746 | 0.112088 | 0.62514 | 223.2686 | 55.49889 |
| CUL4B | 1839.976 | 225 | 0.052709 | 0.539797 | 54.59301 | 38.82249 |
| CUL5 | 2650.928 | 285 | 0.063693 | 0.549877 | 70.72793 | 42.67476 |
| CUL7 | 11263.35 | 516 | 0.089423 | 0.594896 | 137.5462 | 42.40782 |
| EED | 5102.608 | 353 | 0.069229 | 0.563161 | 92.51078 | 42.27559 |
| EEF1A1 | 4780.58 | 314 | 0.069172 | 0.556163 | 83.56022 | 46.71179 |
| EGFR | 13317.28 | 754 | 0.057234 | 0.57473 | 110.652 | 34.75342 |
| EP300 | 6746.502 | 394 | 0.052589 | 0.55922 | 97.24061 | 41.21667 |
| ESR1 | 18882.41 | 655 | 0.089313 | 0.599357 | 161.4259 | 47.05094 |
| EWSR1 | 5770.875 | 373 | 0.052883 | 0.551775 | 63.89007 | 31.83099 |
| FBXO6 | 5816.718 | 406 | 0.059012 | 0.56006 | 63.2766 | 27.50622 |
| FLNA | 2676.772 | 255 | 0.041068 | 0.536691 | 52.6308 | 32.32278 |
| FN1 | 14858.62 | 637 | 0.098175 | 0.604212 | 164.6199 | 47.36951 |
| FUS | 3243.07 | 267 | 0.060553 | 0.549337 | 70.14472 | 42.96059 |
| GRB2 | 7550.957 | 498 | 0.053289 | 0.557271 | 82.46437 | 36.01732 |
| GSK3B | 1856.076 | 236 | 0.023009 | 0.524122 | 24.57385 | 18.06087 |
| HDAC1 | 3996.132 | 340 | 0.04438 | 0.548261 | 80.02582 | 40.31343 |
| HDAC2 | 2077.513 | 228 | 0.039335 | 0.535407 | 56.55155 | 37.14013 |
| HDAC5 | 5277.048 | 335 | 0.06655 | 0.563161 | 80.1464 | 37.30435 |
| HIST1H3A | 2243.314 | 299 | 0.058513 | 0.553139 | 94.78935 | 49.27149 |
| HIST1H3B | 2243.314 | 299 | 0.058513 | 0.553139 | 94.78935 | 49.27149 |
| HIST1H3C | 2243.314 | 299 | 0.058513 | 0.553139 | 94.78935 | 49.27149 |
| HIST1H3D | 2243.314 | 299 | 0.058513 | 0.553139 | 94.78935 | 49.27149 |
| HIST1H3E | 2243.314 | 299 | 0.058513 | 0.553139 | 94.78935 | 49.27149 |
| HIST1H3F | 2243.314 | 299 | 0.058513 | 0.553139 | 94.78935 | 49.27149 |
| HIST1H3G | 2243.314 | 299 | 0.058513 | 0.553139 | 94.78935 | 49.27149 |
| HIST1H3H | 2243.314 | 299 | 0.058513 | 0.553139 | 94.78935 | 49.27149 |
| HIST1H3I | 2243.314 | 299 | 0.058513 | 0.553139 | 94.78935 | 49.27149 |
| HIST1H3J | 2243.314 | 299 | 0.058513 | 0.553139 | 94.78935 | 49.27149 |
| HNRNPA1 | 3637.919 | 353 | 0.069599 | 0.556163 | 98.85389 | 54.10965 |
| HNRNPU | 5618.83 | 359 | 0.08286 | 0.567157 | 132.8304 | 62.01115 |
| HSP90AA1 | 10880.94 | 510 | 0.069474 | 0.575617 | 123.1948 | 44.88814 |
| HSP90AB1 | 7656.348 | 427 | 0.064204 | 0.564297 | 93.47498 | 40.77734 |
| HSPA4 | 3248.203 | 242 | 0.042704 | 0.54058 | 46.36127 | 29.33929 |
| HSPA5 | 7329.547 | 385 | 0.067911 | 0.564012 | 97.47353 | 44.35156 |
| HSPA8 | 4687.261 | 344 | 0.060173 | 0.556716 | 83.1319 | 42.24017 |
| HSPB1 | 2003.876 | 246 | 0.037236 | 0.531087 | 38.1704 | 28.38406 |
| HUWE1 | 7330.847 | 362 | 0.067432 | 0.567444 | 95.67031 | 39.5539 |
| IKBKE | 3441.343 | 301 | 0.035775 | 0.539797 | 33.66644 | 18.8869 |
| IKBKG | 3025.254 | 285 | 0.037539 | 0.537981 | 46.02634 | 28.46296 |
| ILF3 | 1537.796 | 226 | 0.060678 | 0.538758 | 65.70144 | 51.88623 |
| ITGA4 | 8578.77 | 471 | 0.084921 | 0.583116 | 119.5056 | 44.0875 |
| JUN | 1826.878 | 278 | 0.025224 | 0.528079 | 35.30796 | 24.83333 |
| LRRK2 | 2019.167 | 218 | 0.032326 | 0.529579 | 28.13583 | 21.18939 |
| MAPK1 | 1949.823 | 276 | 0.028549 | 0.529579 | 37.35194 | 25.33083 |
| MCM2 | 17792.85 | 627 | 0.105613 | 0.612144 | 193.2651 | 54.78832 |
| MDM2 | 5623.851 | 336 | 0.048375 | 0.550147 | 59.76312 | 30.37681 |
| MYC | 7597.264 | 420 | 0.050764 | 0.560341 | 70.85183 | 29.36364 |
| MYH9 | 2382.112 | 257 | 0.037539 | 0.535663 | 51.92459 | 31.26797 |
| NPM1 | 9635.612 | 448 | 0.096393 | 0.584029 | 161.5907 | 64.28483 |
| NTRK1 | 42789.39 | 1205 | 0.117286 | 0.660567 | 288.3564 | 52.2844 |
| OBSL1 | 7148.986 | 440 | 0.077046 | 0.578295 | 105.7583 | 38.08553 |
| PAN2 | 3828.375 | 295 | 0.062026 | 0.552047 | 61.51504 | 33.94931 |
| PARK2 | 6367.807 | 344 | 0.054 | 0.556994 | 68.29404 | 30.0087 |
| PARP1 | 2210.795 | 224 | 0.045603 | 0.53592 | 50.20836 | 36.58228 |
| PRKDC | 2561.071 | 228 | 0.046683 | 0.537981 | 45.06906 | 31.22424 |
| RB1 | 2091.219 | 232 | 0.027534 | 0.529328 | 36.93104 | 25.21053 |
| RELA | 2831.735 | 243 | 0.032418 | 0.534639 | 43.06181 | 27.33775 |
| RNF2 | 9959.804 | 473 | 0.074233 | 0.578295 | 108.2545 | 37.95082 |
| RPA1 | 4390.951 | 330 | 0.055959 | 0.551503 | 68.0717 | 35.63256 |
| RPA2 | 3511.883 | 309 | 0.050821 | 0.547456 | 52.45729 | 29.46939 |
| RPA3 | 2192.439 | 267 | 0.045168 | 0.540319 | 45.0315 | 28.83041 |
| RPS27A | 3037.391 | 244 | 0.050637 | 0.540058 | 53.55727 | 36.97688 |
| SIRT7 | 7107.218 | 456 | 0.067539 | 0.566582 | 82.74584 | 34.37687 |
| SMAD2 | 2173.071 | 272 | 0.026943 | 0.529579 | 31.21864 | 21.10769 |
| SMAD3 | 2392.781 | 280 | 0.02798 | 0.53235 | 40.15121 | 25.20863 |
| SMURF1 | 3960.015 | 269 | 0.045906 | 0.545056 | 43.17304 | 24.16578 |
| SNW1 | 6888.205 | 353 | 0.054569 | 0.558105 | 78.98291 | 35.22128 |
| SRC | 3363.554 | 321 | 0.027203 | 0.535663 | 49.75035 | 26.50993 |
| SRPK2 | 1373.365 | 221 | 0.025933 | 0.522897 | 26.23723 | 20.61468 |
| STAU1 | 3039.358 | 255 | 0.055909 | 0.547188 | 59.78137 | 36.02551 |
| SUZ12 | 3470.705 | 270 | 0.045807 | 0.545854 | 55.00068 | 30.27692 |
| TARDBP | 1763.147 | 237 | 0.059667 | 0.540841 | 72.71184 | 50.10497 |
| TP53 | 25160.8 | 747 | 0.095607 | 0.612815 | 196.59 | 51.05811 |
| TRAF6 | 7605.034 | 397 | 0.042822 | 0.555335 | 63.28851 | 25.45089 |
| TUBB | 2553.822 | 226 | 0.048383 | 0.541889 | 50.53795 | 32.67232 |
| U2AF2 | 4702.742 | 285 | 0.059012 | 0.555887 | 85.26877 | 41.15419 |
| UBC | 15071.38 | 574 | 0.072986 | 0.584639 | 139.9349 | 44.03988 |
| UBE2I | 4991.556 | 336 | 0.041019 | 0.545854 | 53.17903 | 27.20419 |
| UBL4A | 2594.888 | 236 | 0.05337 | 0.541103 | 57.85346 | 39.84746 |
| VCAM1 | 7100.992 | 452 | 0.079302 | 0.576804 | 101.6432 | 39.17391 |
| VCP | 6575.68 | 423 | 0.061178 | 0.5595 | 84.99034 | 40.31799 |
| VHL | 5100.23 | 349 | 0.055846 | 0.55232 | 56.5339 | 30.21759 |
| XPO1 | 10398.56 | 624 | 0.06294 | 0.567732 | 91.98576 | 35.47037 |
| XRCC6 | 2144.346 | 218 | 0.043333 | 0.533874 | 43.20322 | 31.11333 |
| YWHAB | 1749.938 | 251 | 0.032408 | 0.528828 | 35.7431 | 26.72656 |
| YWHAE | 2804.564 | 280 | 0.051664 | 0.542151 | 57.40661 | 38.28652 |
| YWHAG | 2873.103 | 275 | 0.04507 | 0.540319 | 52.25113 | 33.67857 |
| YWHAQ | 4550.907 | 337 | 0.053026 | 0.550147 | 61.82703 | 34.19417 |
| YWHAZ | 8656.936 | 466 | 0.076201 | 0.574141 | 121.7417 | 50.17931 |
